# Supplementary material for: Development of a Train-the-Trainer Quality Improvement Curriculum
Source: MedEdPORTAL. 2024 Jul 16;20:11425. doi: 10.15766/mep_2374-8265.11425 (PMC11249715; doi:10.15766/mep_2374-8265.11425)
Supplement: Supplementary file 1 — Train-the-Trainer Slide Set.pptxExercise 1 Aim Statements.docxExercise 2 Stakeholder Analysis.docxExercise 3a Flowchart Critique.docxExercise 3b Fishbone Critique.docxExercise 4 Measures Critique.docxExercise 5 Intervention Critique.docxExercise 1 Aim Statements Facilitator Guide.docxExercise 2 Stakeholder Analysis Facilitator Guide.docxExercise 3a Flowchart Critique Facilitator Guide.docxExercise 3b Fishbone Critique Facilitator Guide.docxExercise 4 Measures Critique Facilitator Guide.docxExercise 5 Intervention Critique Facilitator Guide.docxTrain-the-Trainer Quality Preassessment.docxCourse Evaluation.docxTrain-the-Trainer Quality Postassessment.doc [file mep_2374-8265.11425-s001.zip › O. Course Evaluation.docx]

**How to Teach QI: A Train the Trainer Workshop**

**Course Evaluation**

Workshop Content:

| 1= very poor | 2 = poor | 3 = good | 4 = very good | 5 = excellent |
| --- | --- | --- | --- | --- |

| 1. The workshop was well organized. | 1 | 2 | 3 | 4 | 5 |
| --- | --- | --- | --- | --- | --- |
| 1. Objectives were stated clearly. | 1 | 2 | 3 | 4 | 5 |
| 1. The topics covered in the workshop met my expectations. | 1 | 2 | 3 | 4 | 5 |
| 1. The handouts were helpful in class or for future use. | 1 | 2 | 3 | 4 | 5 |
| 1. The exercises/lessons were a helpful learning experience. | 1 | 2 | 3 | 4 | 5 |

Instructor(s):

| 1= very poor | 2 = poor | 3 = good | 4 = very good | 5 = excellent |
| --- | --- | --- | --- | --- |

| 1. The instructor(s) presented the material clearly. | 1 | 2 | 3 | 4 | 5 |
| --- | --- | --- | --- | --- | --- |
| 1. The instructor(s) were prepared to teach the course. | 1 | 2 | 3 | 4 | 5 |
| 1. The exchange of ideas was allowed and encouraged. | 1 | 2 | 3 | 4 | 5 |

Overall rating:

| 1= very poor | 2 = poor | 3 = good | 4 = very good | | 5 = excellent | | | | |
| --- | --- | --- | --- | --- | --- | --- | --- | --- | --- |
| My overall rating for the workshop is: | | | | 1 | | 2 | 3 | 4 | 5 |

**Comments or suggestions for course improvement:**
