# Supplementary material for: Development of a Train-the-Trainer Quality Improvement Curriculum
Source: MedEdPORTAL. 2024 Jul 16;20:11425. doi: 10.15766/mep_2374-8265.11425 (PMC11249715; doi:10.15766/mep_2374-8265.11425)
Supplement: Supplementary file 1 — Train-the-Trainer Slide Set.pptxExercise 1 Aim Statements.docxExercise 2 Stakeholder Analysis.docxExercise 3a Flowchart Critique.docxExercise 3b Fishbone Critique.docxExercise 4 Measures Critique.docxExercise 5 Intervention Critique.docxExercise 1 Aim Statements Facilitator Guide.docxExercise 2 Stakeholder Analysis Facilitator Guide.docxExercise 3a Flowchart Critique Facilitator Guide.docxExercise 3b Fishbone Critique Facilitator Guide.docxExercise 4 Measures Critique Facilitator Guide.docxExercise 5 Intervention Critique Facilitator Guide.docxTrain-the-Trainer Quality Preassessment.docxCourse Evaluation.docxTrain-the-Trainer Quality Postassessment.doc [file mep_2374-8265.11425-s001.zip › A. Train-the-Trainer Slide Set.pptx]

## Slide 1
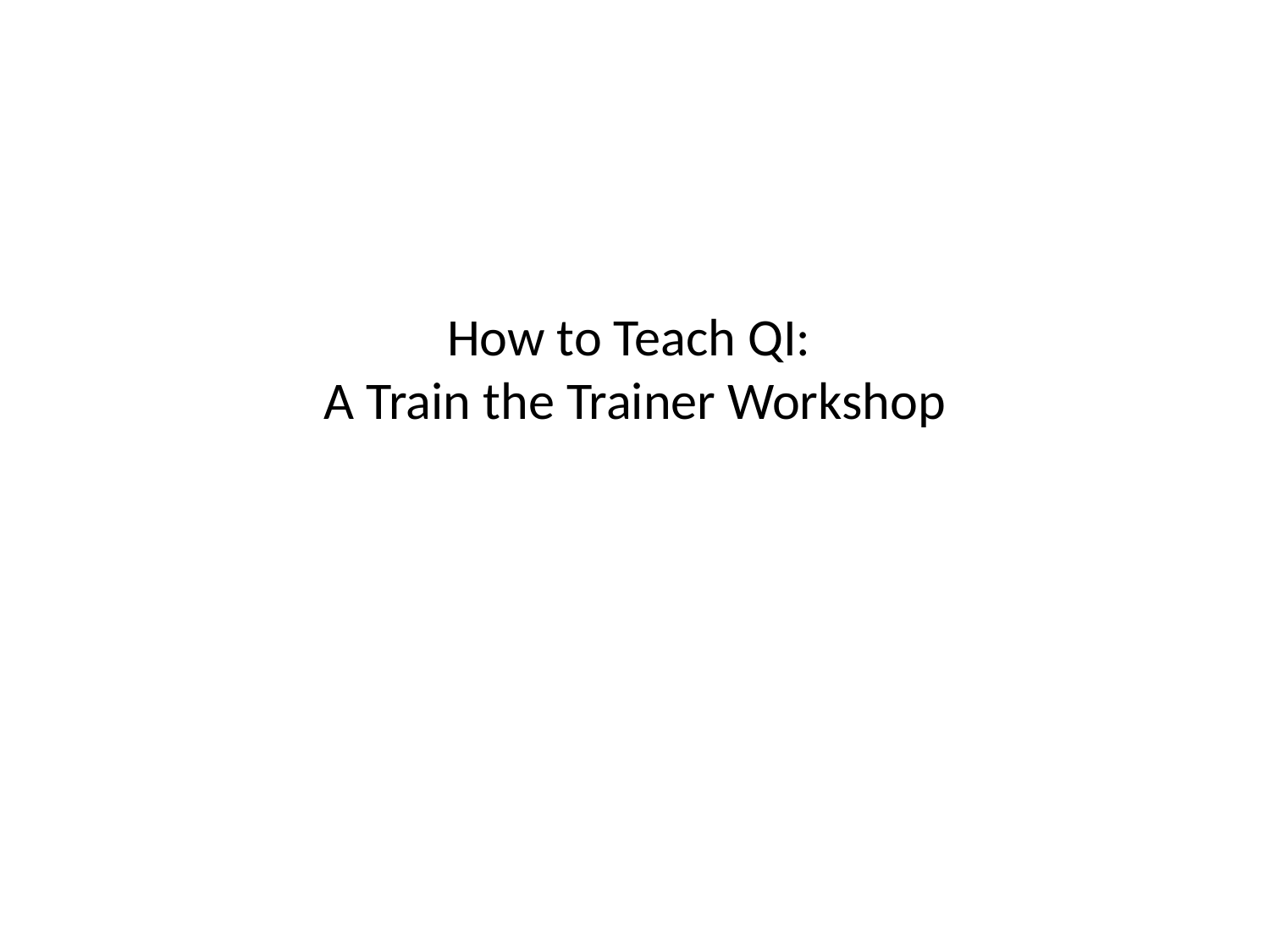

# How to Teach QI: A Train the Trainer Workshop

## Slide 2
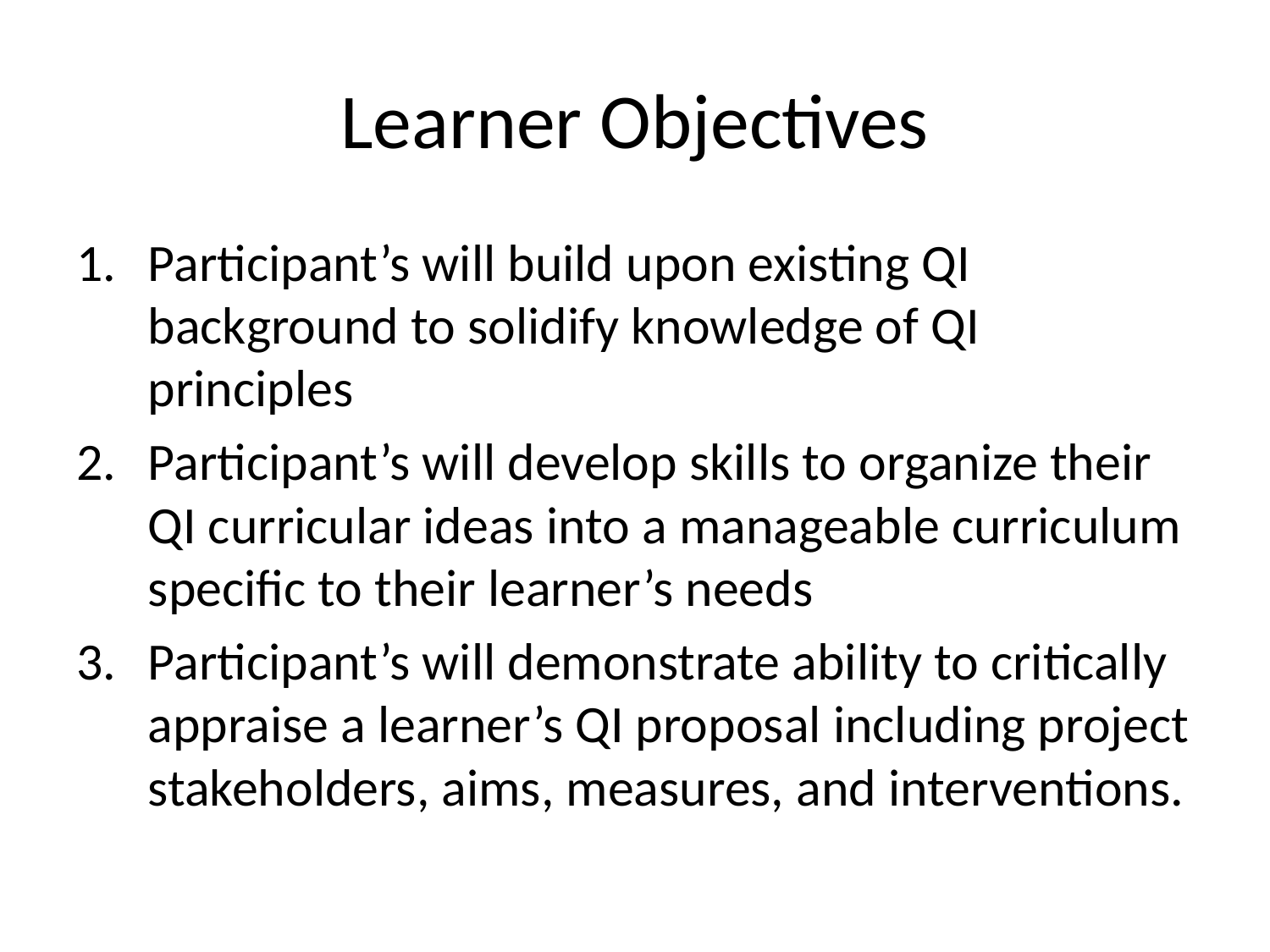

# Learner Objectives
Participant’s will build upon existing QI background to solidify knowledge of QI principles
Participant’s will develop skills to organize their QI curricular ideas into a manageable curriculum specific to their learner’s needs
Participant’s will demonstrate ability to critically appraise a learner’s QI proposal including project stakeholders, aims, measures, and interventions.

## Slide 3
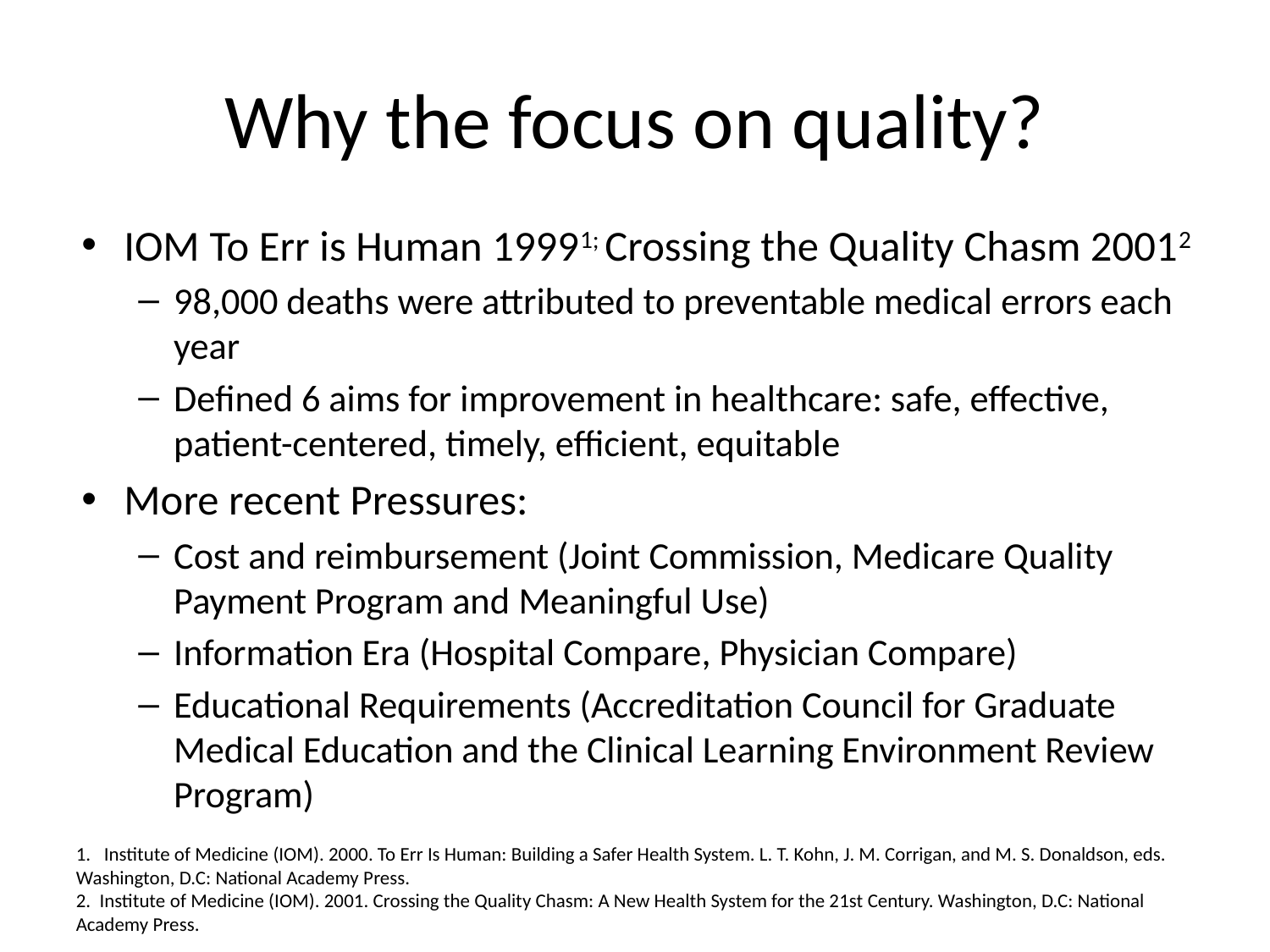

# Why the focus on quality?
IOM To Err is Human 19991; Crossing the Quality Chasm 20012
98,000 deaths were attributed to preventable medical errors each year
Defined 6 aims for improvement in healthcare: safe, effective, patient-centered, timely, efficient, equitable
More recent Pressures:
Cost and reimbursement (Joint Commission, Medicare Quality Payment Program and Meaningful Use)
Information Era (Hospital Compare, Physician Compare)
Educational Requirements (Accreditation Council for Graduate Medical Education and the Clinical Learning Environment Review Program)
1. Institute of Medicine (IOM). 2000. To Err Is Human: Building a Safer Health System. L. T. Kohn, J. M. Corrigan, and M. S. Donaldson, eds. Washington, D.C: National Academy Press.
2. Institute of Medicine (IOM). 2001. Crossing the Quality Chasm: A New Health System for the 21st Century. Washington, D.C: National Academy Press.

## Slide 4
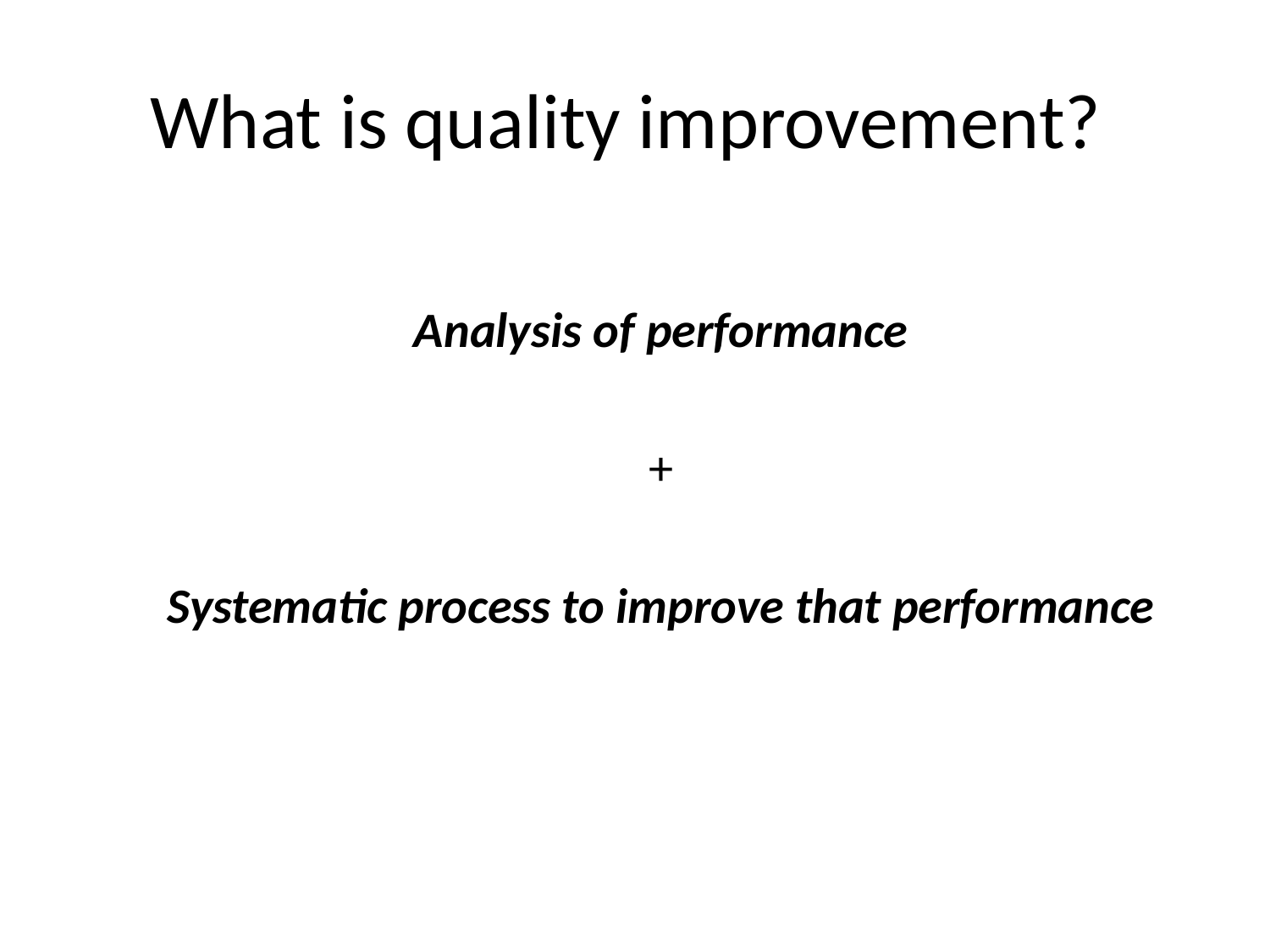

# What is quality improvement?
Analysis of performance
+
Systematic process to improve that performance

## Slide 5
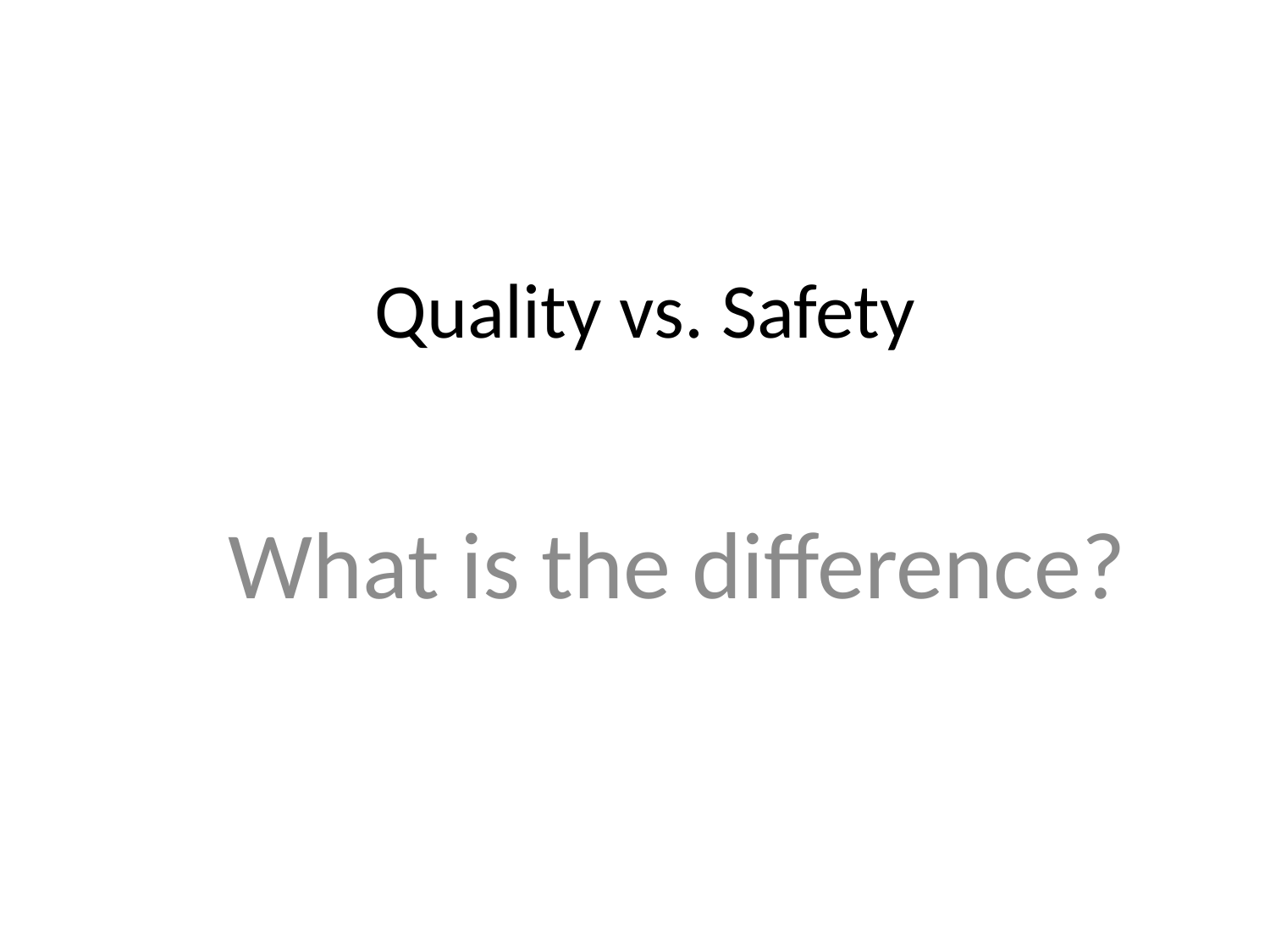

# Quality vs. Safety
What is the difference?

## Slide 6
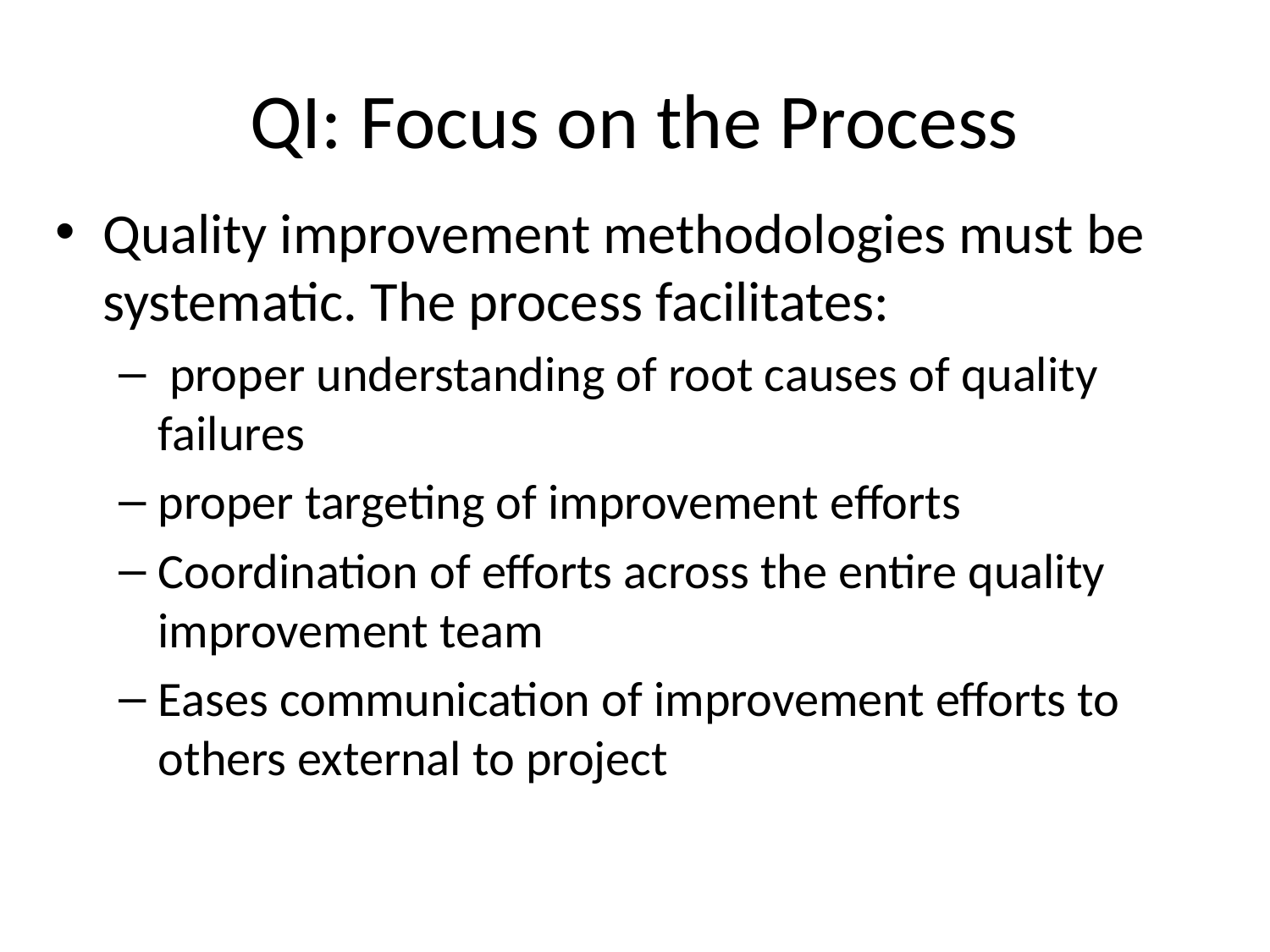

# QI: Focus on the Process
Quality improvement methodologies must be systematic. The process facilitates:
 proper understanding of root causes of quality failures
proper targeting of improvement efforts
Coordination of efforts across the entire quality improvement team
Eases communication of improvement efforts to others external to project

## Slide 7
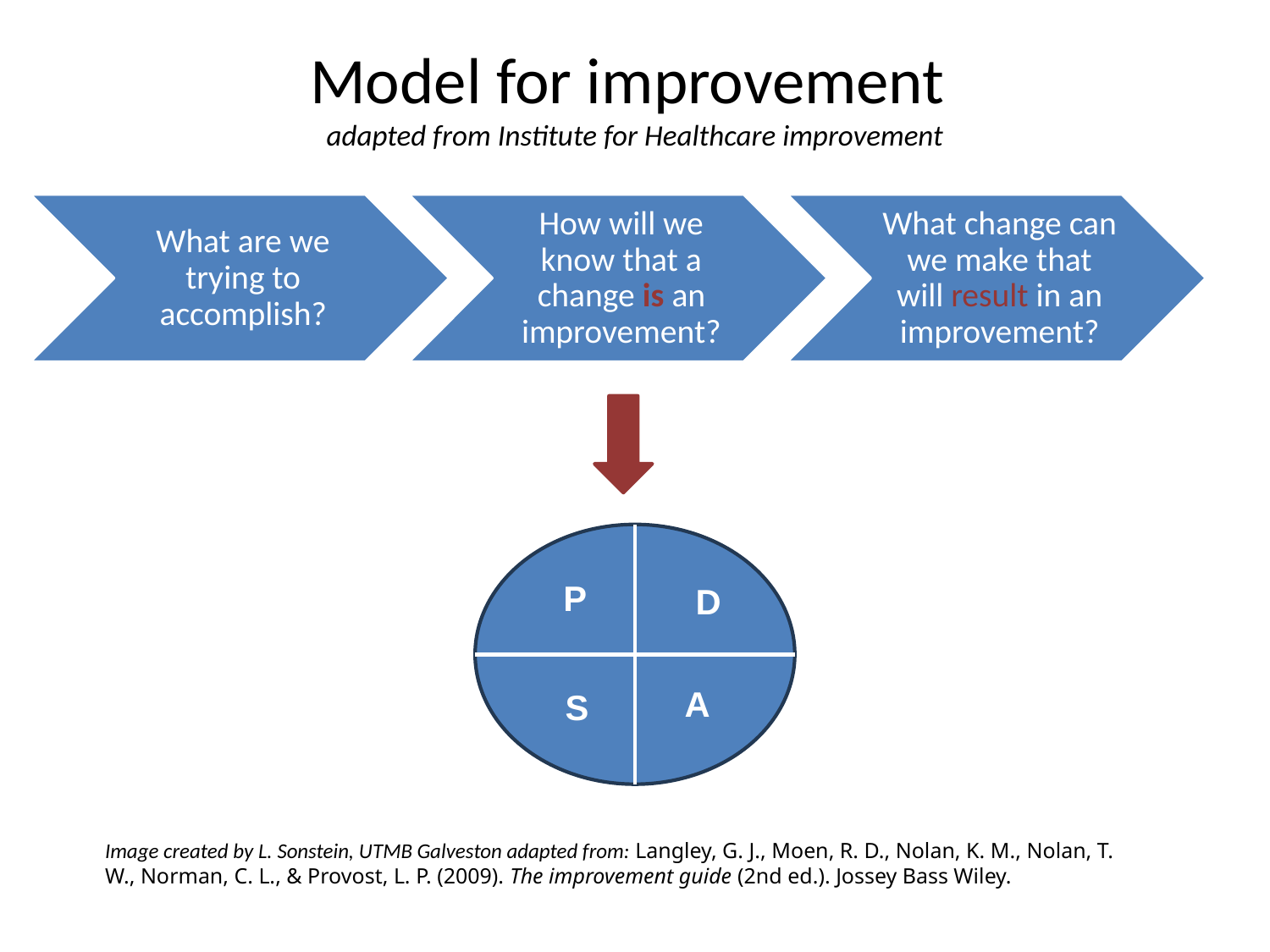

# Model for improvement  adapted from Institute for Healthcare improvement
P
D
A
S
Image created by L. Sonstein, UTMB Galveston adapted from: Langley, G. J., Moen, R. D., Nolan, K. M., Nolan, T. W., Norman, C. L., & Provost, L. P. (2009). The improvement guide (2nd ed.). Jossey Bass Wiley.

## Slide 8
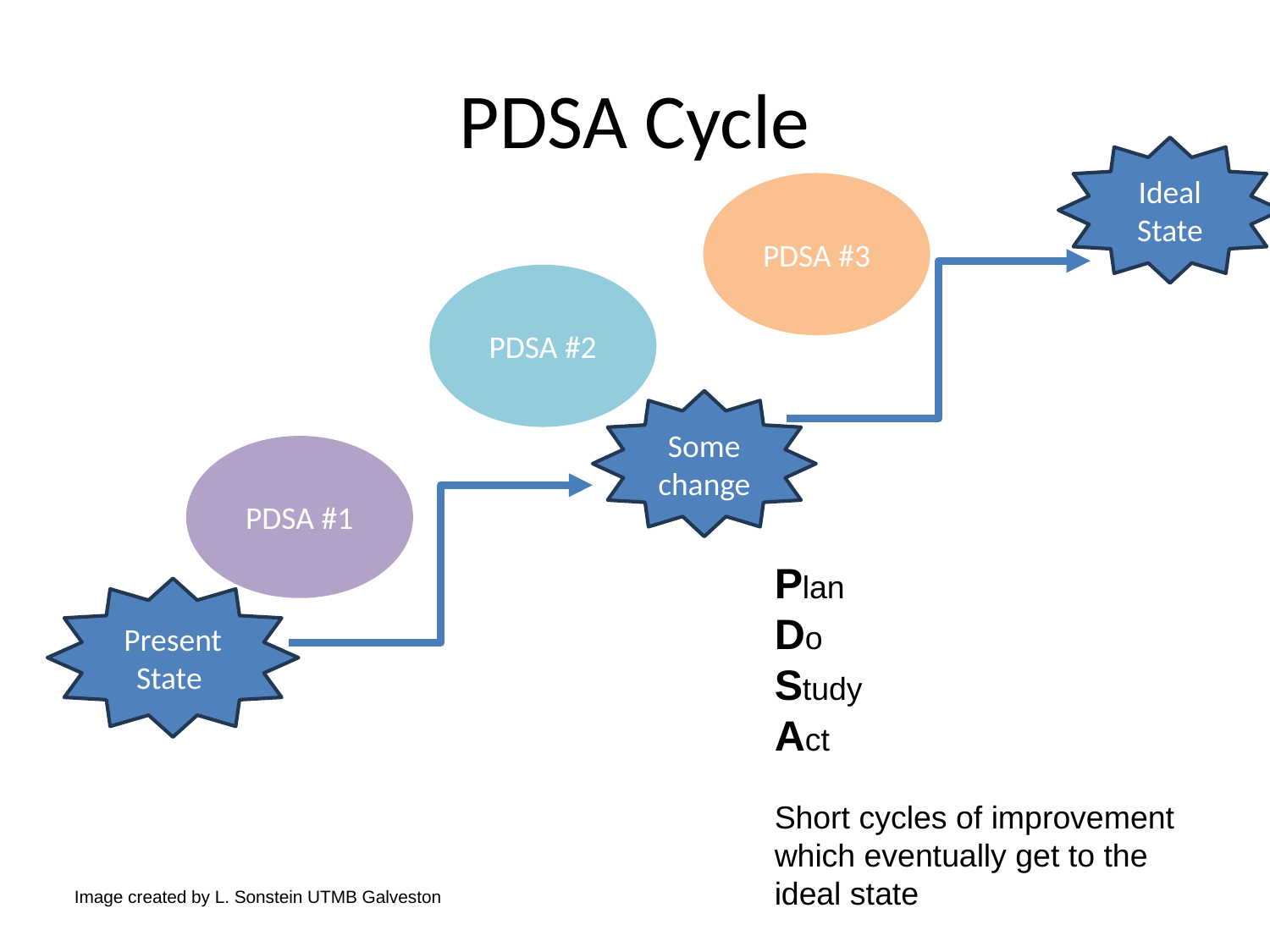

# PDSA Cycle
Ideal State
PDSA #3
PDSA #2
Some change
PDSA #1
Plan
Do
Study
Act
Short cycles of improvement which eventually get to the ideal state
Present State
Image created by L. Sonstein UTMB Galveston

## Slide 9
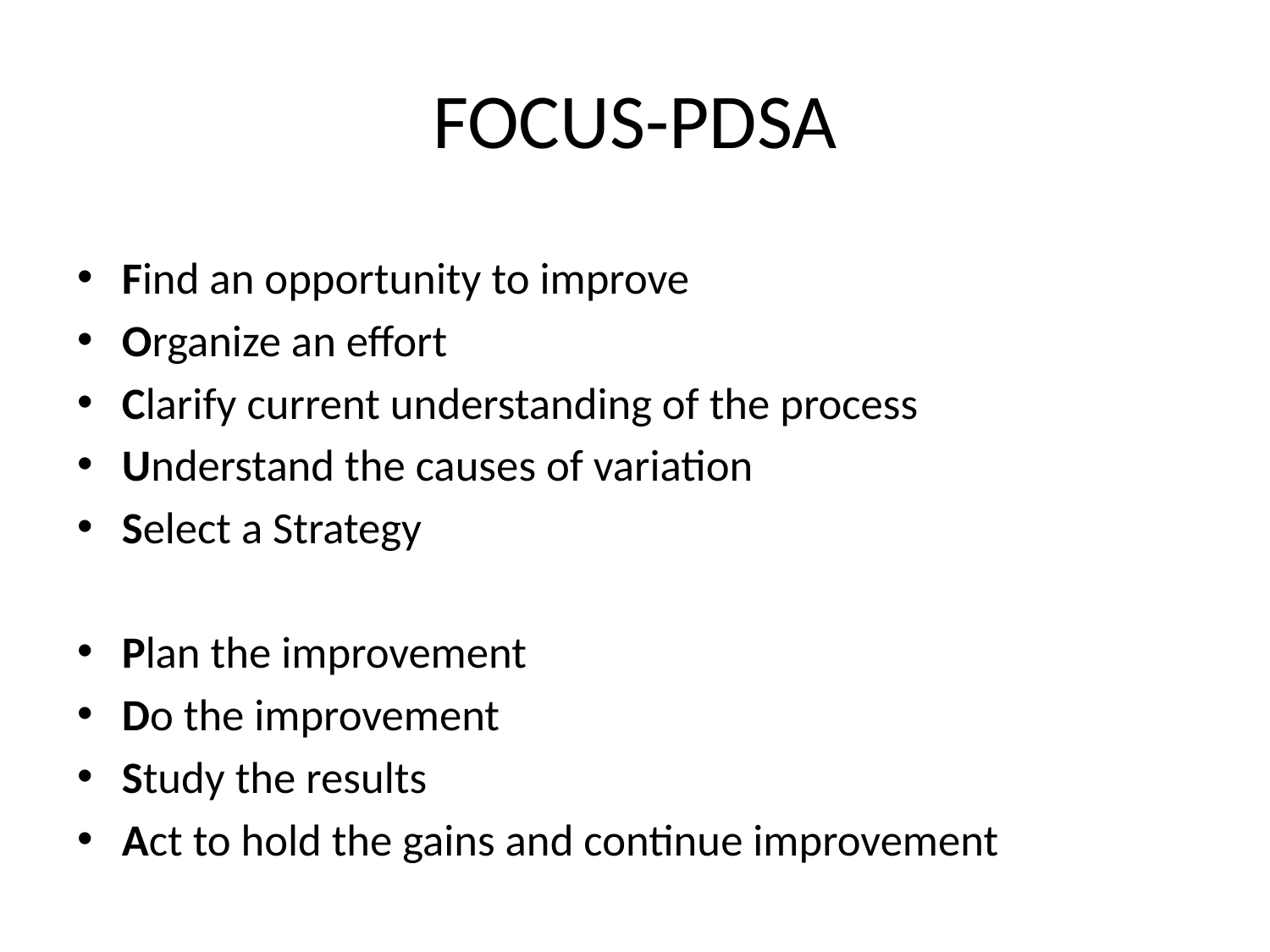

# FOCUS-PDSA
Find an opportunity to improve
Organize an effort
Clarify current understanding of the process
Understand the causes of variation
Select a Strategy
Plan the improvement
Do the improvement
Study the results
Act to hold the gains and continue improvement

## Slide 10
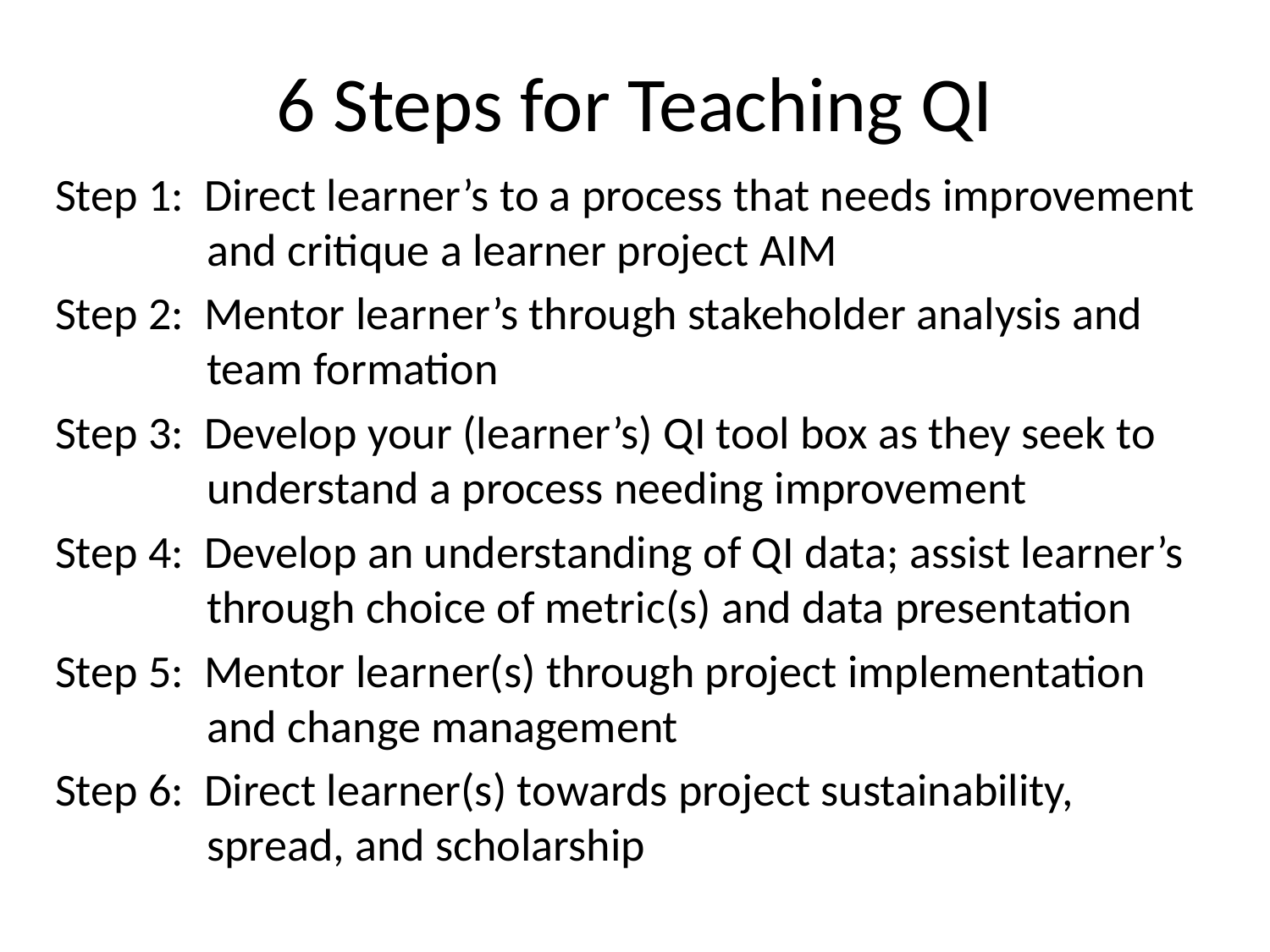

# 6 Steps for Teaching QI
Step 1: Direct learner’s to a process that needs improvement and critique a learner project AIM
Step 2: Mentor learner’s through stakeholder analysis and team formation
Step 3: Develop your (learner’s) QI tool box as they seek to understand a process needing improvement
Step 4: Develop an understanding of QI data; assist learner’s through choice of metric(s) and data presentation
Step 5: Mentor learner(s) through project implementation and change management
Step 6: Direct learner(s) towards project sustainability, spread, and scholarship

## Slide 11
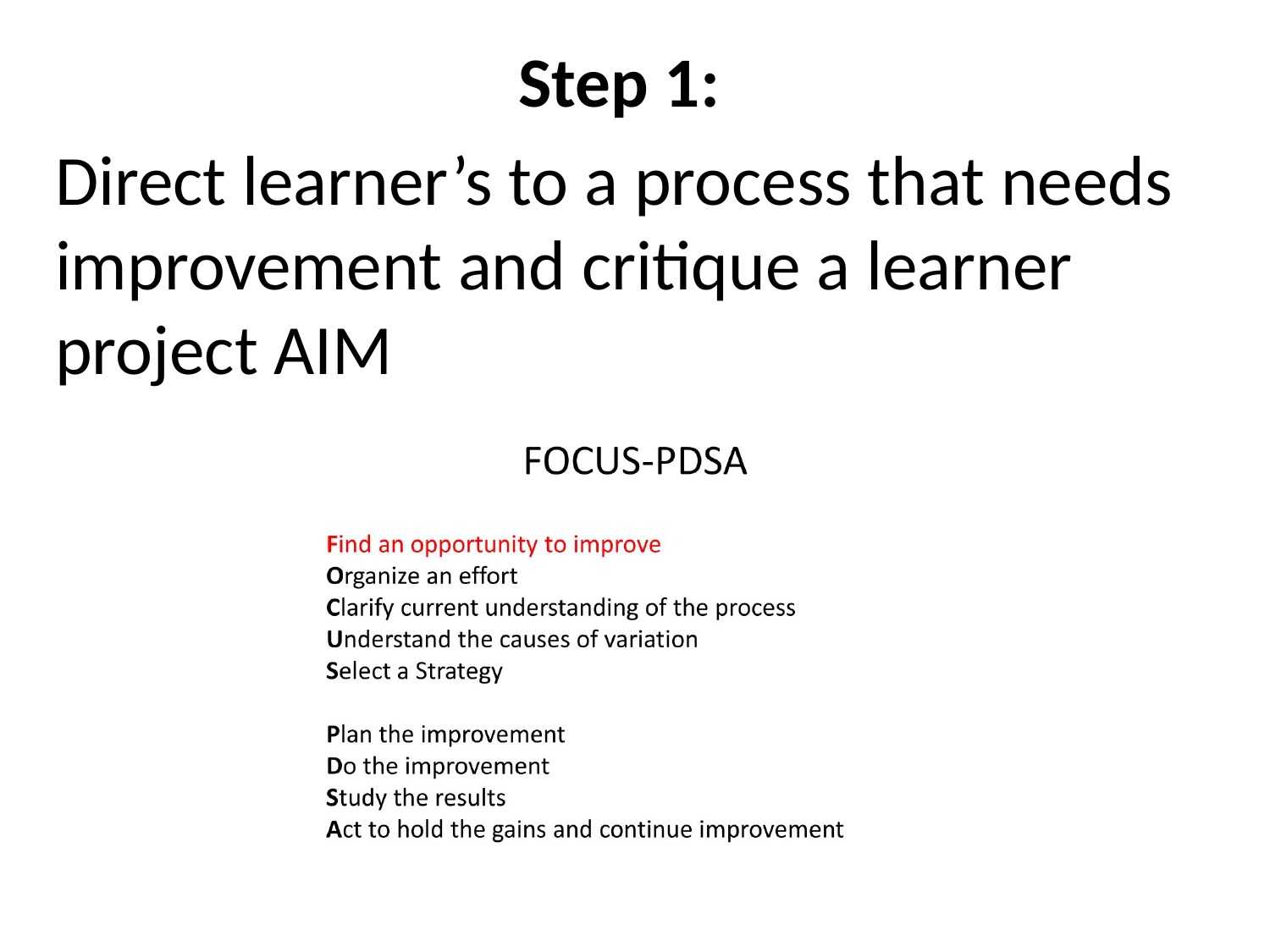

Step 1:
Direct learner’s to a process that needs improvement and critique a learner project AIM

## Slide 12
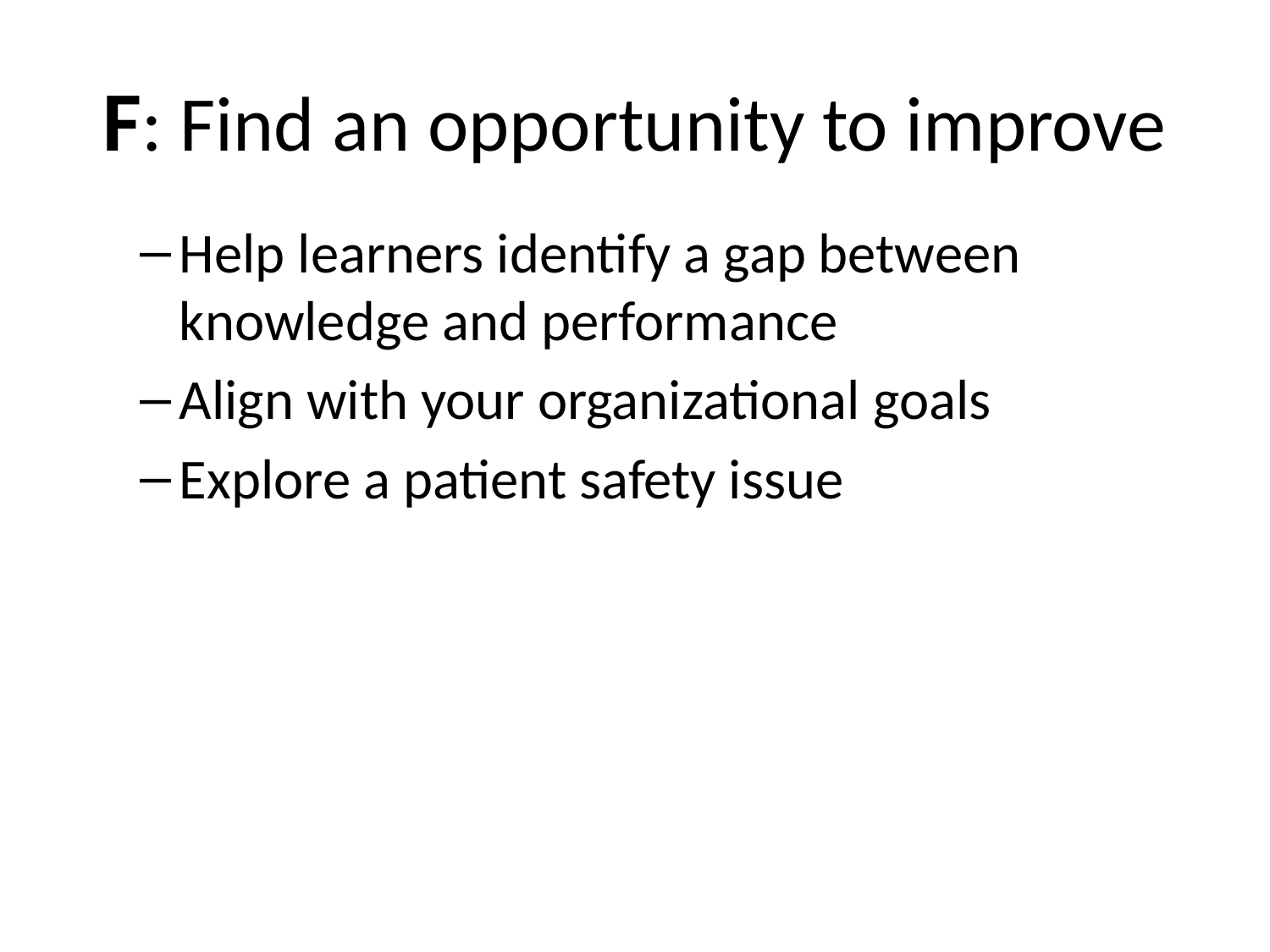

# F: Find an opportunity to improve
Help learners identify a gap between knowledge and performance
Align with your organizational goals
Explore a patient safety issue

## Slide 13
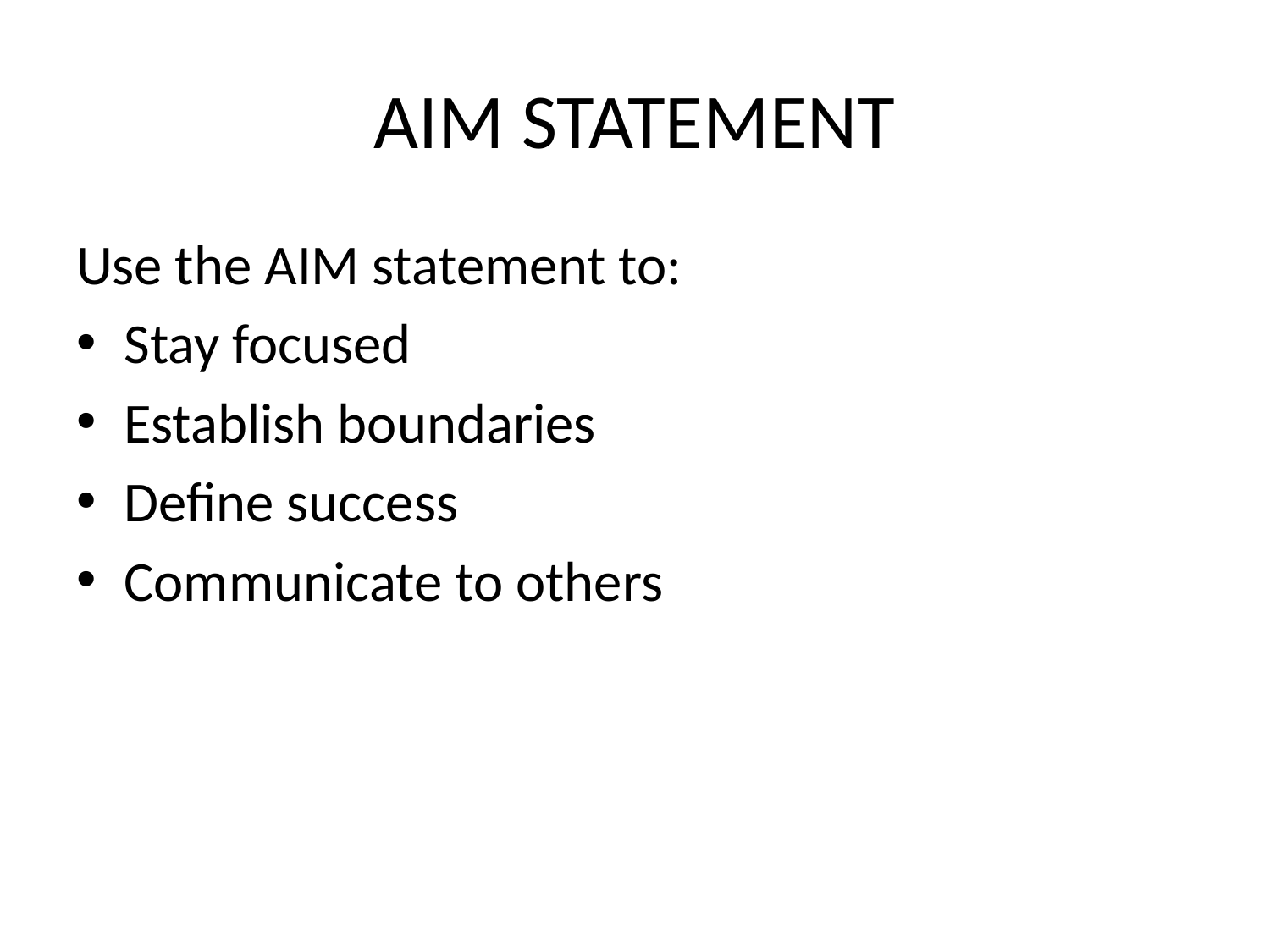

# AIM STATEMENT
Use the AIM statement to:
Stay focused
Establish boundaries
Define success
Communicate to others

## Slide 14
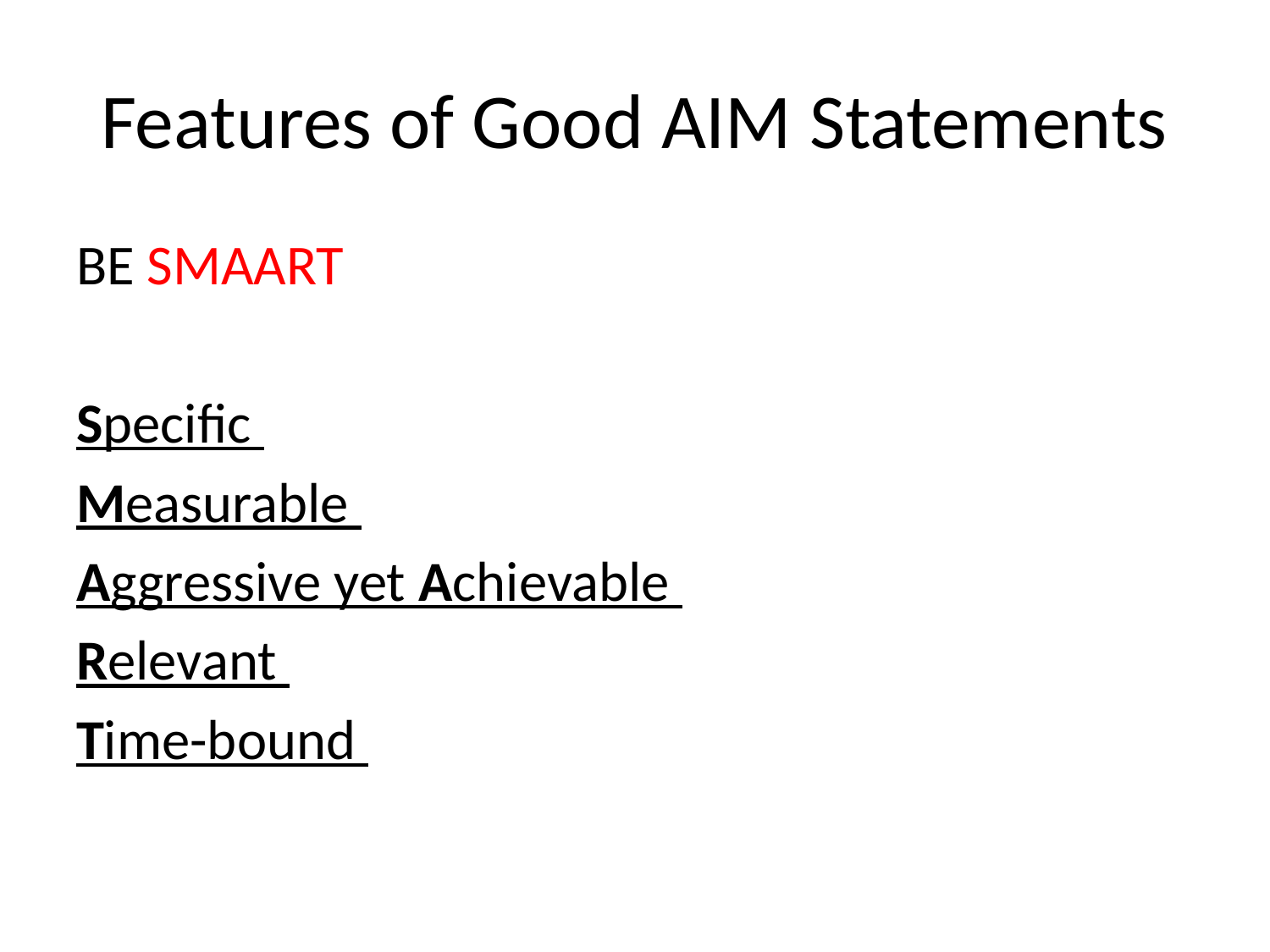

# Features of Good AIM Statements
BE SMAART
Specific
Measurable
Aggressive yet Achievable
Relevant
Time-bound

## Slide 15
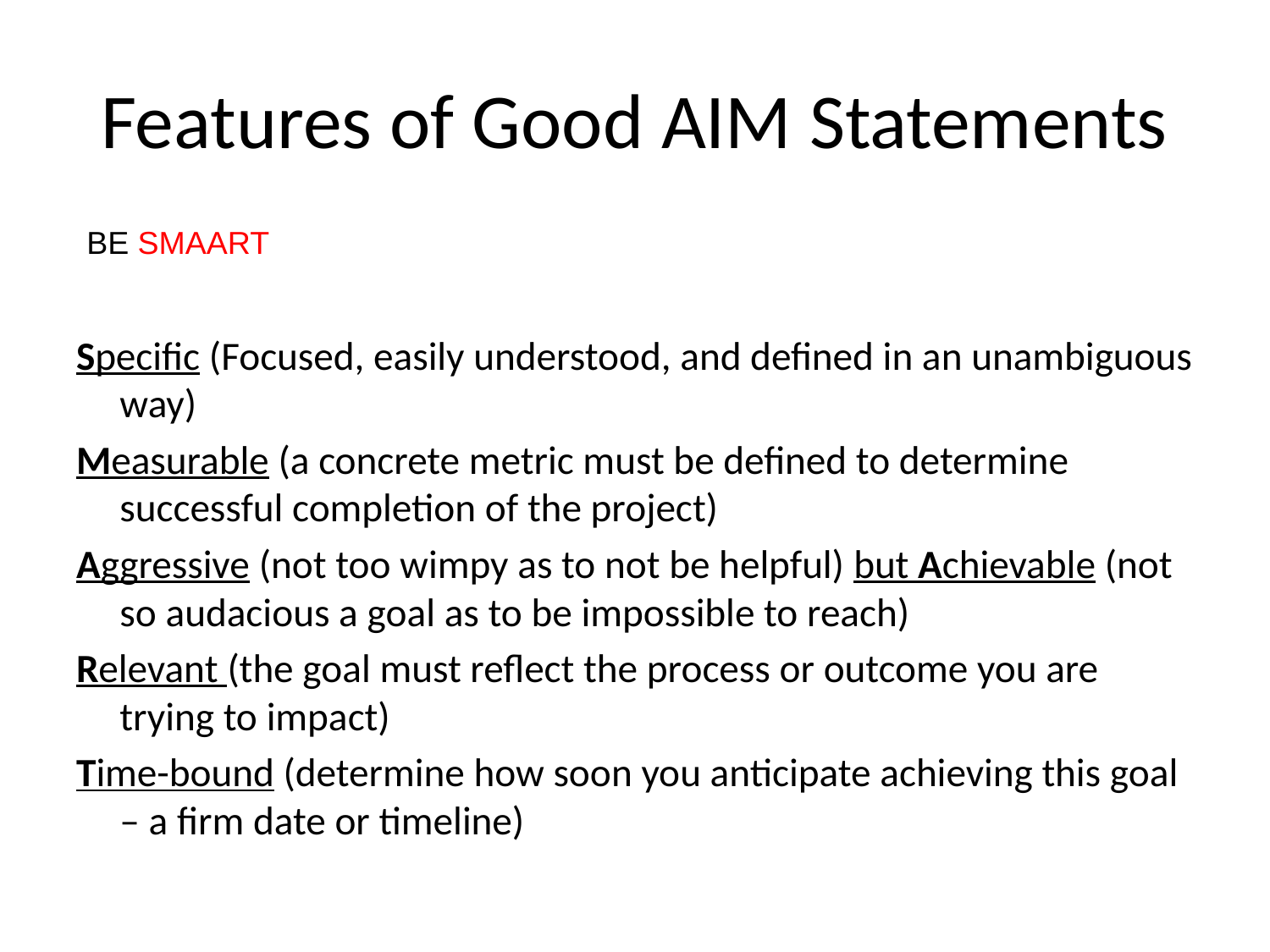

# Features of Good AIM Statements
BE SMAART
Specific (Focused, easily understood, and defined in an unambiguous way)
Measurable (a concrete metric must be defined to determine successful completion of the project)
Aggressive (not too wimpy as to not be helpful) but Achievable (not so audacious a goal as to be impossible to reach)
Relevant (the goal must reflect the process or outcome you are trying to impact)
Time-bound (determine how soon you anticipate achieving this goal – a firm date or timeline)

## Slide 16
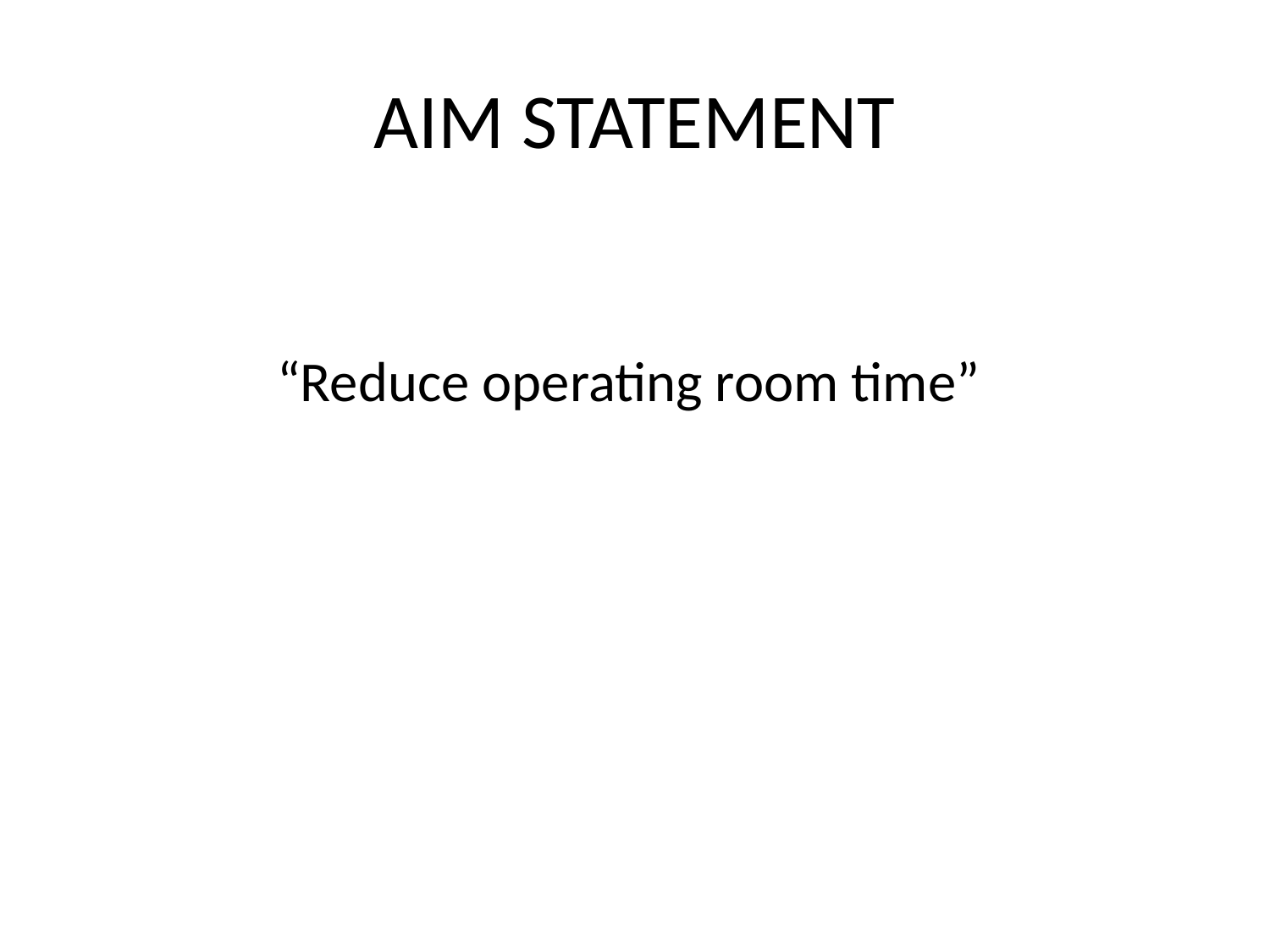

# AIM STATEMENT
“Reduce operating room time”

## Slide 17
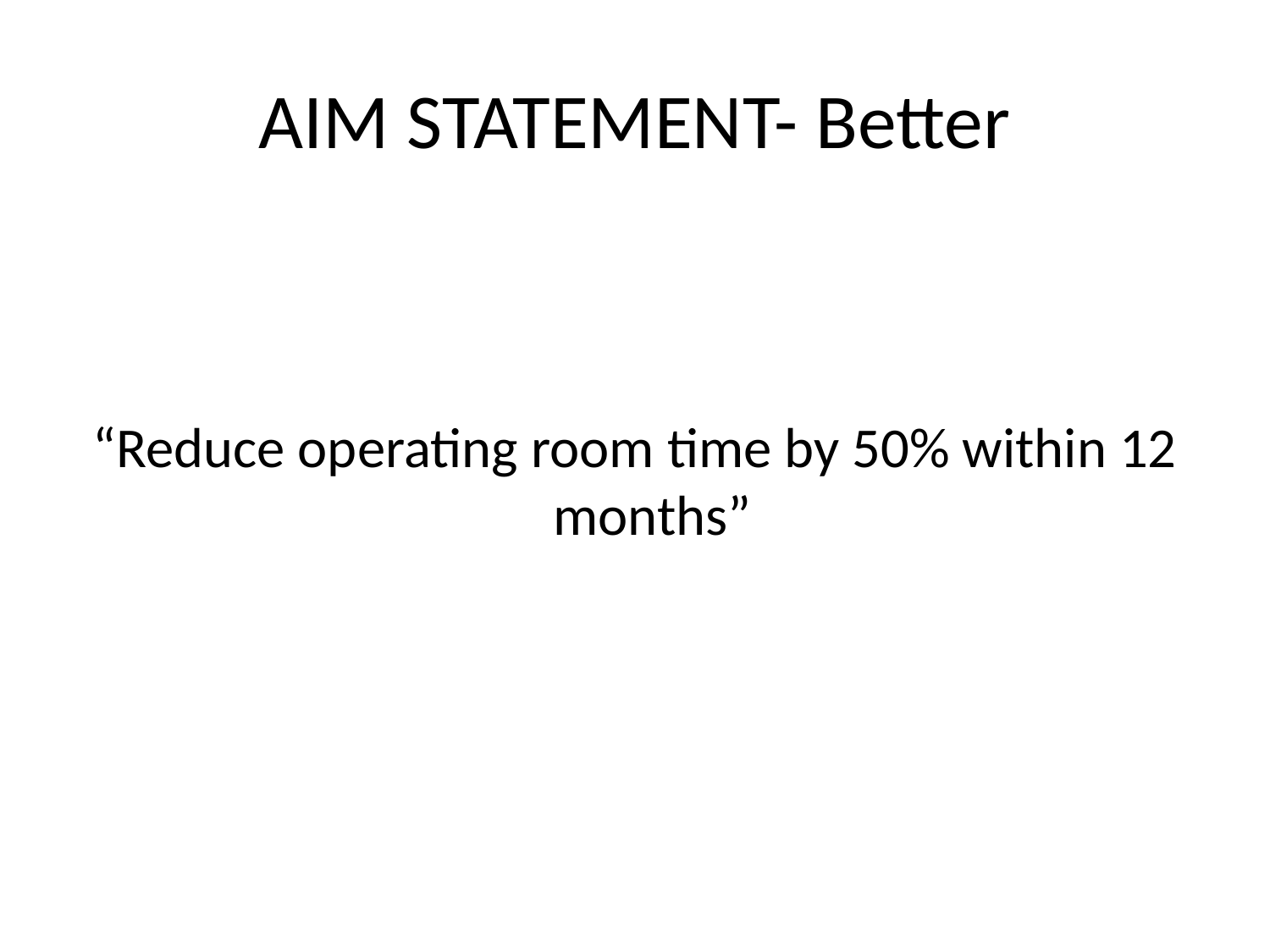

# AIM STATEMENT- Better
“Reduce operating room time by 50% within 12 months”

## Slide 18
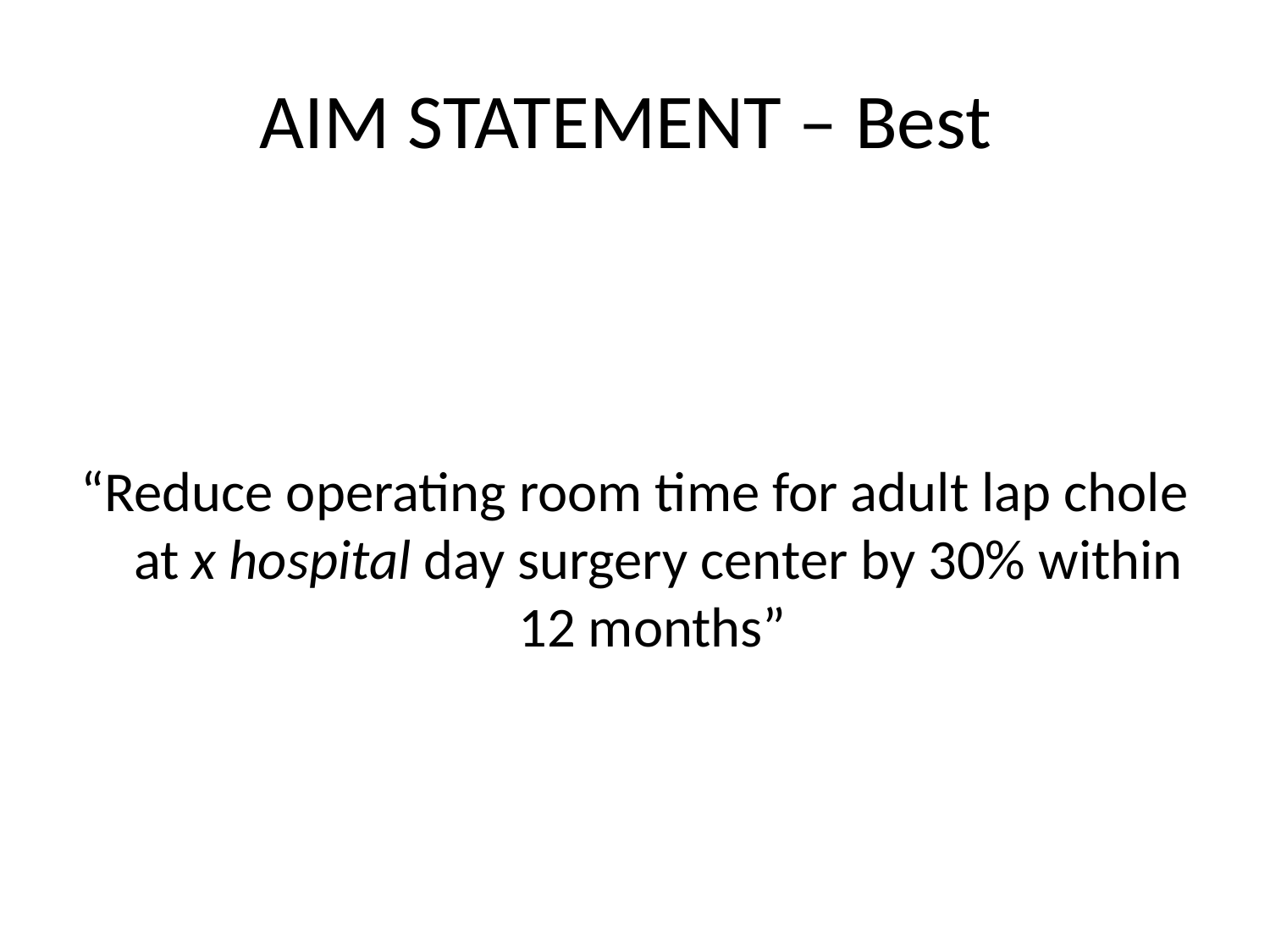

# AIM STATEMENT – Best
“Reduce operating room time for adult lap chole at x hospital day surgery center by 30% within 12 months”

## Slide 19
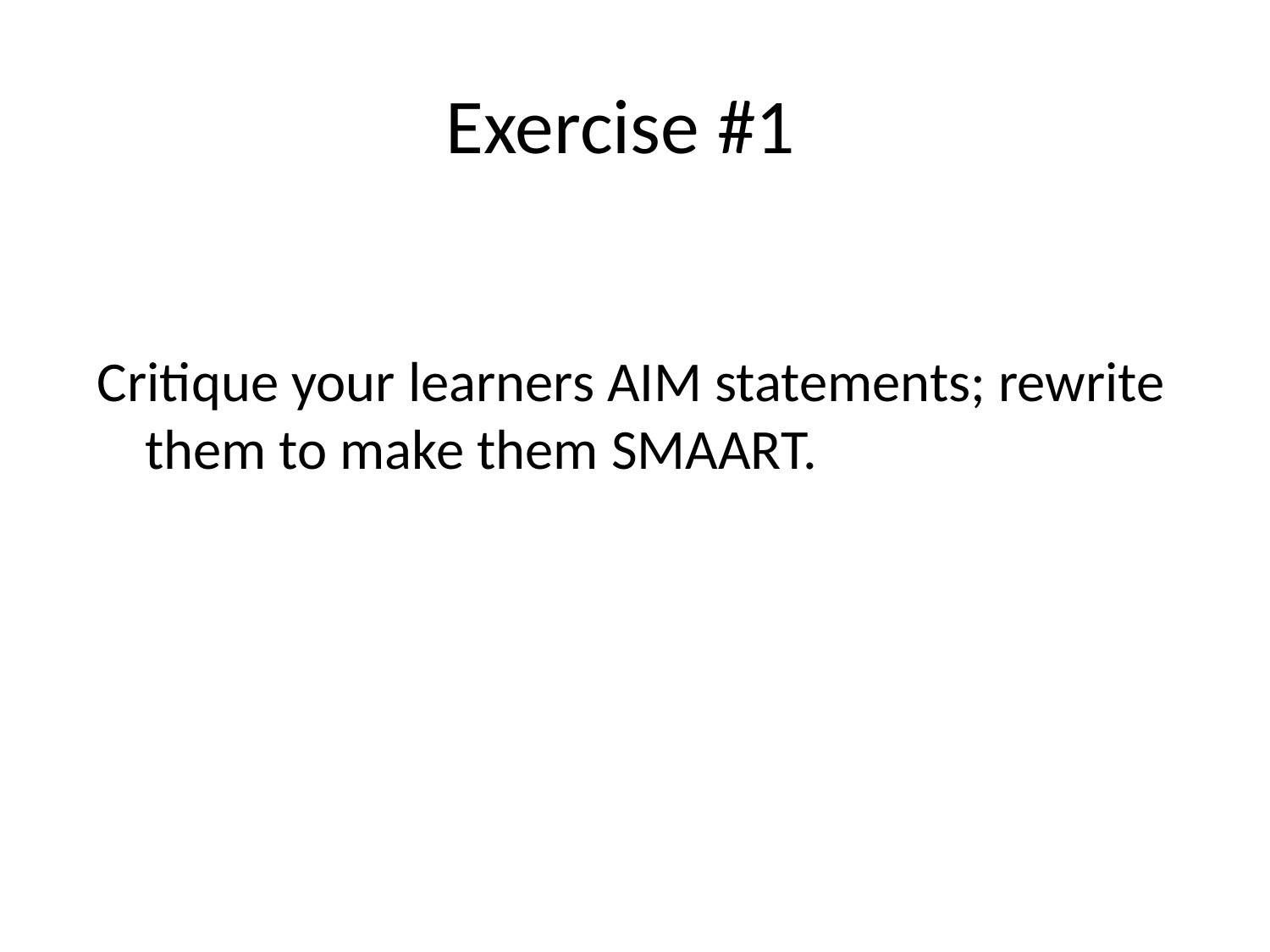

Exercise #1
Critique your learners AIM statements; rewrite them to make them SMAART.

## Slide 20
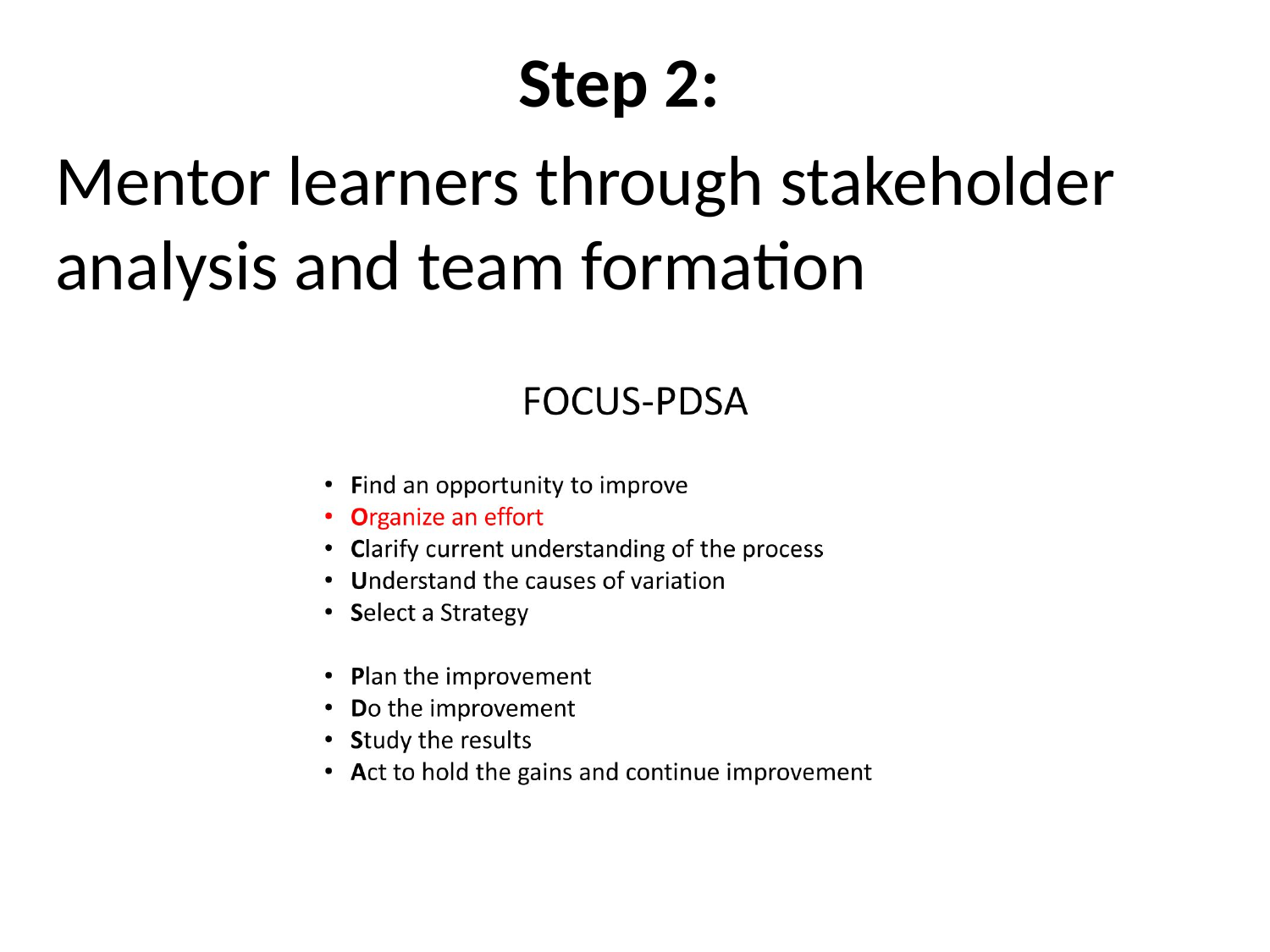

Step 2:
Mentor learners through stakeholder analysis and team formation

## Slide 21
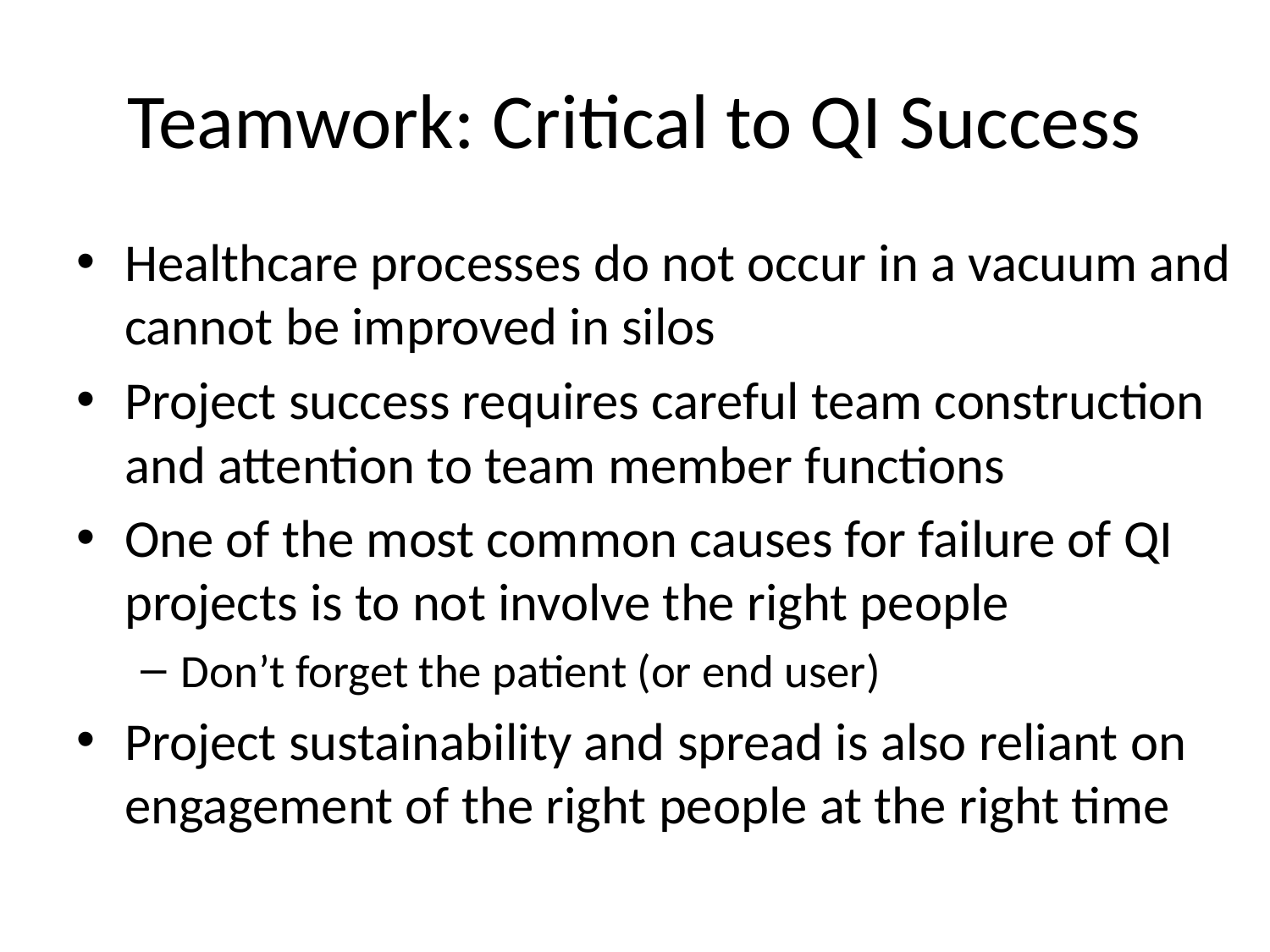

# Teamwork: Critical to QI Success
Healthcare processes do not occur in a vacuum and cannot be improved in silos
Project success requires careful team construction and attention to team member functions
One of the most common causes for failure of QI projects is to not involve the right people
Don’t forget the patient (or end user)
Project sustainability and spread is also reliant on engagement of the right people at the right time

## Slide 22
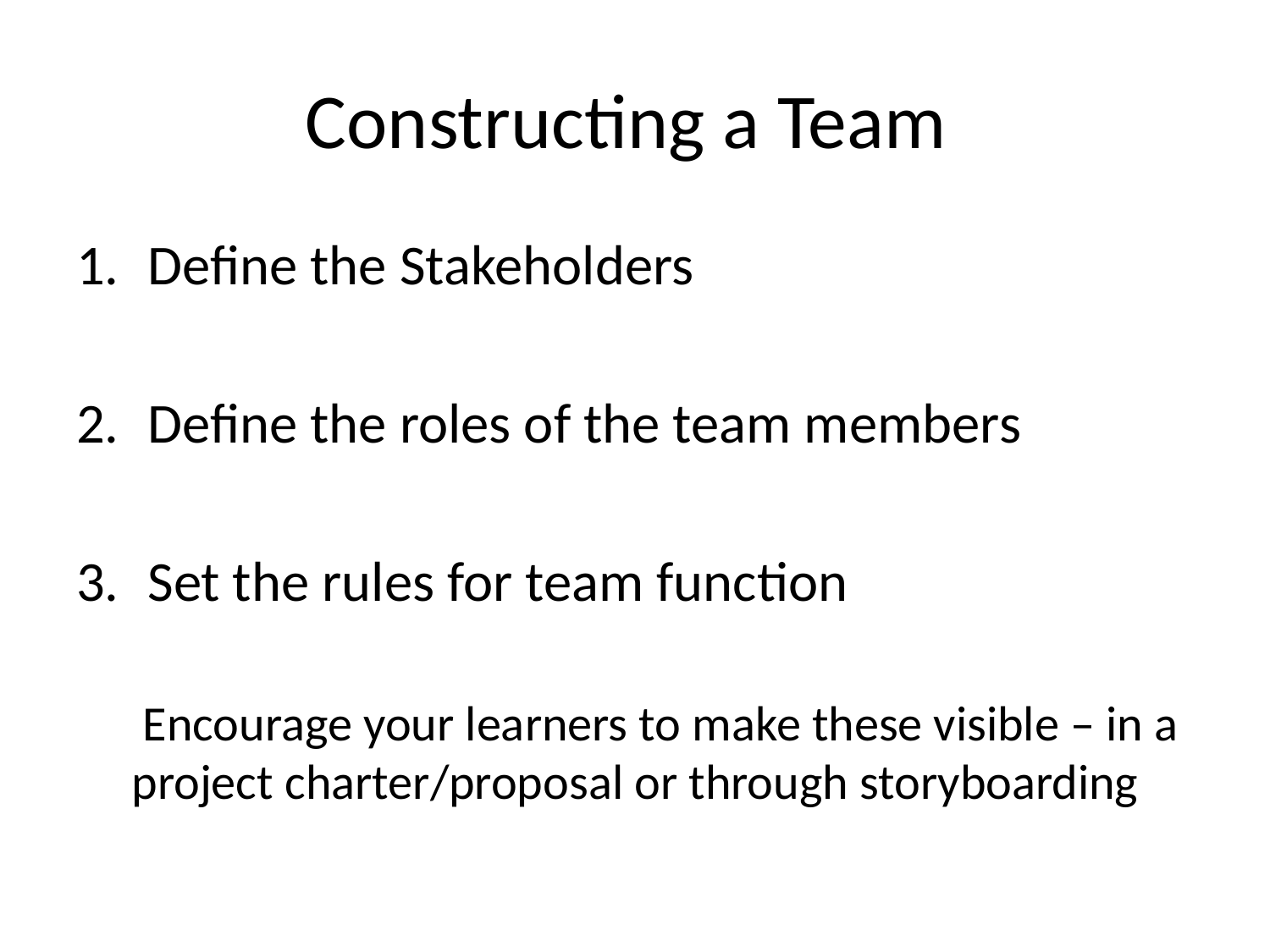

# Constructing a Team
Define the Stakeholders
Define the roles of the team members
Set the rules for team function
 Encourage your learners to make these visible – in a project charter/proposal or through storyboarding

## Slide 23
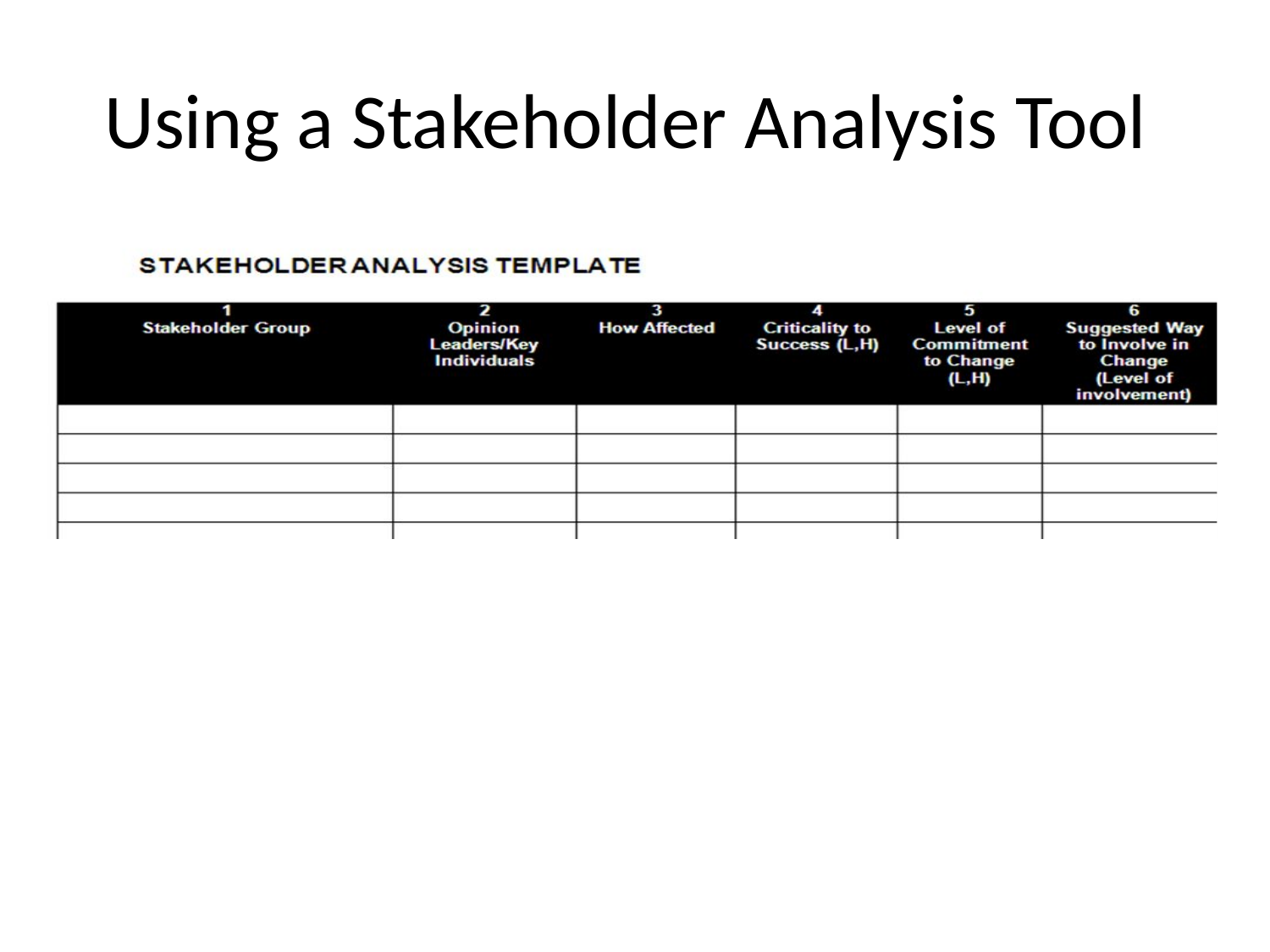

# Using a Stakeholder Analysis Tool

## Slide 24
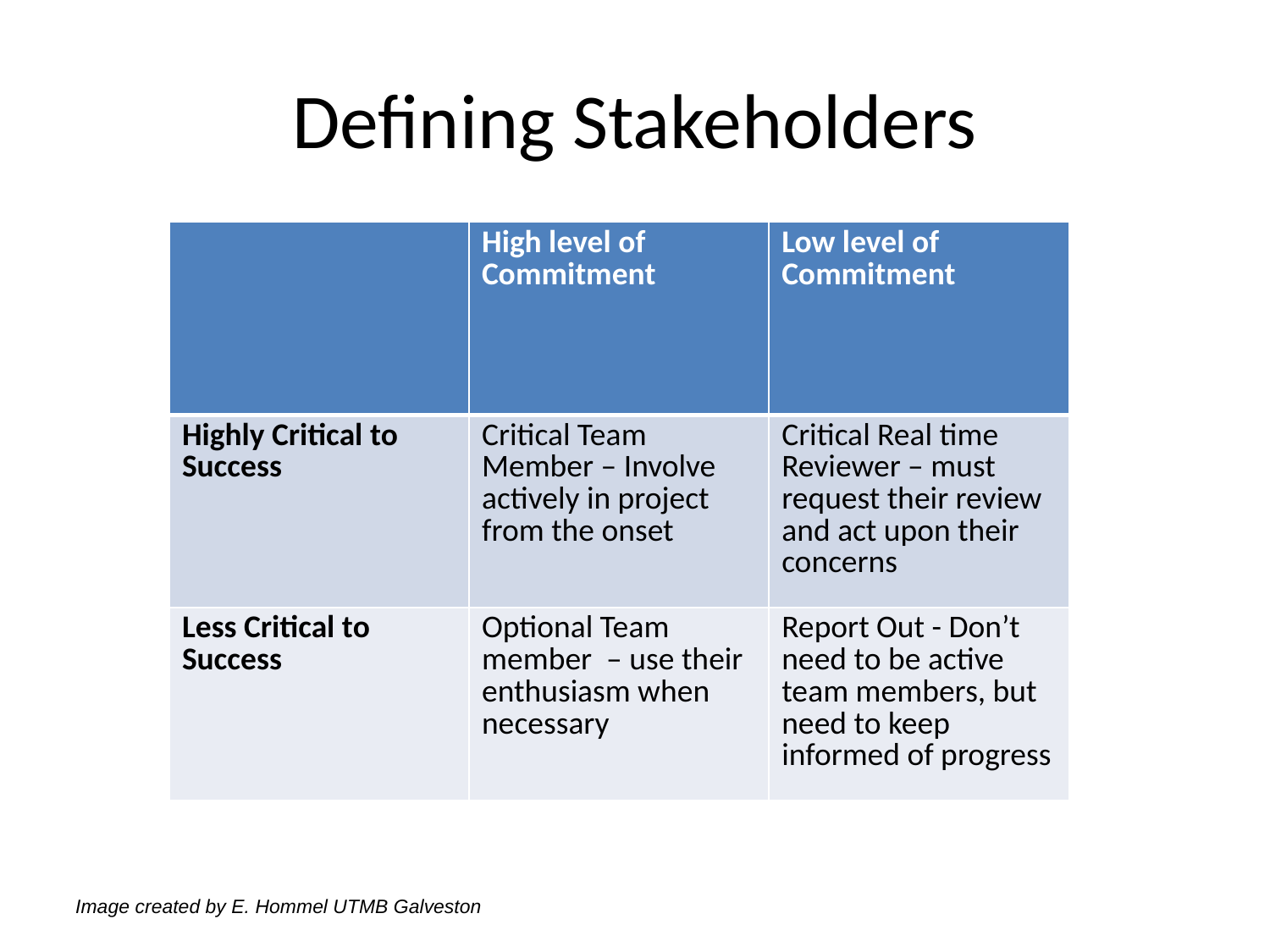

# Defining Stakeholders
| | High level of Commitment | Low level of Commitment |
| --- | --- | --- |
| Highly Critical to Success | Critical Team Member – Involve actively in project from the onset | Critical Real time Reviewer – must request their review and act upon their concerns |
| Less Critical to Success | Optional Team member – use their enthusiasm when necessary | Report Out - Don’t need to be active team members, but need to keep informed of progress |
Image created by E. Hommel UTMB Galveston

## Slide 25
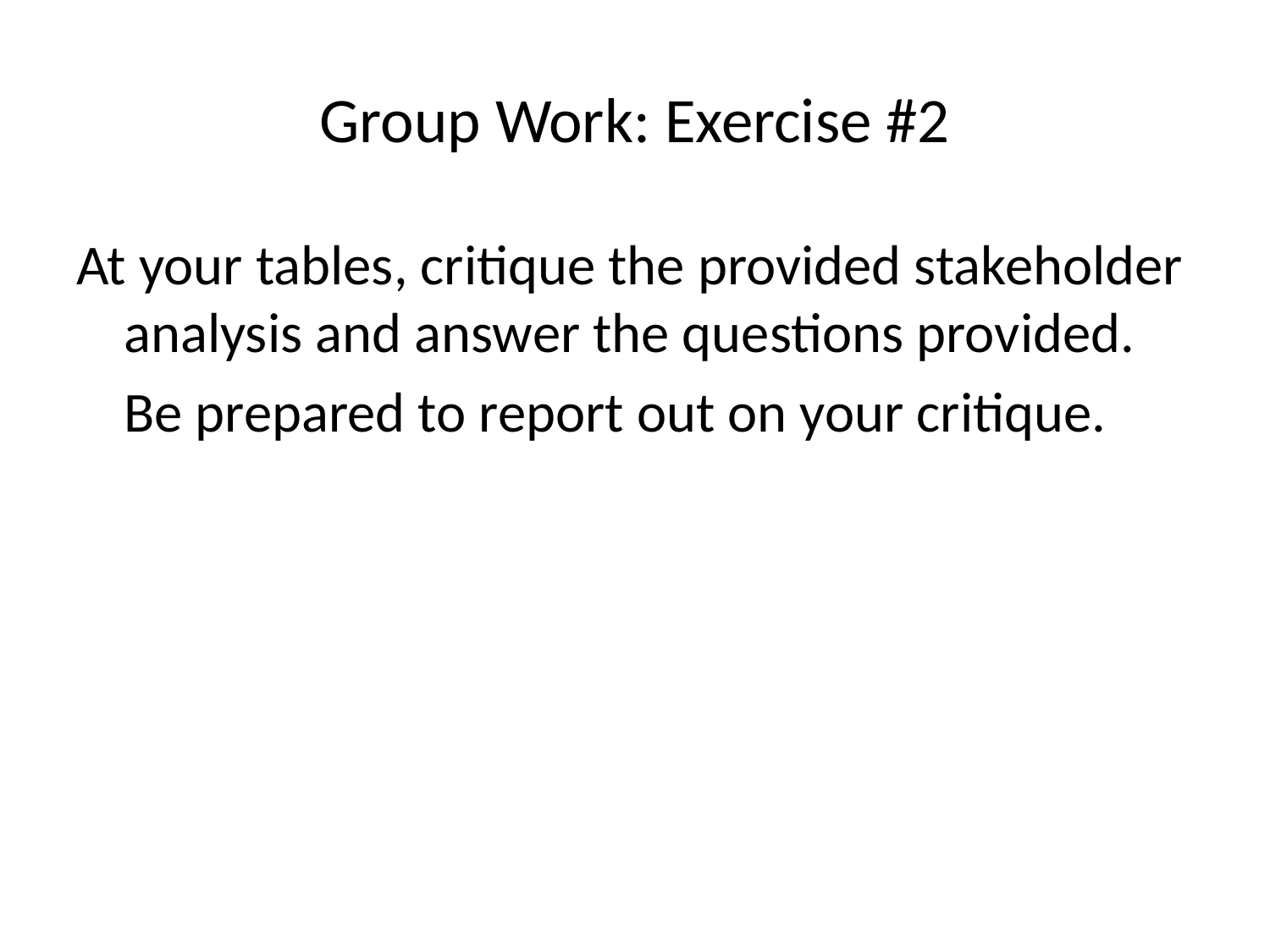

# Group Work: Exercise #2
At your tables, critique the provided stakeholder analysis and answer the questions provided.
	Be prepared to report out on your critique.

## Slide 26
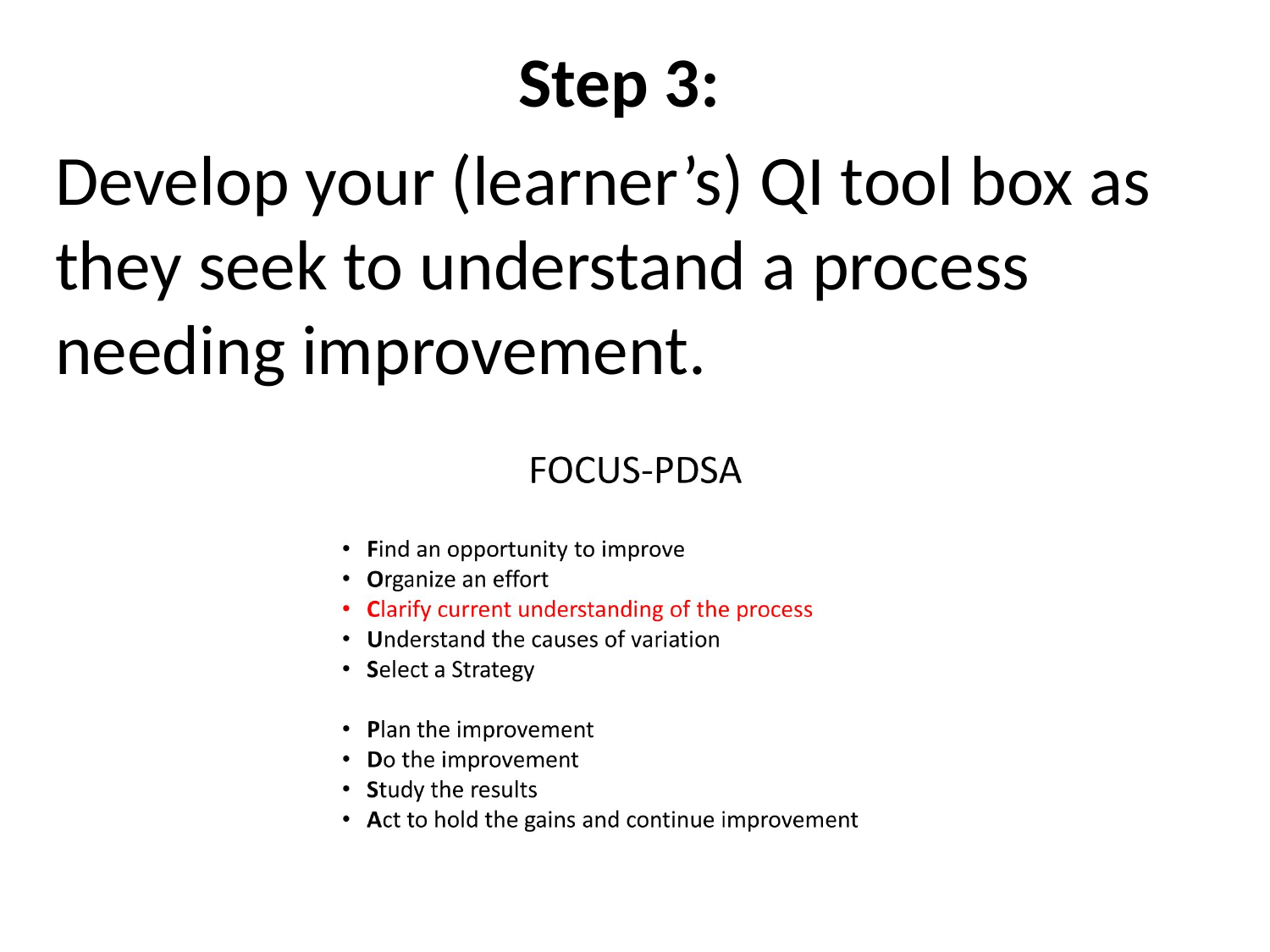

Step 3:
Develop your (learner’s) QI tool box as they seek to understand a process needing improvement.

## Slide 27
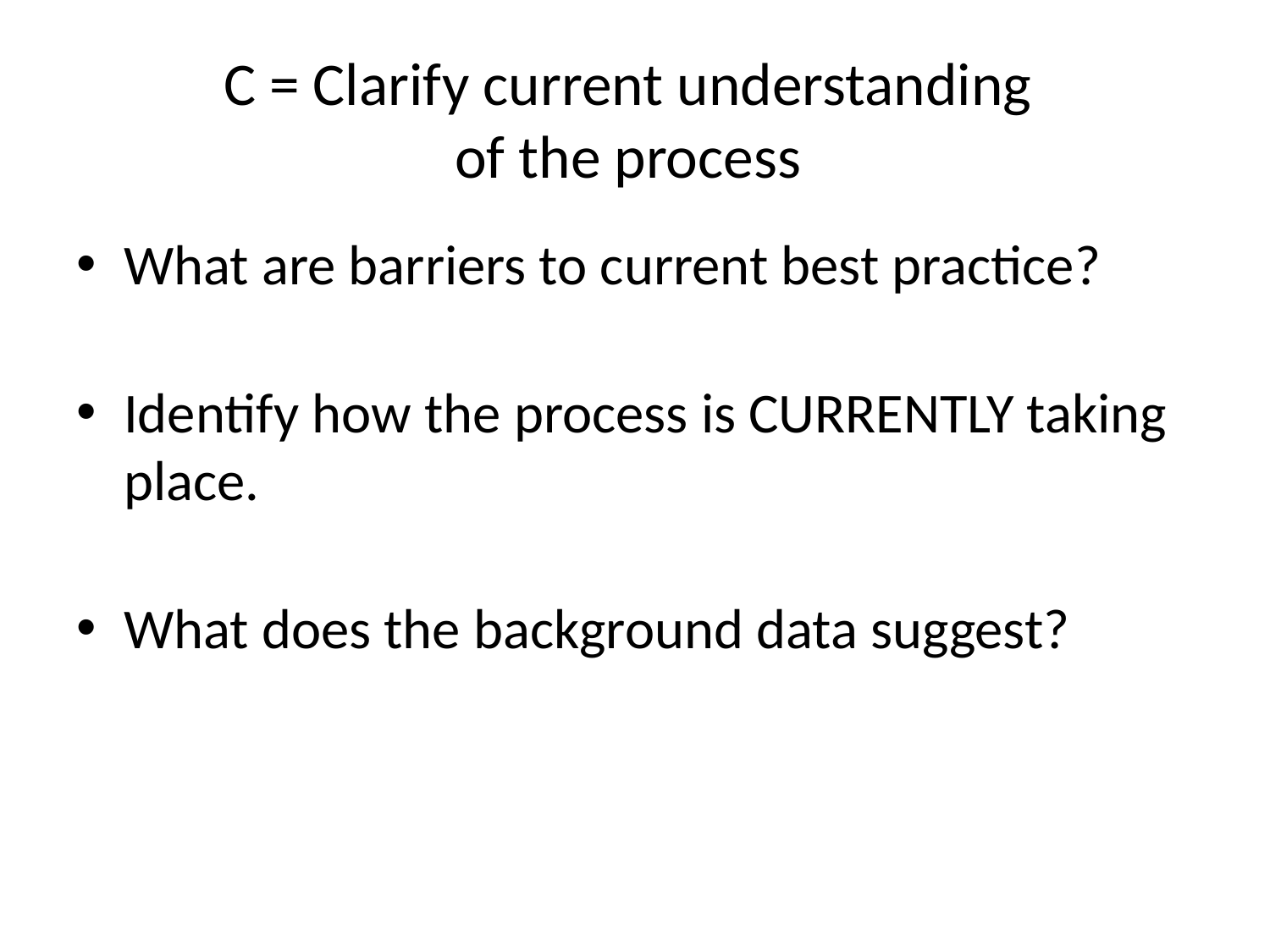

# C = Clarify current understanding of the process
What are barriers to current best practice?
Identify how the process is CURRENTLY taking place.
What does the background data suggest?

## Slide 28
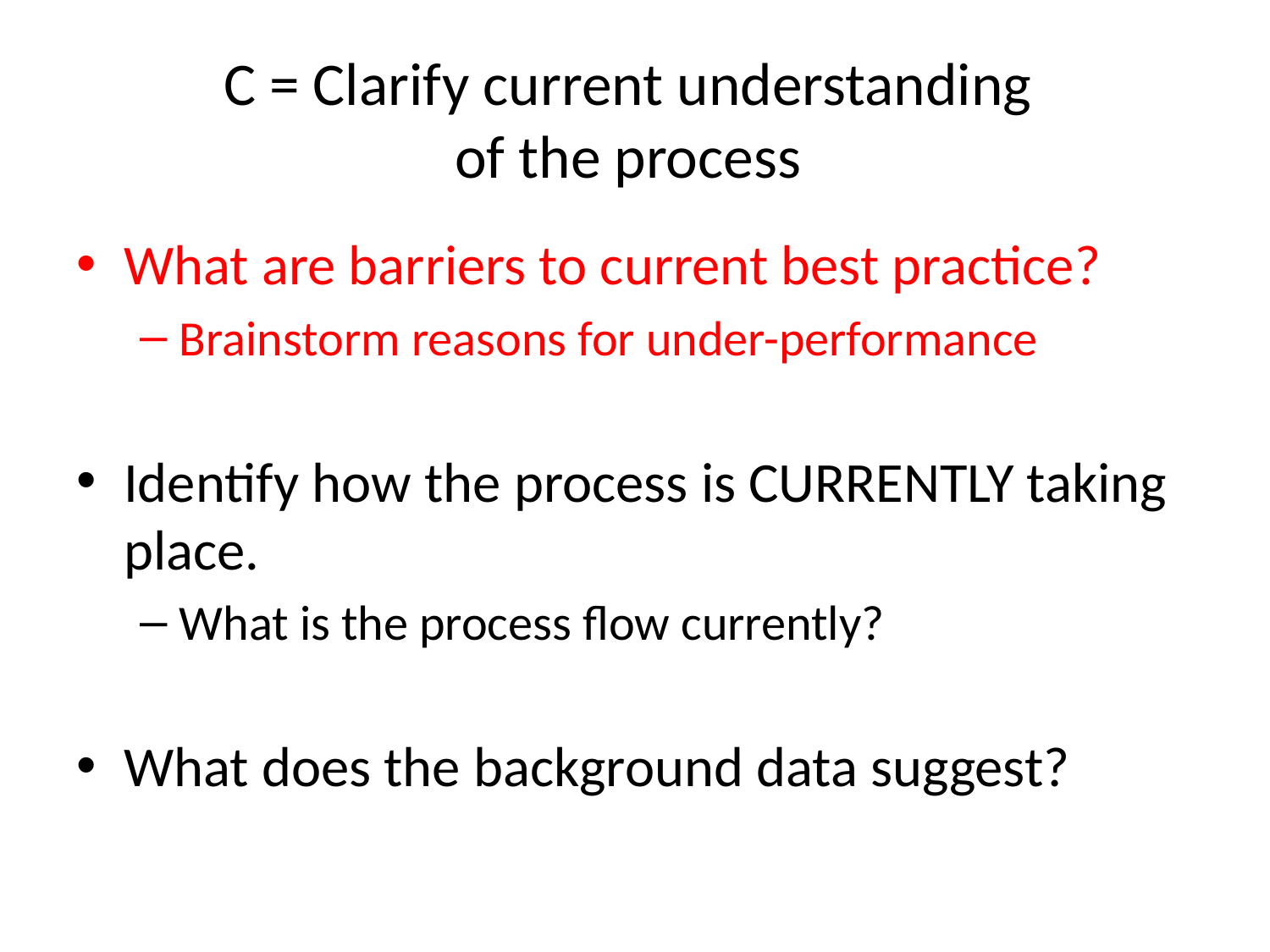

# C = Clarify current understanding of the process
What are barriers to current best practice?
Brainstorm reasons for under-performance
Identify how the process is CURRENTLY taking place.
What is the process flow currently?
What does the background data suggest?

## Slide 29
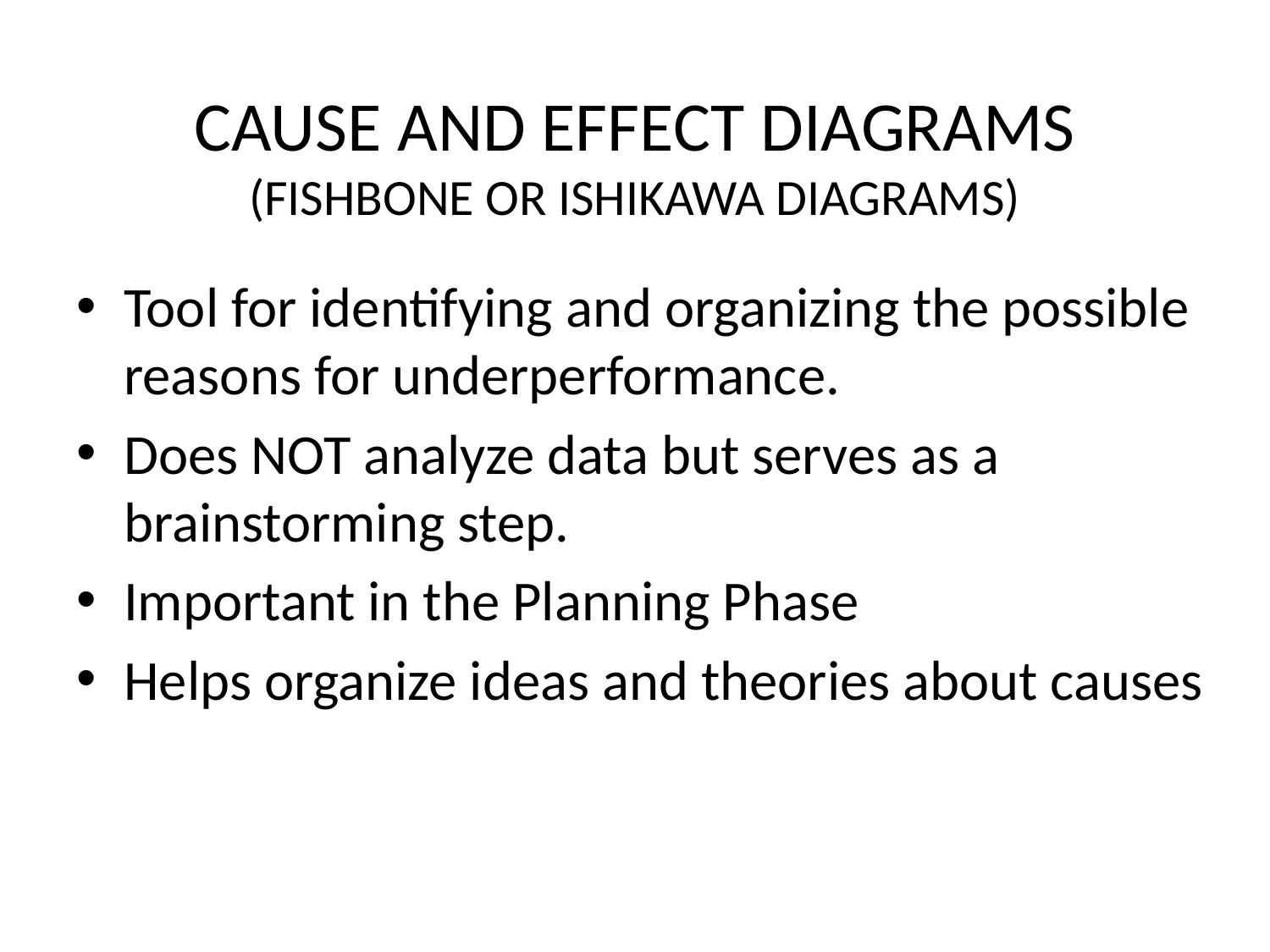

# CAUSE AND EFFECT DIAGRAMS (FISHBONE OR ISHIKAWA DIAGRAMS)
Tool for identifying and organizing the possible reasons for underperformance.
Does NOT analyze data but serves as a brainstorming step.
Important in the Planning Phase
Helps organize ideas and theories about causes

## Slide 30
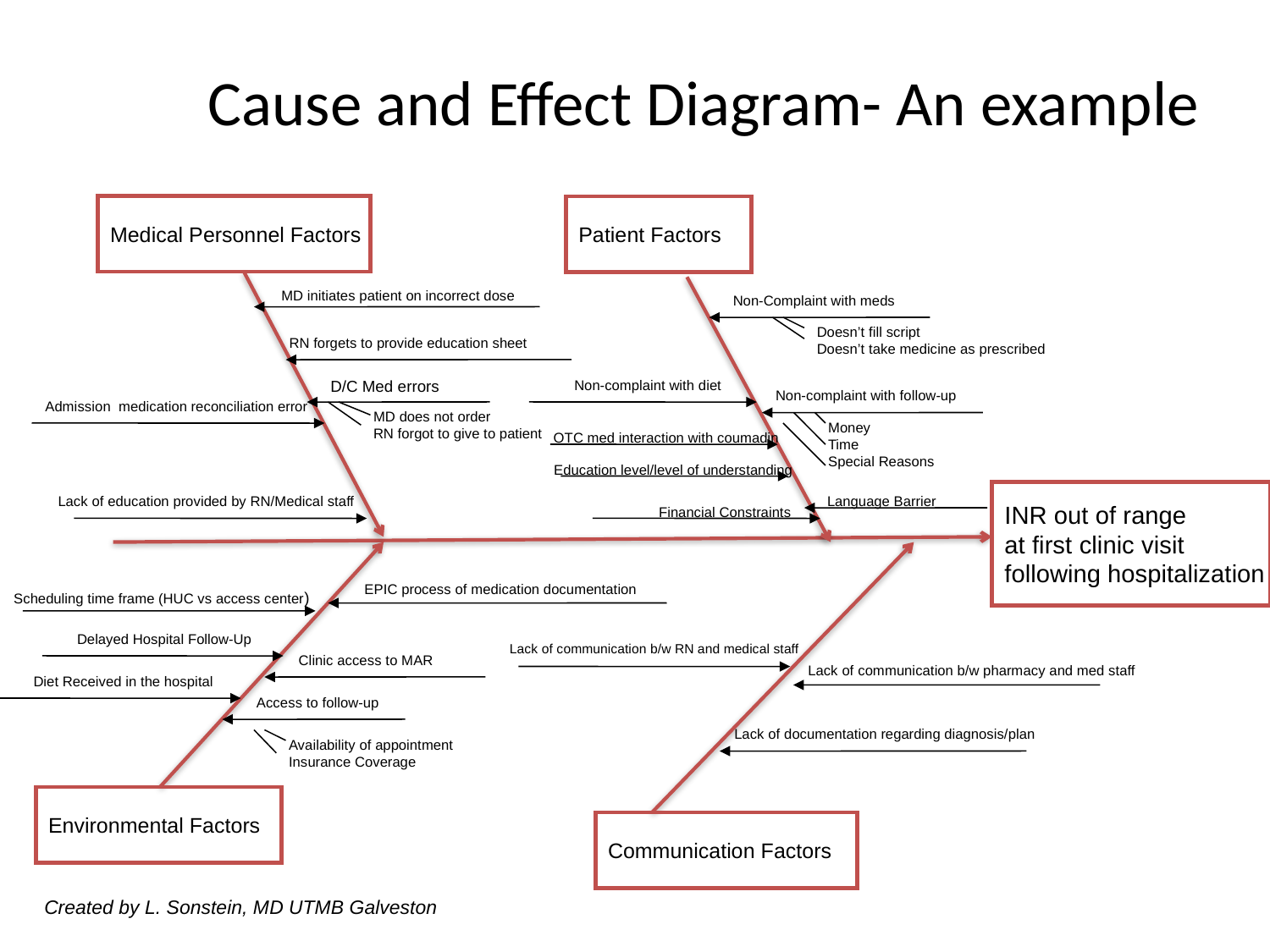

Cause and Effect Diagram- An example
Medical Personnel Factors
Patient Factors
MD initiates patient on incorrect dose
Non-Complaint with meds
Doesn’t fill script
Doesn’t take medicine as prescribed
RN forgets to provide education sheet
D/C Med errors
Non-complaint with diet
Non-complaint with follow-up
Admission medication reconciliation error
MD does not order
RN forgot to give to patient
Money
Time
Special Reasons
OTC med interaction with coumadin
Education level/level of understanding
INR out of range
at first clinic visit
following hospitalization
Lack of education provided by RN/Medical staff
Language Barrier
Financial Constraints
EPIC process of medication documentation
Scheduling time frame (HUC vs access center)
Delayed Hospital Follow-Up
Lack of communication b/w RN and medical staff
Clinic access to MAR
Lack of communication b/w pharmacy and med staff
Diet Received in the hospital
Access to follow-up
Lack of documentation regarding diagnosis/plan
Availability of appointment
Insurance Coverage
Environmental Factors
Communication Factors
Created by L. Sonstein, MD UTMB Galveston

## Slide 31
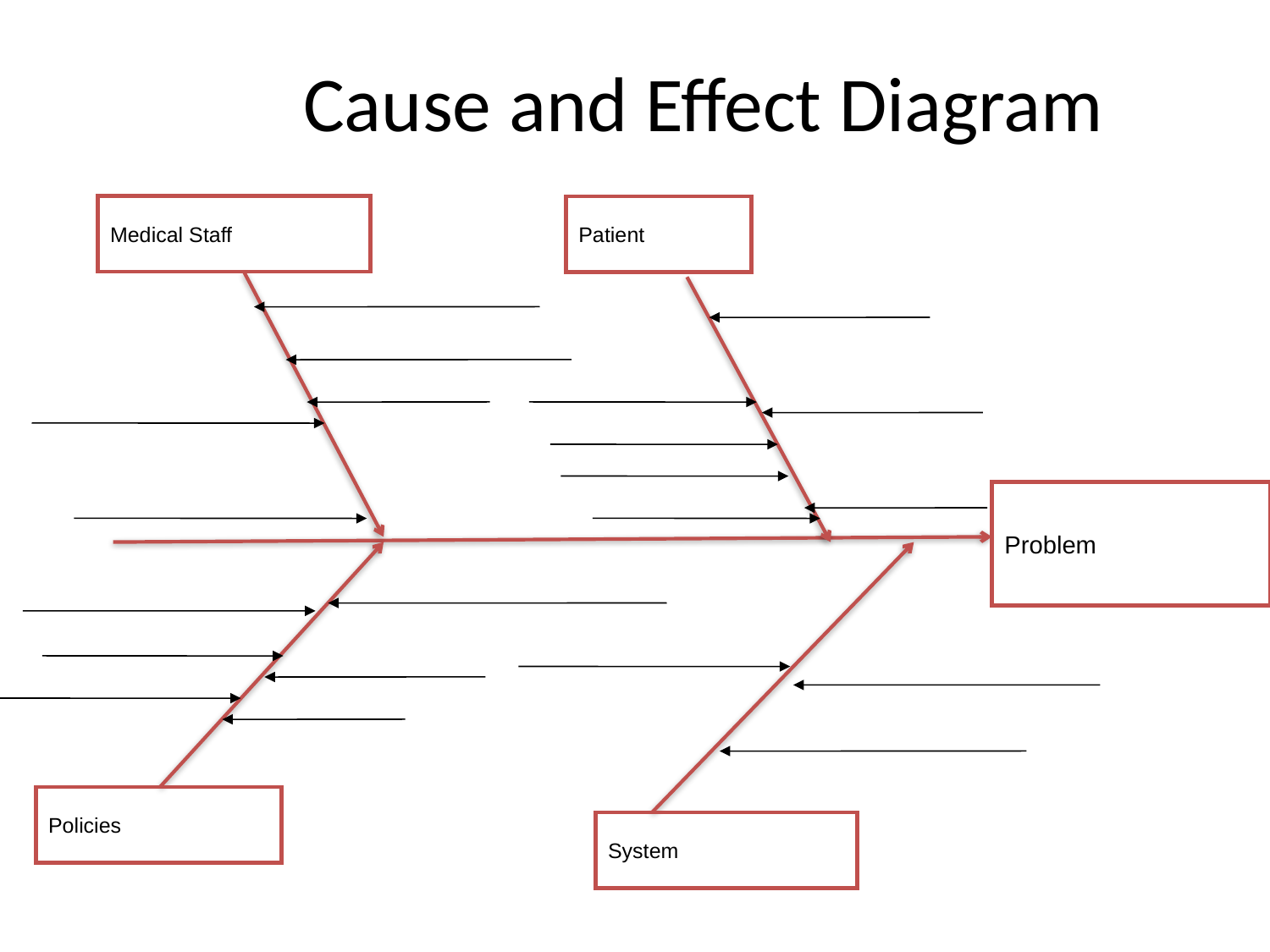

Cause and Effect Diagram
Medical Staff
Patient
Problem
Policies
System

## Slide 32
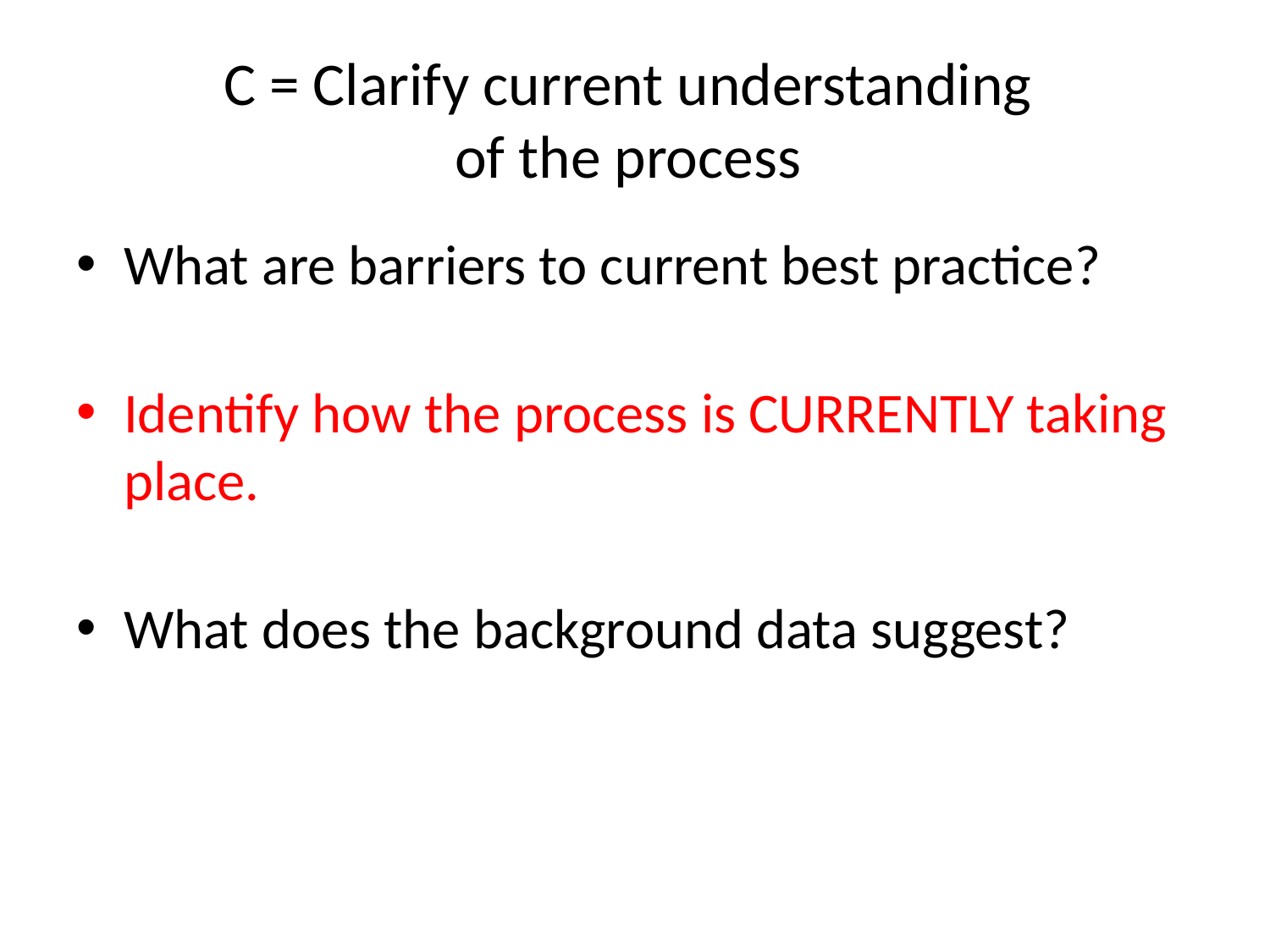

# C = Clarify current understanding of the process
What are barriers to current best practice?
Identify how the process is CURRENTLY taking place.
What does the background data suggest?

## Slide 33
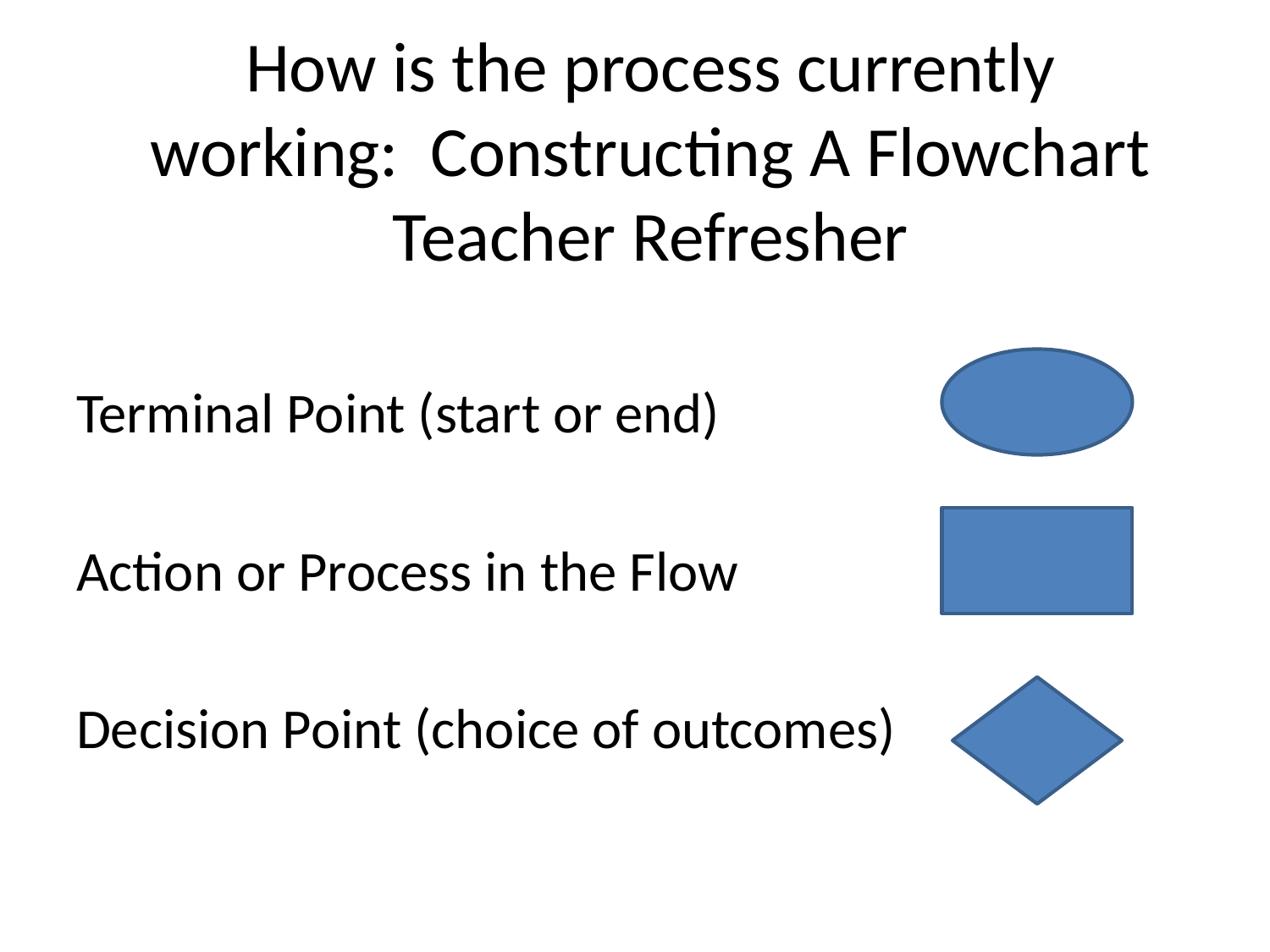

# How is the process currently working: Constructing A Flowchart Teacher Refresher
Terminal Point (start or end)
Action or Process in the Flow
Decision Point (choice of outcomes)

## Slide 34
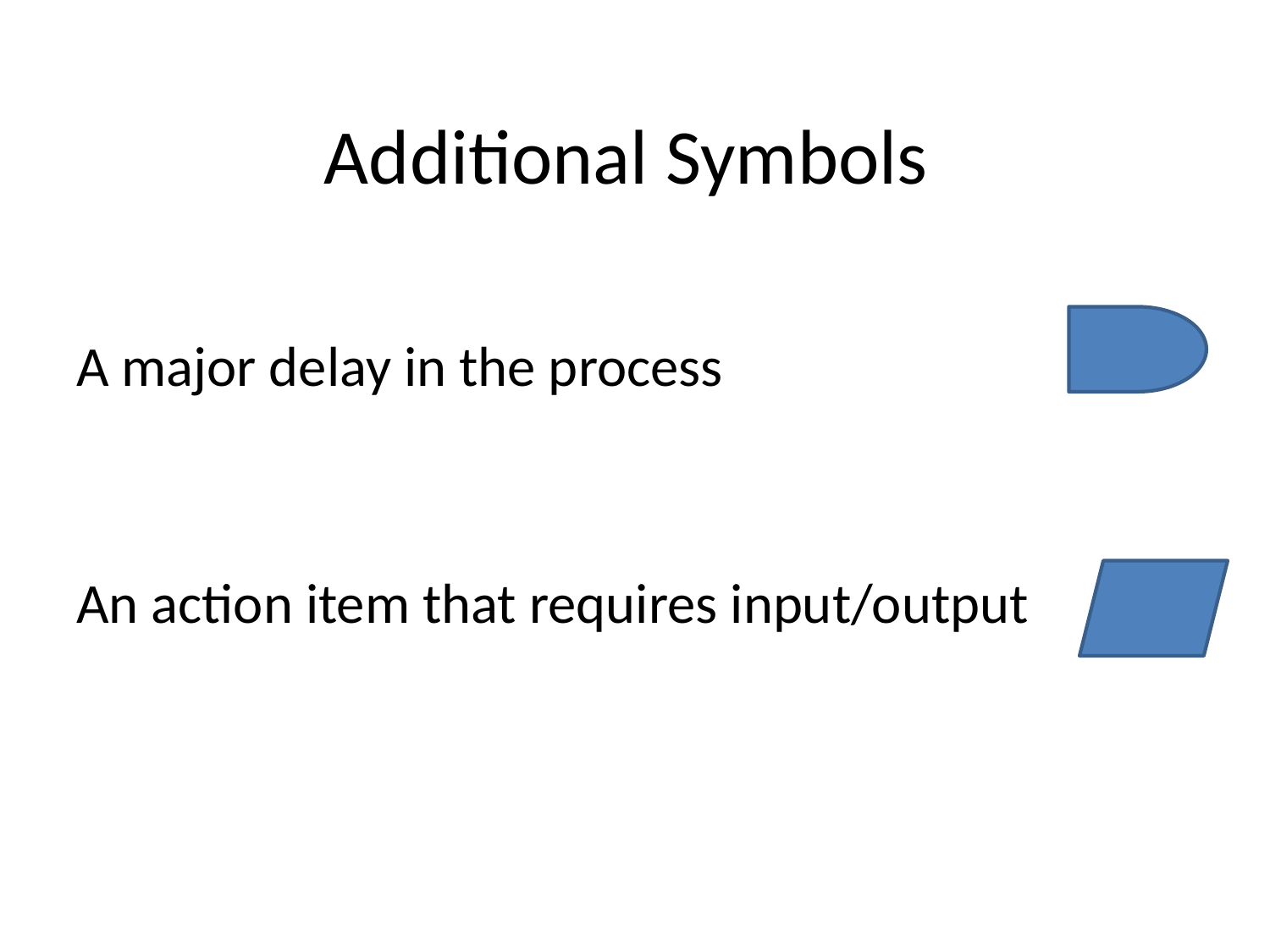

# Additional Symbols
A major delay in the process
An action item that requires input/output

## Slide 35
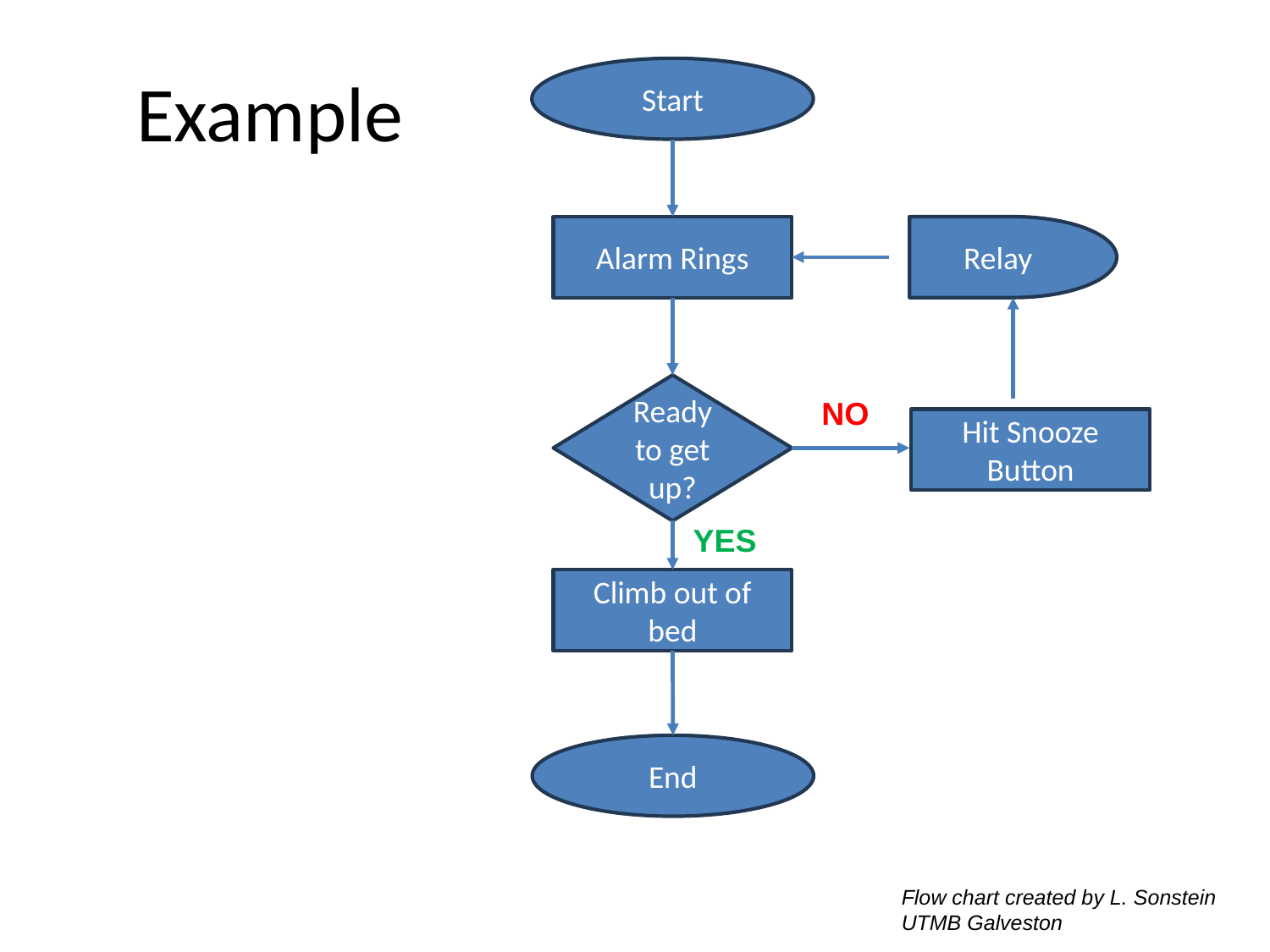

# Example
Start
Alarm Rings
Relay
Ready to get up?
NO
Hit Snooze Button
YES
Climb out of bed
End
Flow chart created by L. Sonstein UTMB Galveston

## Slide 36
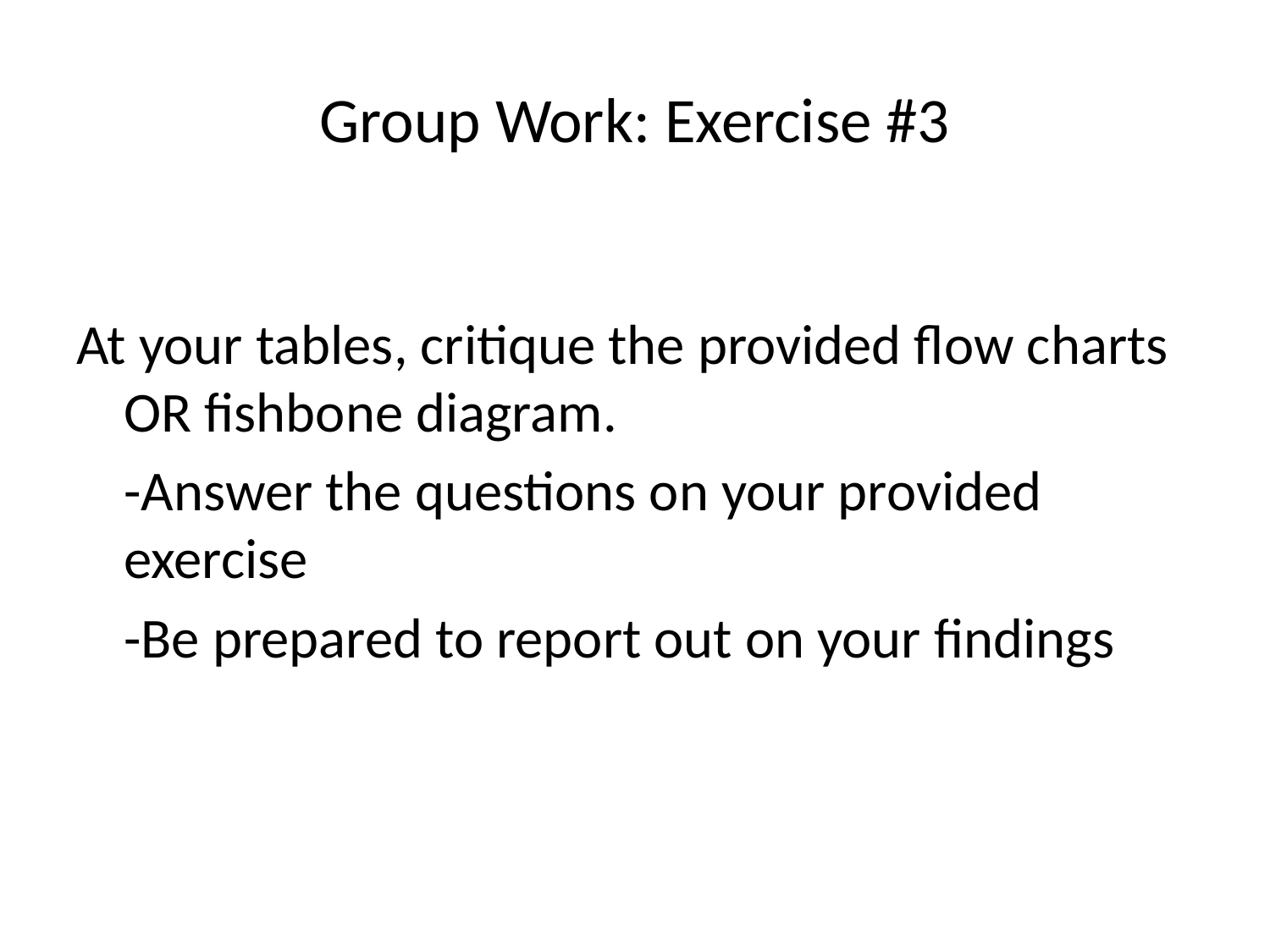

# Group Work: Exercise #3
At your tables, critique the provided flow charts OR fishbone diagram.
	-Answer the questions on your provided exercise
	-Be prepared to report out on your findings

## Slide 37
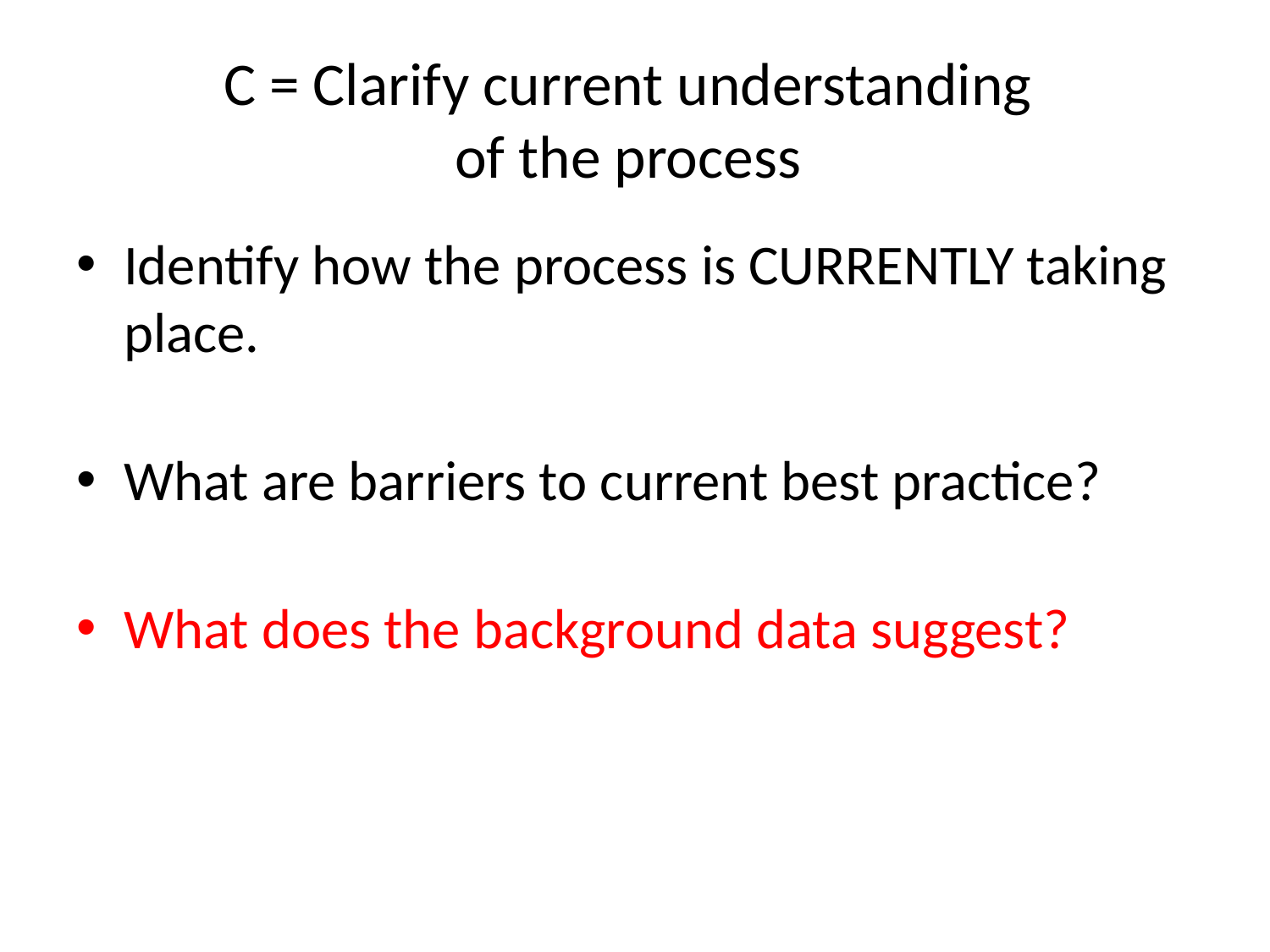

# C = Clarify current understanding of the process
Identify how the process is CURRENTLY taking place.
What are barriers to current best practice?
What does the background data suggest?

## Slide 38
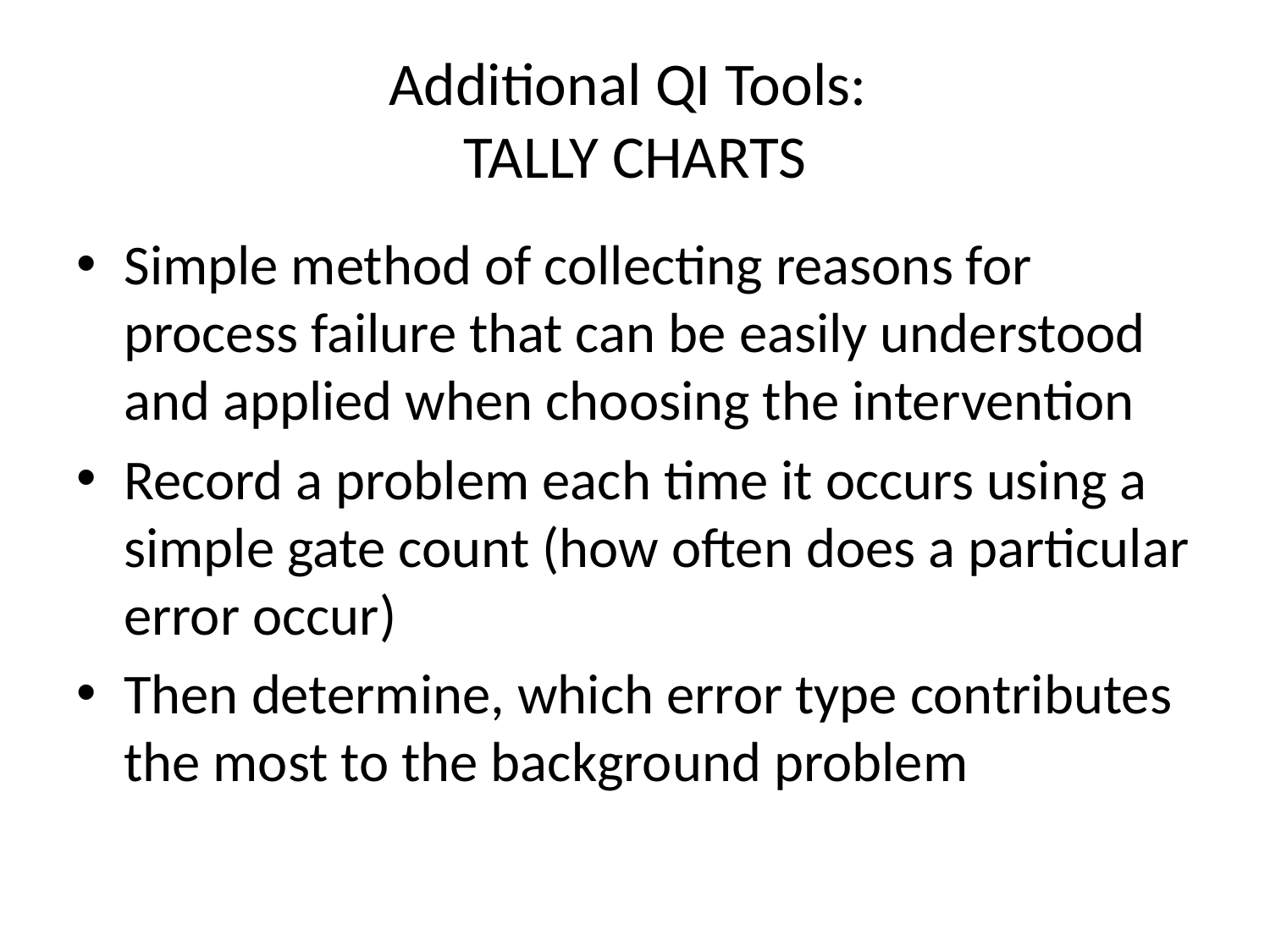

# Additional QI Tools: TALLY CHARTS
Simple method of collecting reasons for process failure that can be easily understood and applied when choosing the intervention
Record a problem each time it occurs using a simple gate count (how often does a particular error occur)
Then determine, which error type contributes the most to the background problem

## Slide 39
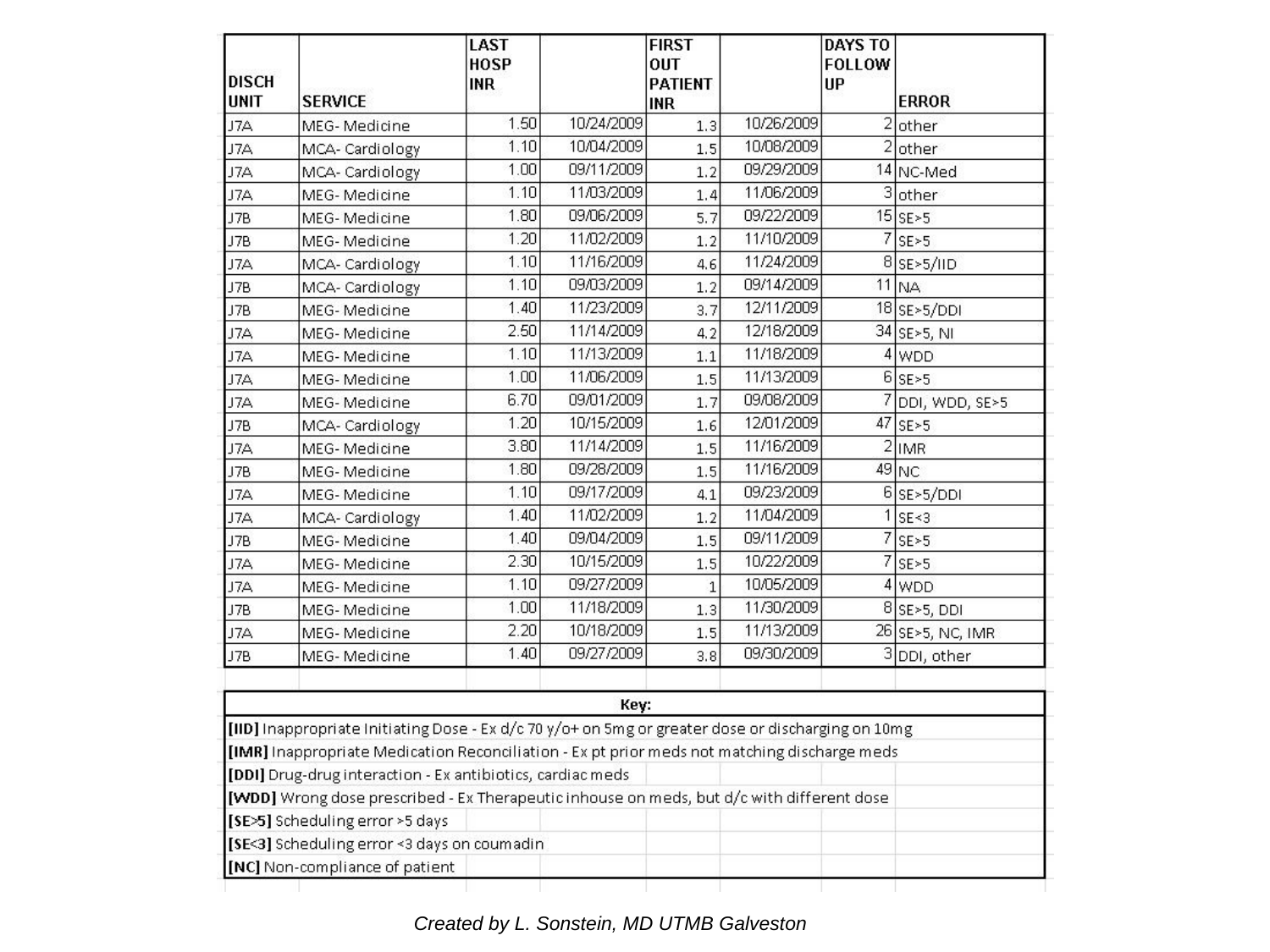

Created by L. Sonstein, MD UTMB Galveston

## Slide 40
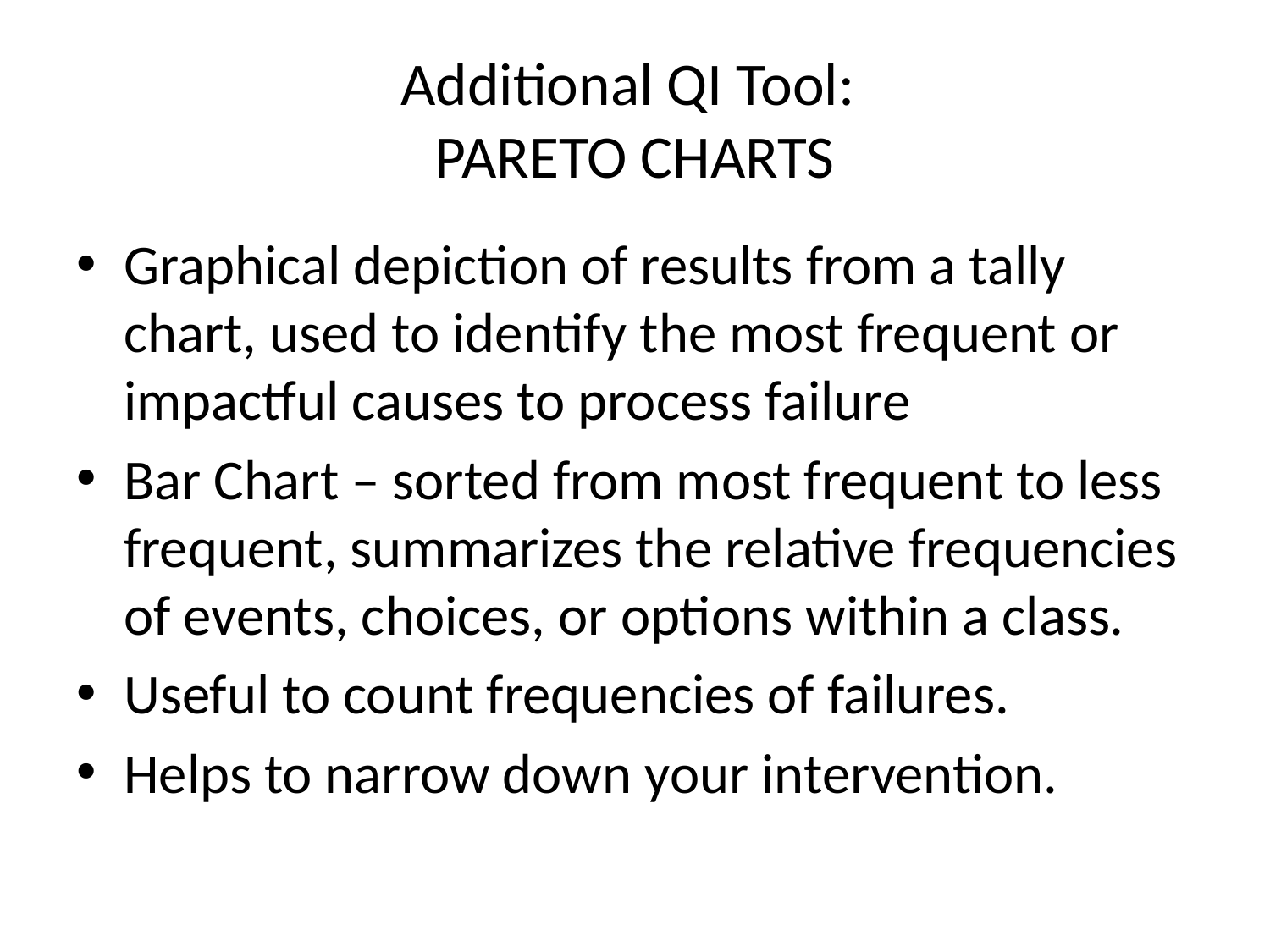

# Additional QI Tool: PARETO CHARTS
Graphical depiction of results from a tally chart, used to identify the most frequent or impactful causes to process failure
Bar Chart – sorted from most frequent to less frequent, summarizes the relative frequencies of events, choices, or options within a class.
Useful to count frequencies of failures.
Helps to narrow down your intervention.

## Slide 41
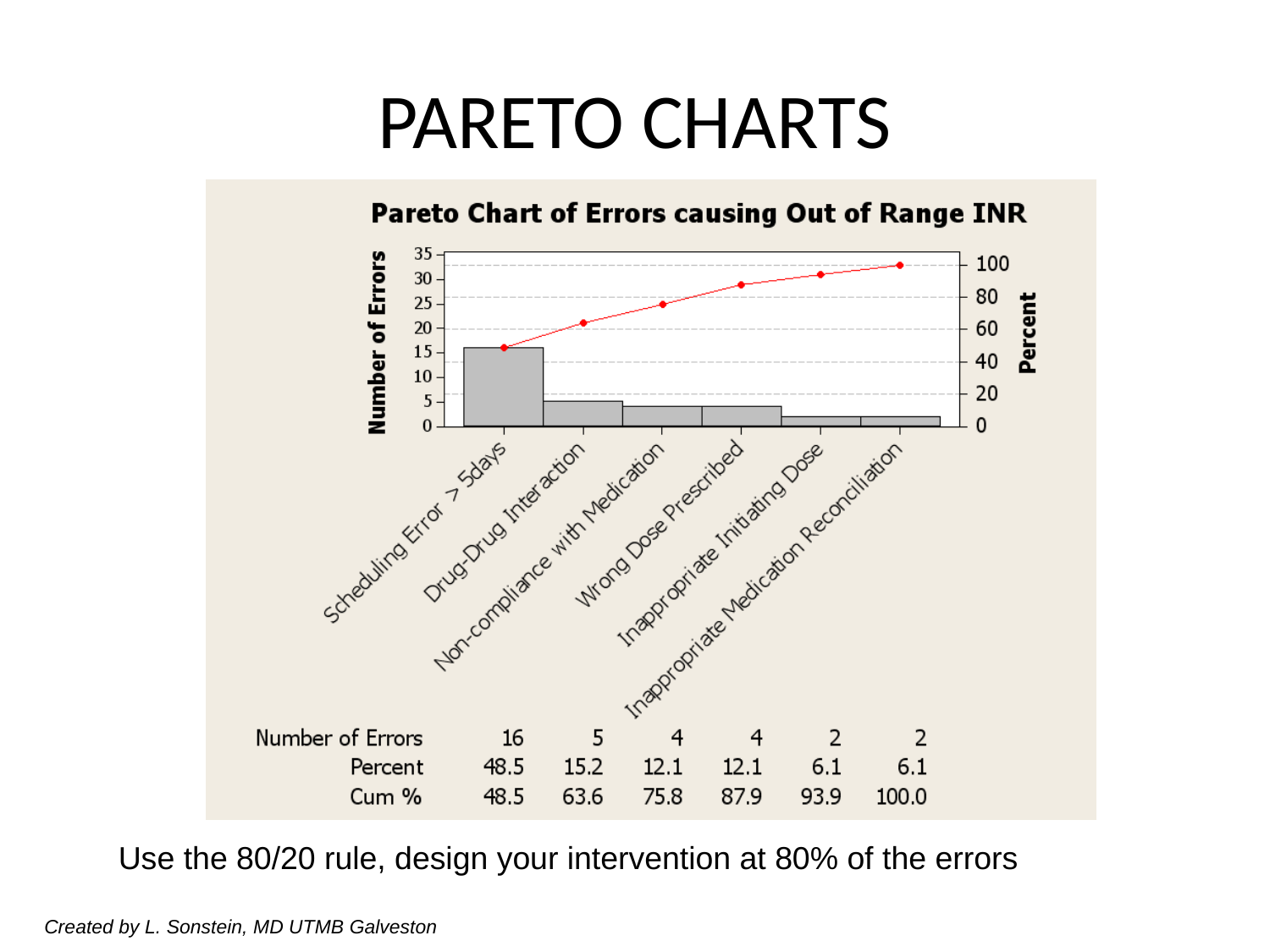

# PARETO CHARTS
Use the 80/20 rule, design your intervention at 80% of the errors
Created by L. Sonstein, MD UTMB Galveston

## Slide 42
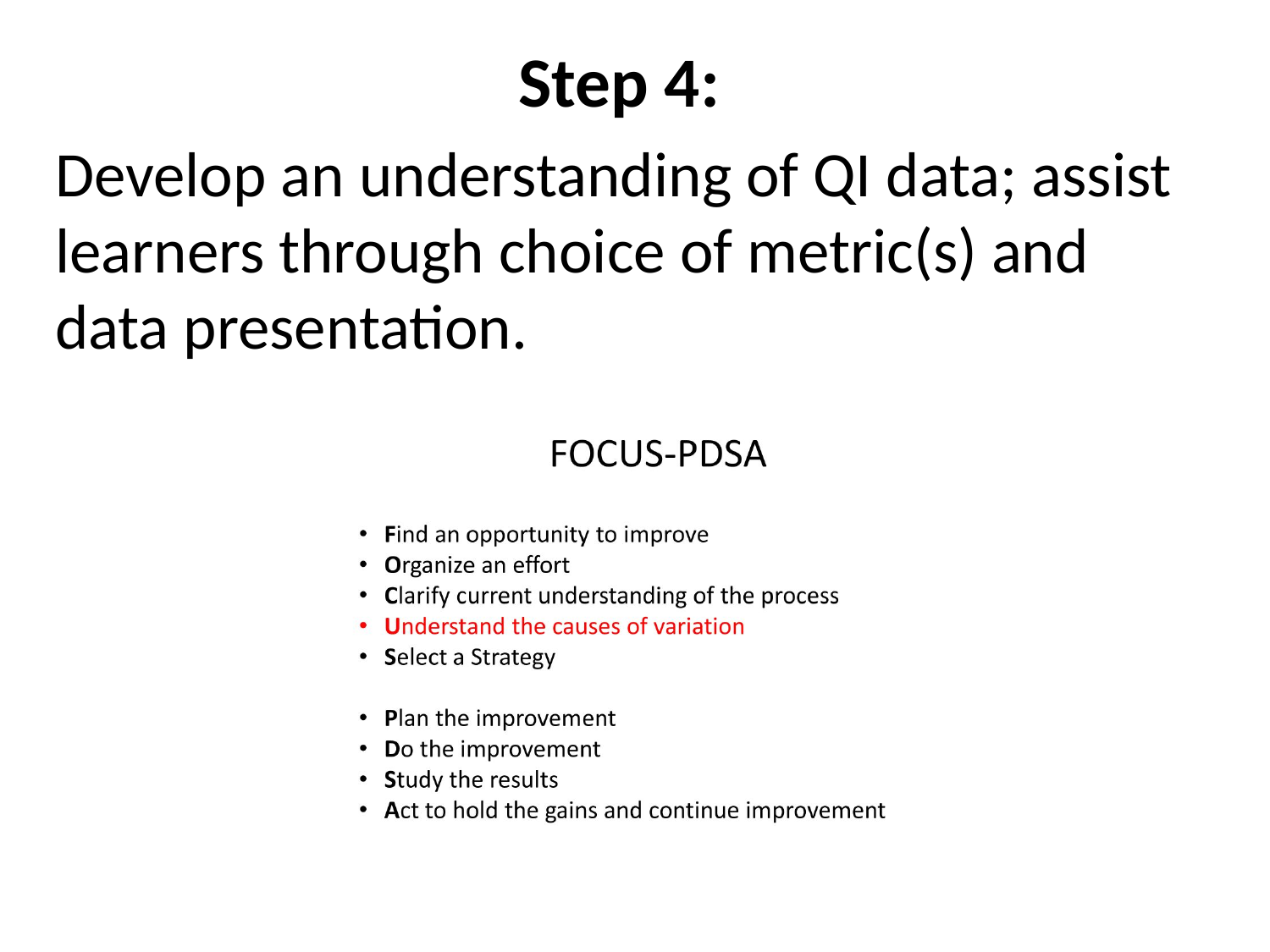

Step 4:
Develop an understanding of QI data; assist learners through choice of metric(s) and data presentation.

## Slide 43
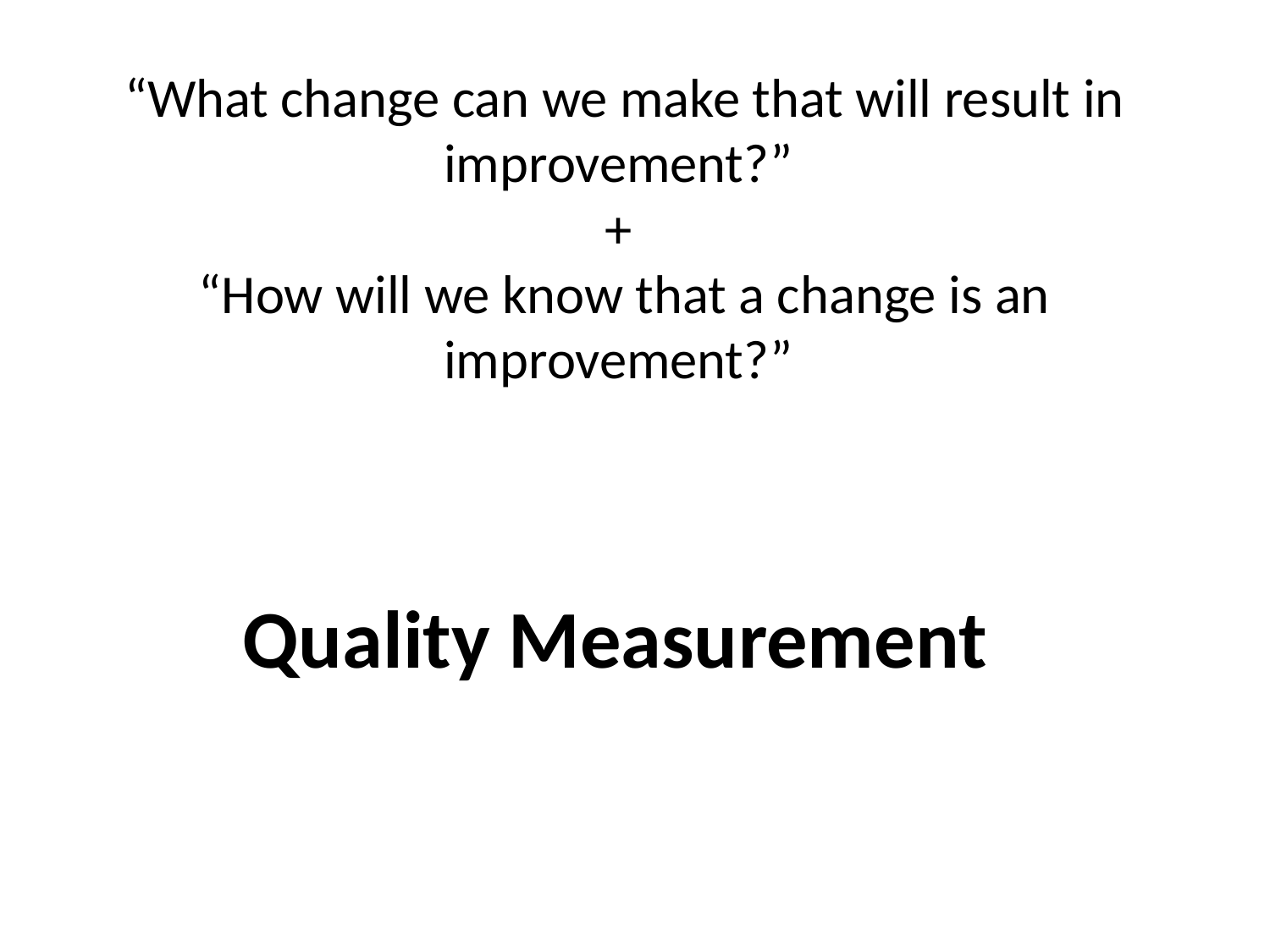

# “What change can we make that will result in improvement?” + “How will we know that a change is an improvement?” Quality Measurement

## Slide 44
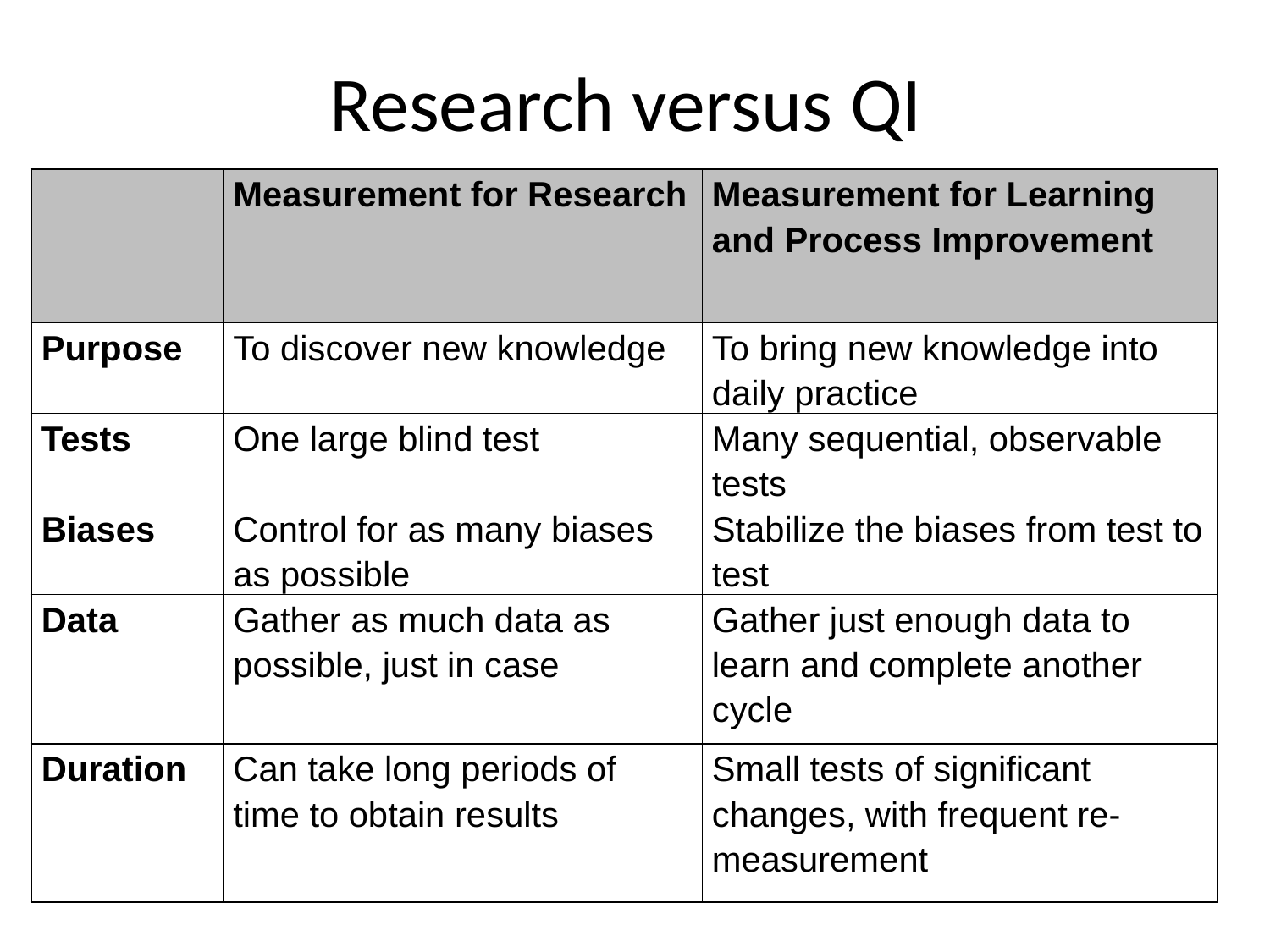

# Research versus QI
| | Measurement for Research | Measurement for Learning and Process Improvement |
| --- | --- | --- |
| Purpose | To discover new knowledge | To bring new knowledge into daily practice |
| Tests | One large blind test | Many sequential, observable tests |
| Biases | Control for as many biases as possible | Stabilize the biases from test to test |
| Data | Gather as much data as possible, just in case | Gather just enough data to learn and complete another cycle |
| Duration | Can take long periods of time to obtain results | Small tests of significant changes, with frequent re-measurement |

## Slide 45
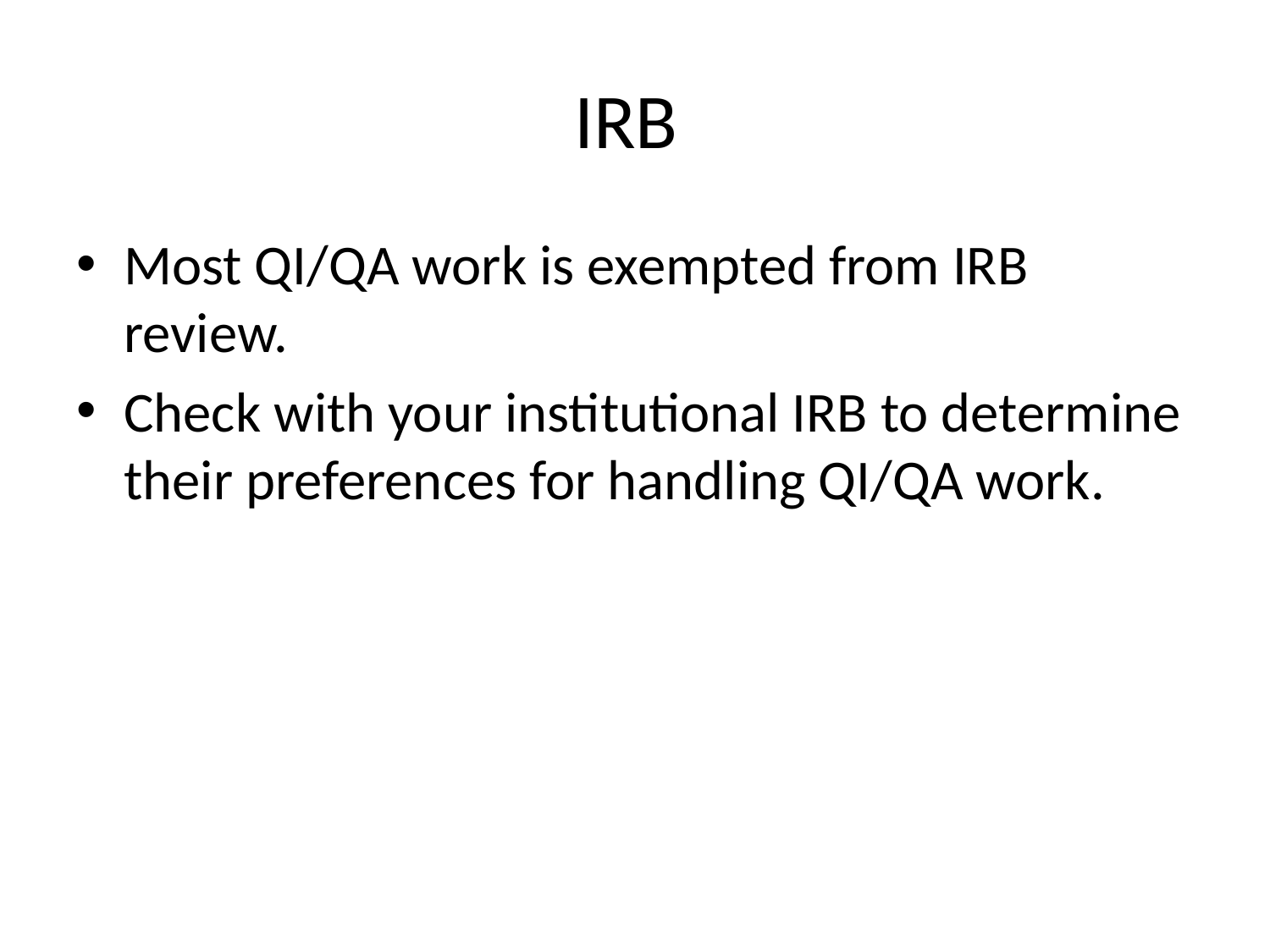

# IRB
Most QI/QA work is exempted from IRB review.
Check with your institutional IRB to determine their preferences for handling QI/QA work.

## Slide 46
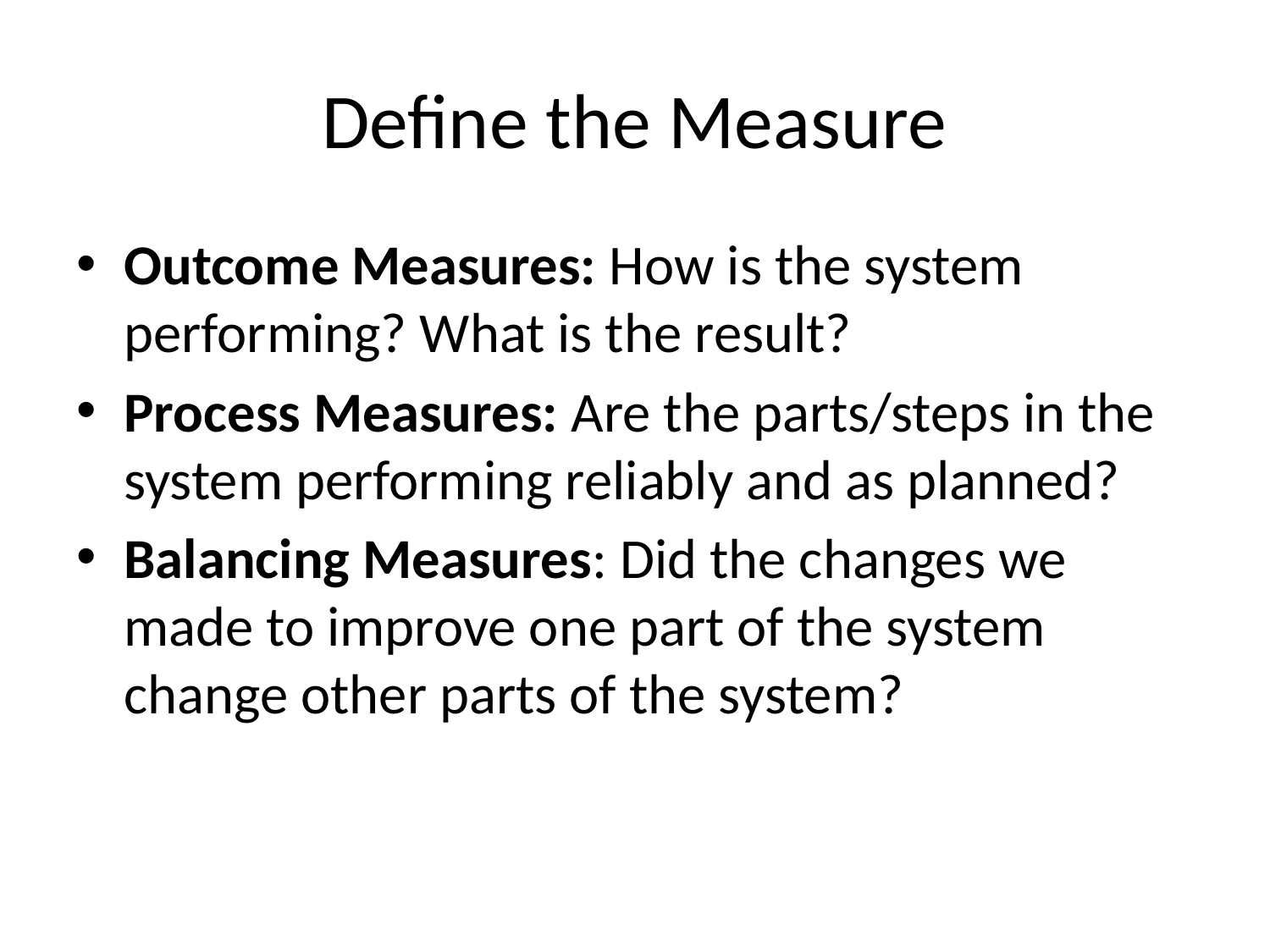

# Define the Measure
Outcome Measures: How is the system performing? What is the result?
Process Measures: Are the parts/steps in the system performing reliably and as planned?
Balancing Measures: Did the changes we made to improve one part of the system change other parts of the system?

## Slide 47
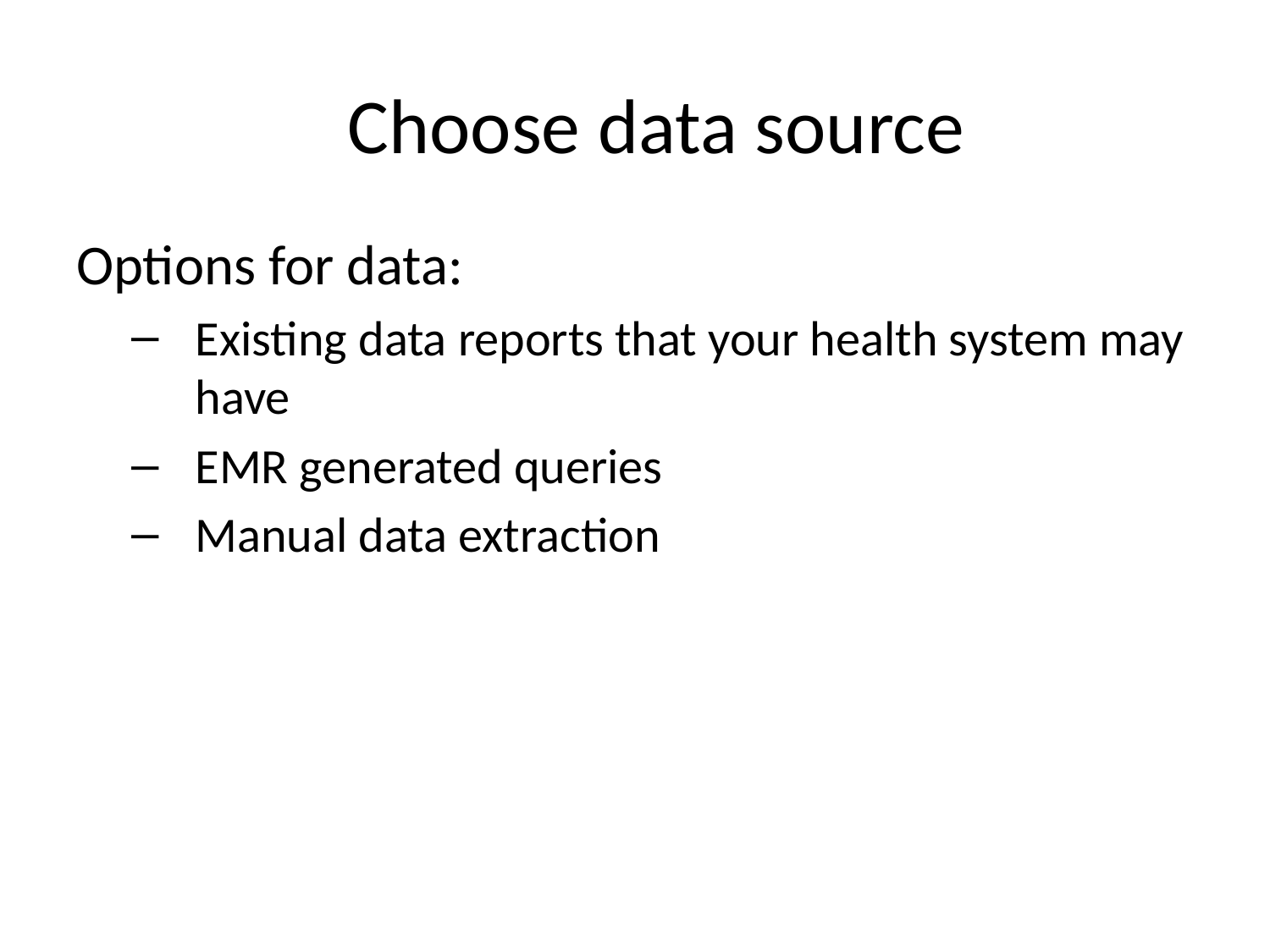

# Choose data source
Options for data:
Existing data reports that your health system may have
EMR generated queries
Manual data extraction

## Slide 48
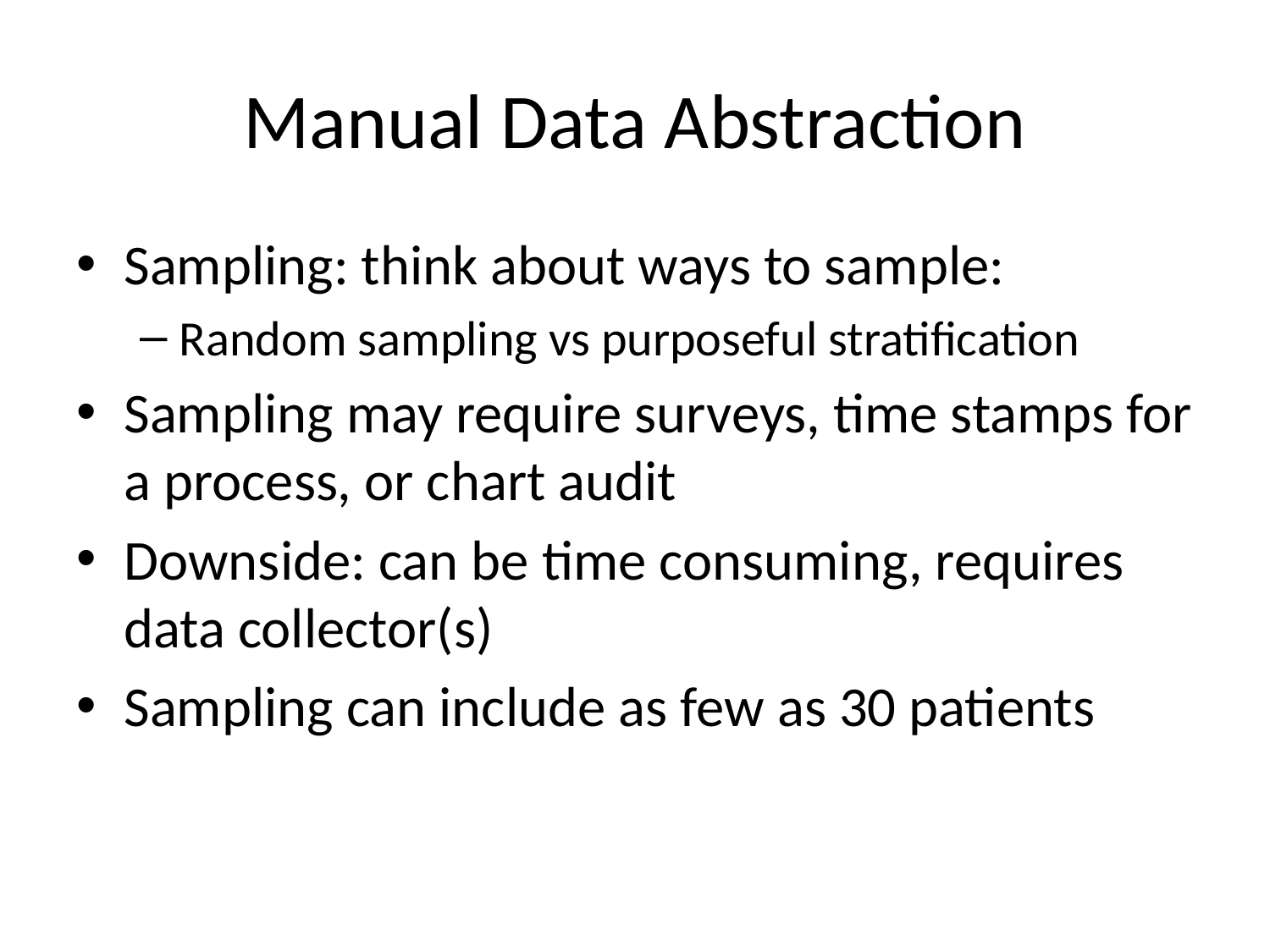

# Manual Data Abstraction
Sampling: think about ways to sample:
Random sampling vs purposeful stratification
Sampling may require surveys, time stamps for a process, or chart audit
Downside: can be time consuming, requires data collector(s)
Sampling can include as few as 30 patients

## Slide 49
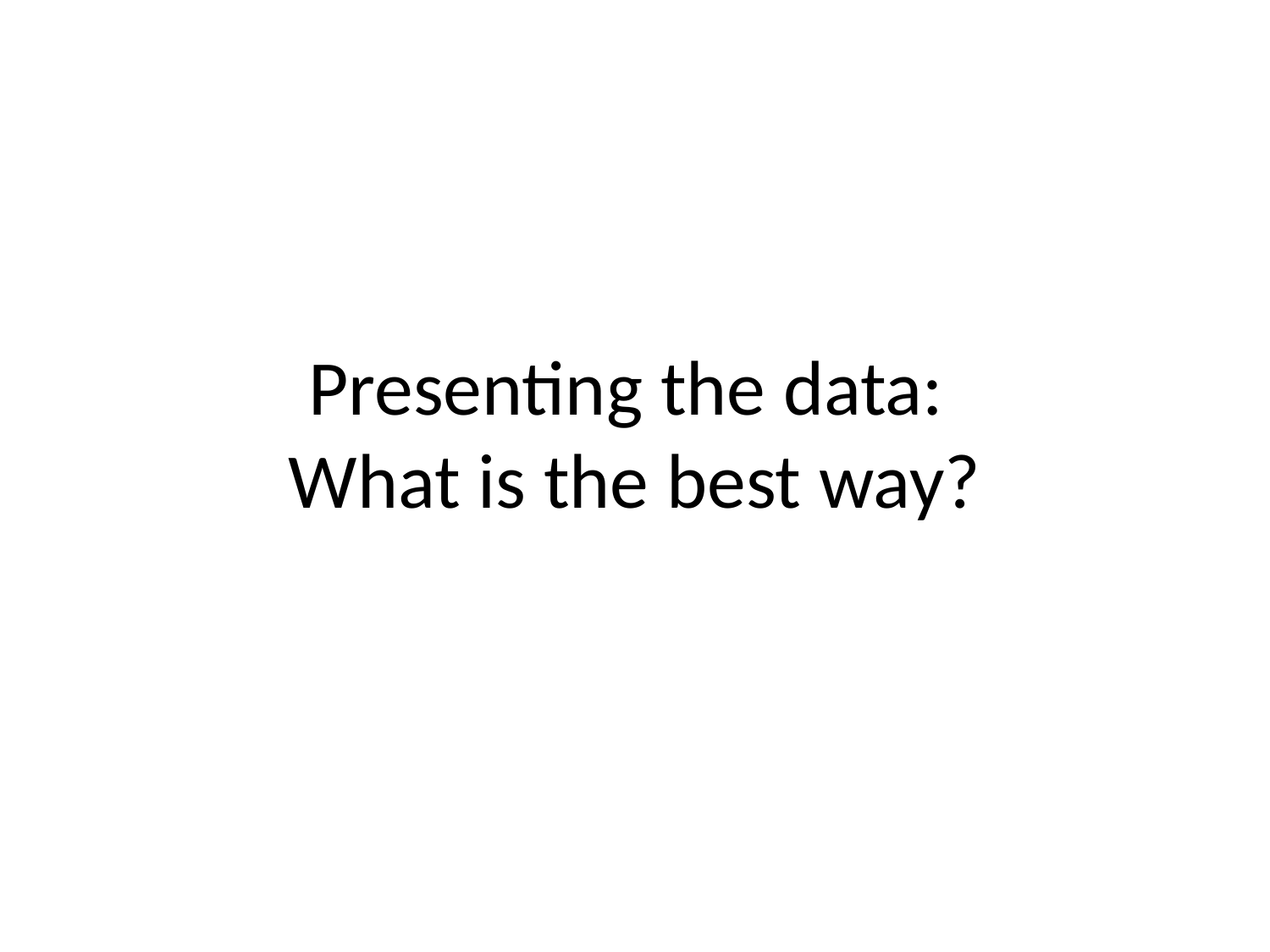

# Presenting the data: What is the best way?

## Slide 50
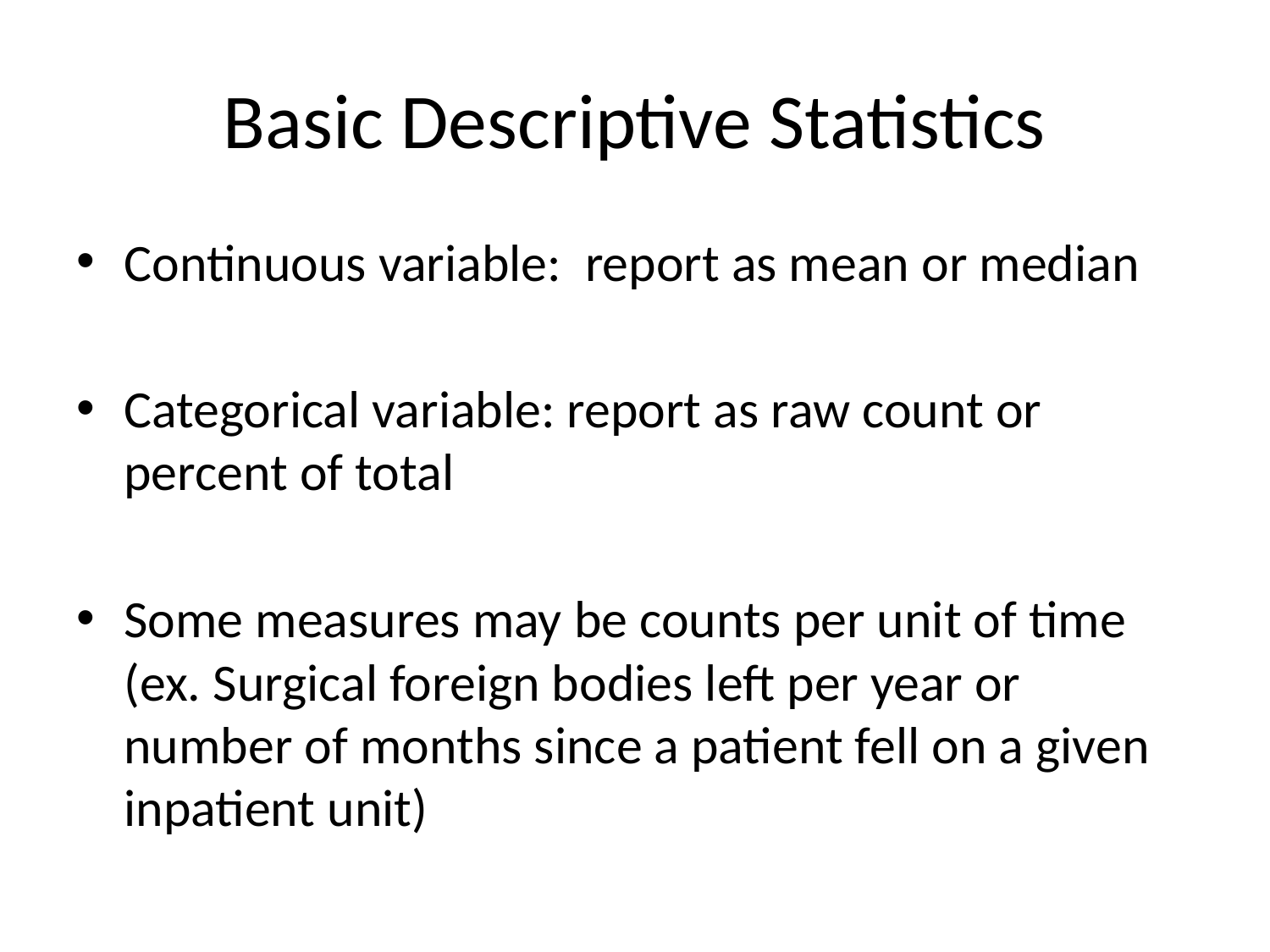

# Basic Descriptive Statistics
Continuous variable: report as mean or median
Categorical variable: report as raw count or percent of total
Some measures may be counts per unit of time   (ex. Surgical foreign bodies left per year or number of months since a patient fell on a given inpatient unit)

## Slide 51
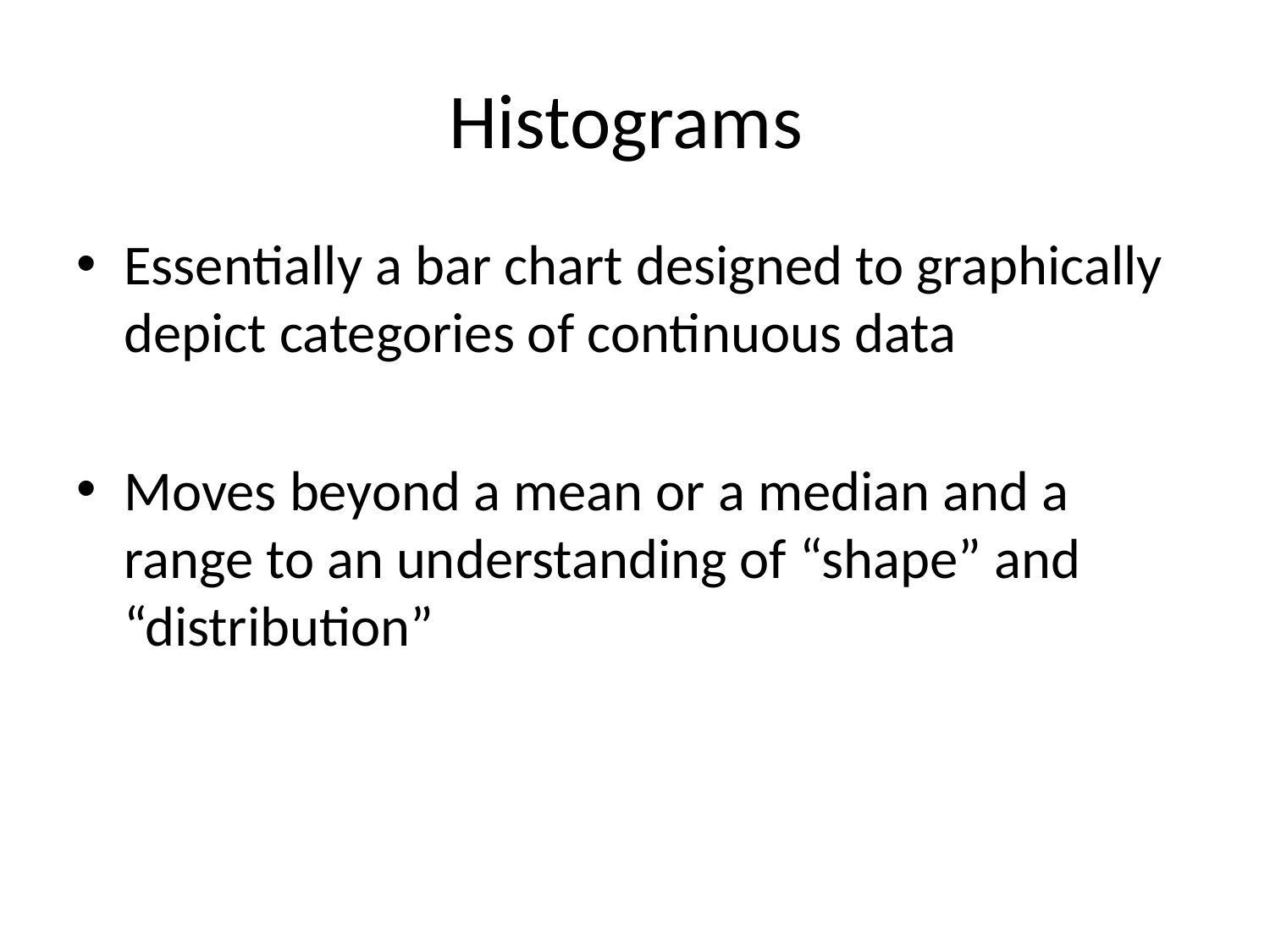

# Histograms
Essentially a bar chart designed to graphically depict categories of continuous data
Moves beyond a mean or a median and a range to an understanding of “shape” and “distribution”

## Slide 52
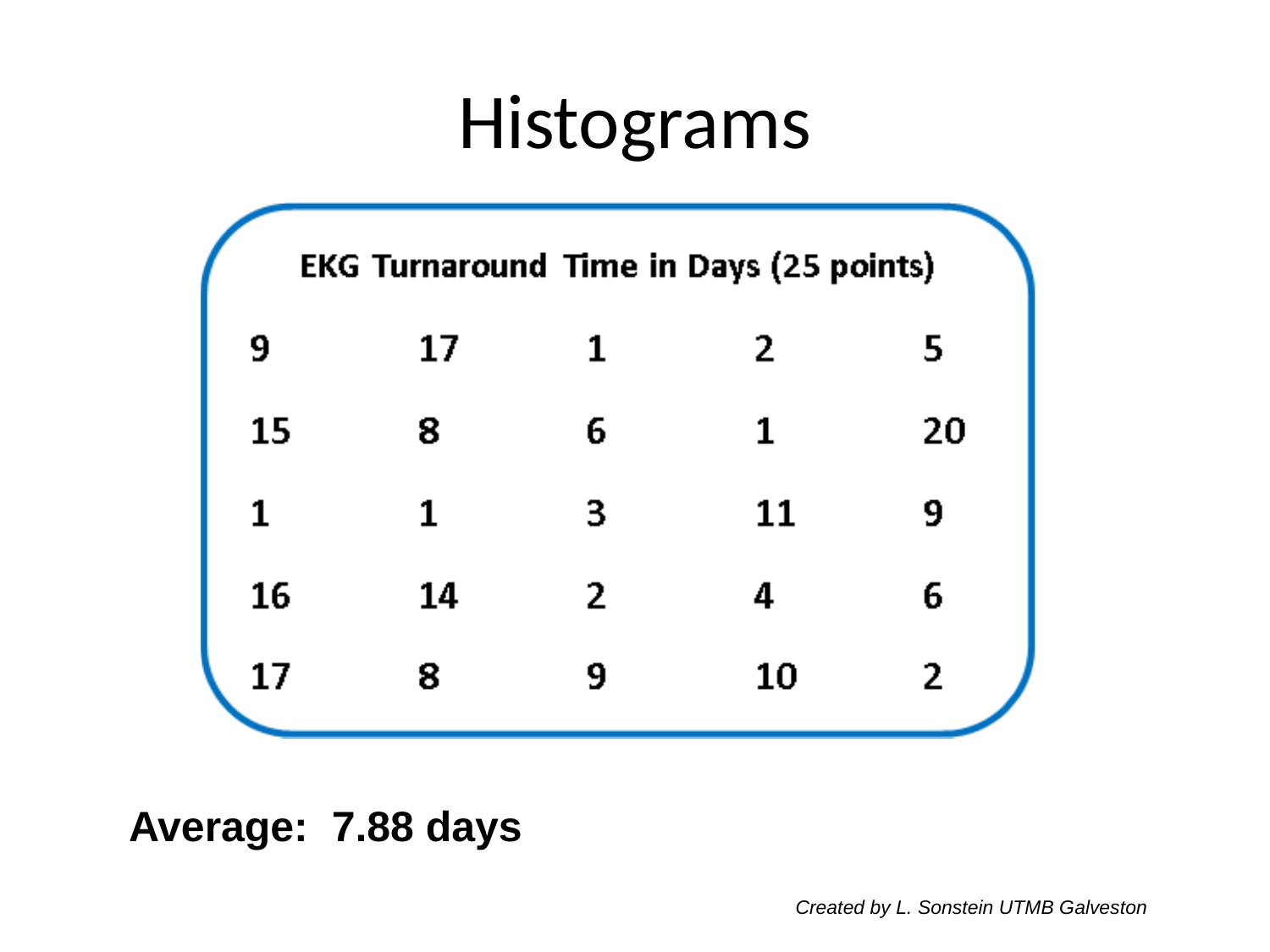

# Histograms
Average: 7.88 days
Created by L. Sonstein UTMB Galveston

## Slide 53
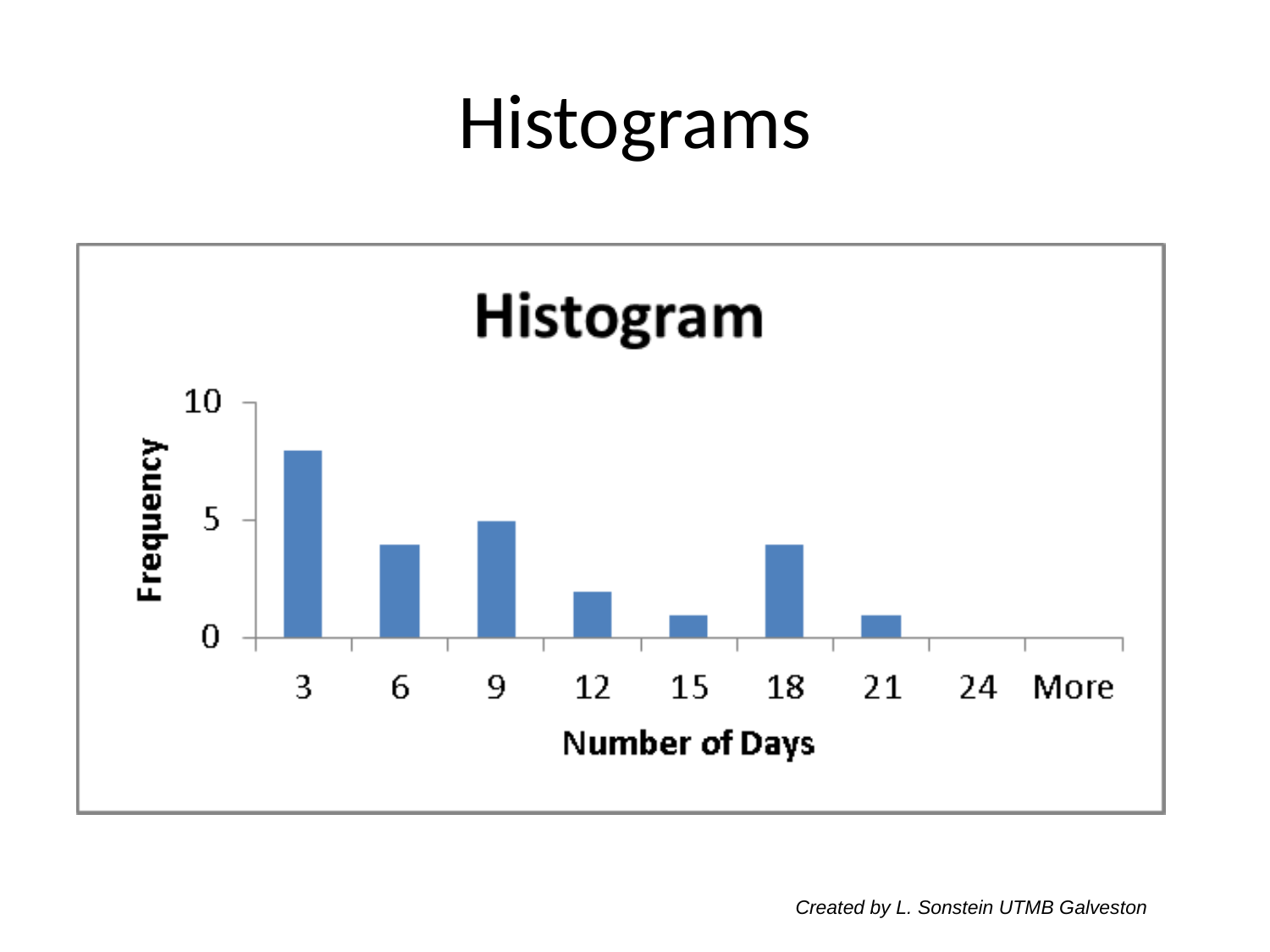

# Histograms
Created by L. Sonstein UTMB Galveston

## Slide 54
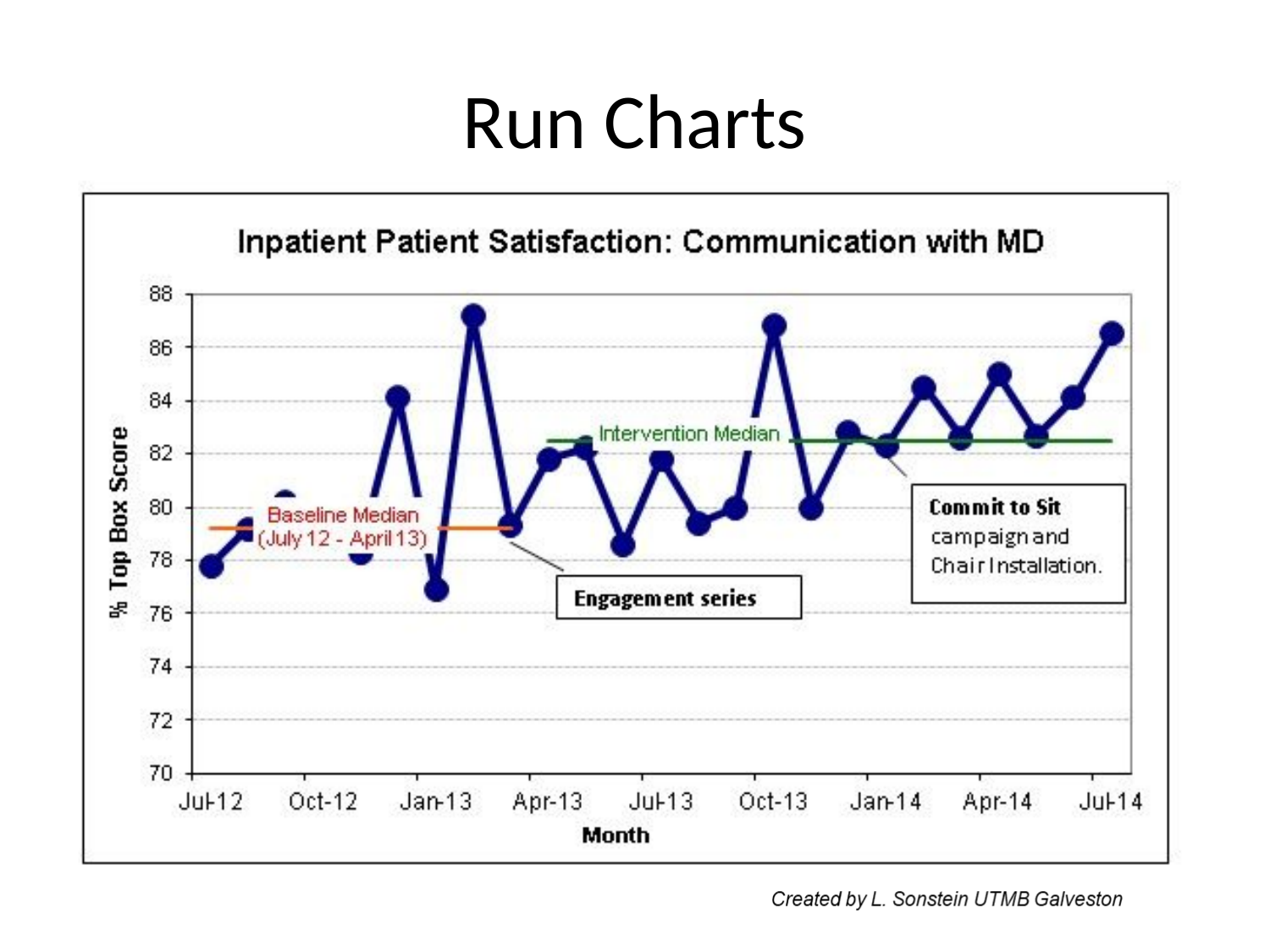

# Run Charts

## Slide 55
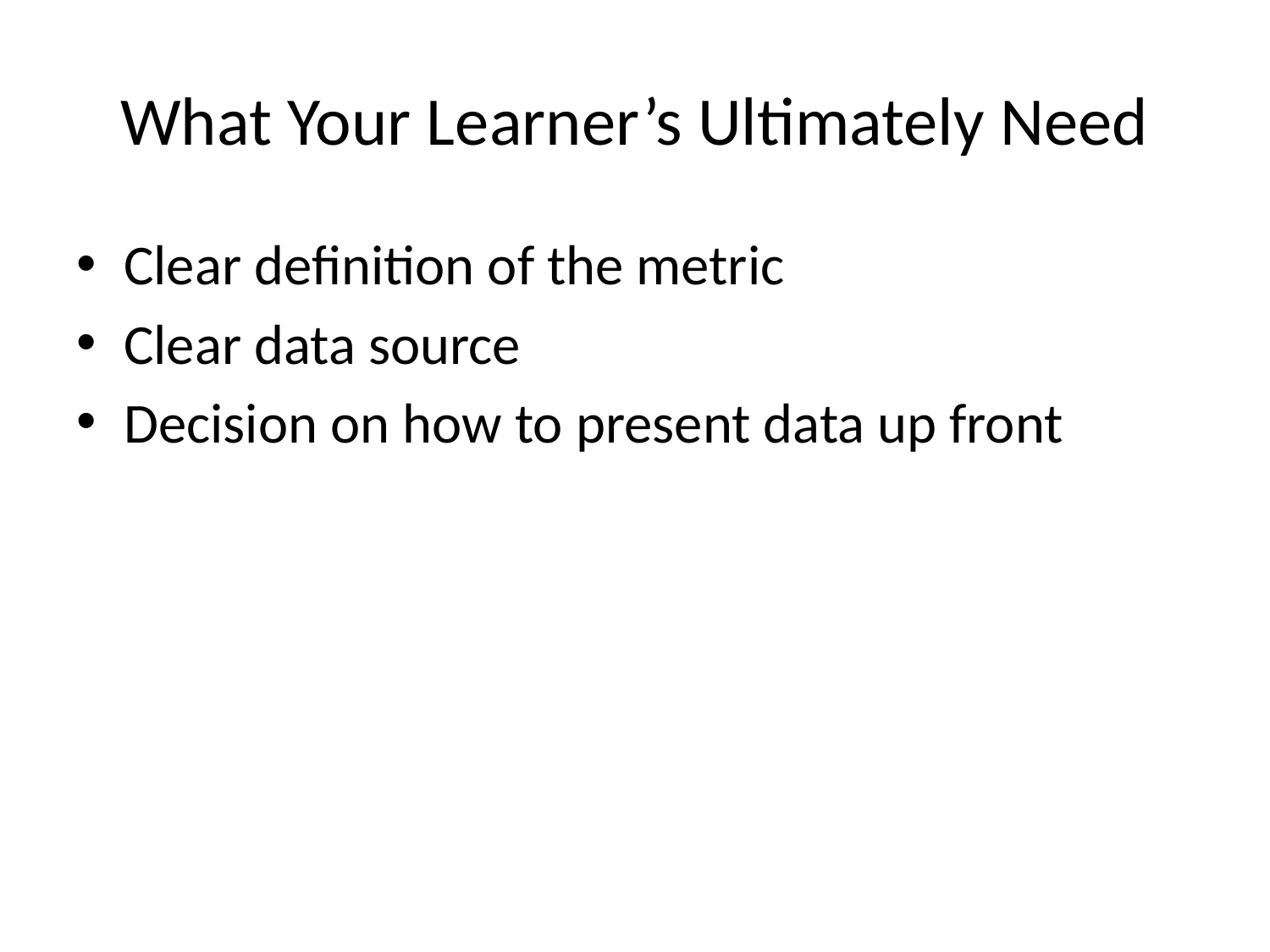

# What Your Learner’s Ultimately Need
Clear definition of the metric
Clear data source
Decision on how to present data up front

## Slide 56
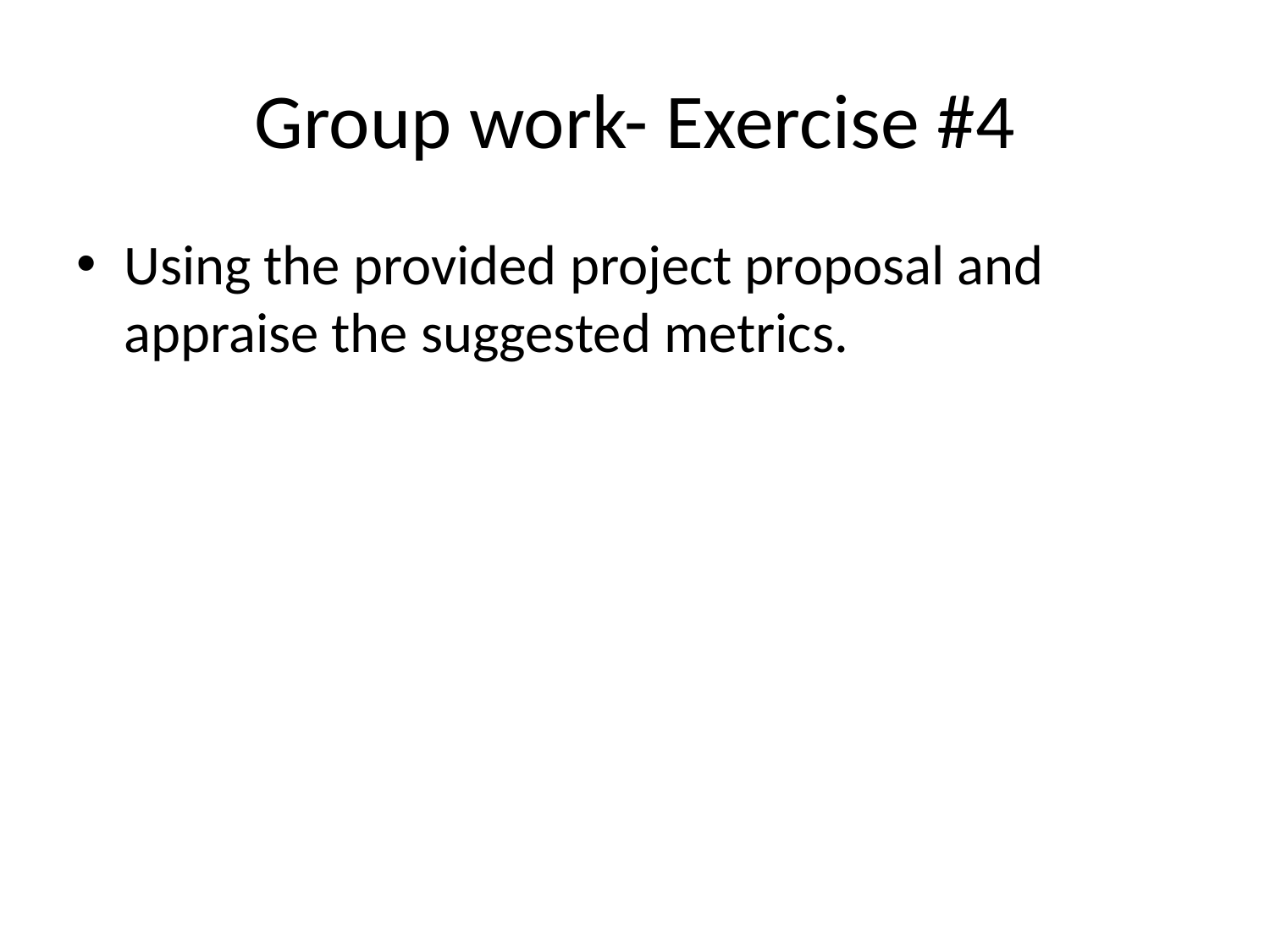

# Group work- Exercise #4
Using the provided project proposal and appraise the suggested metrics.

## Slide 57
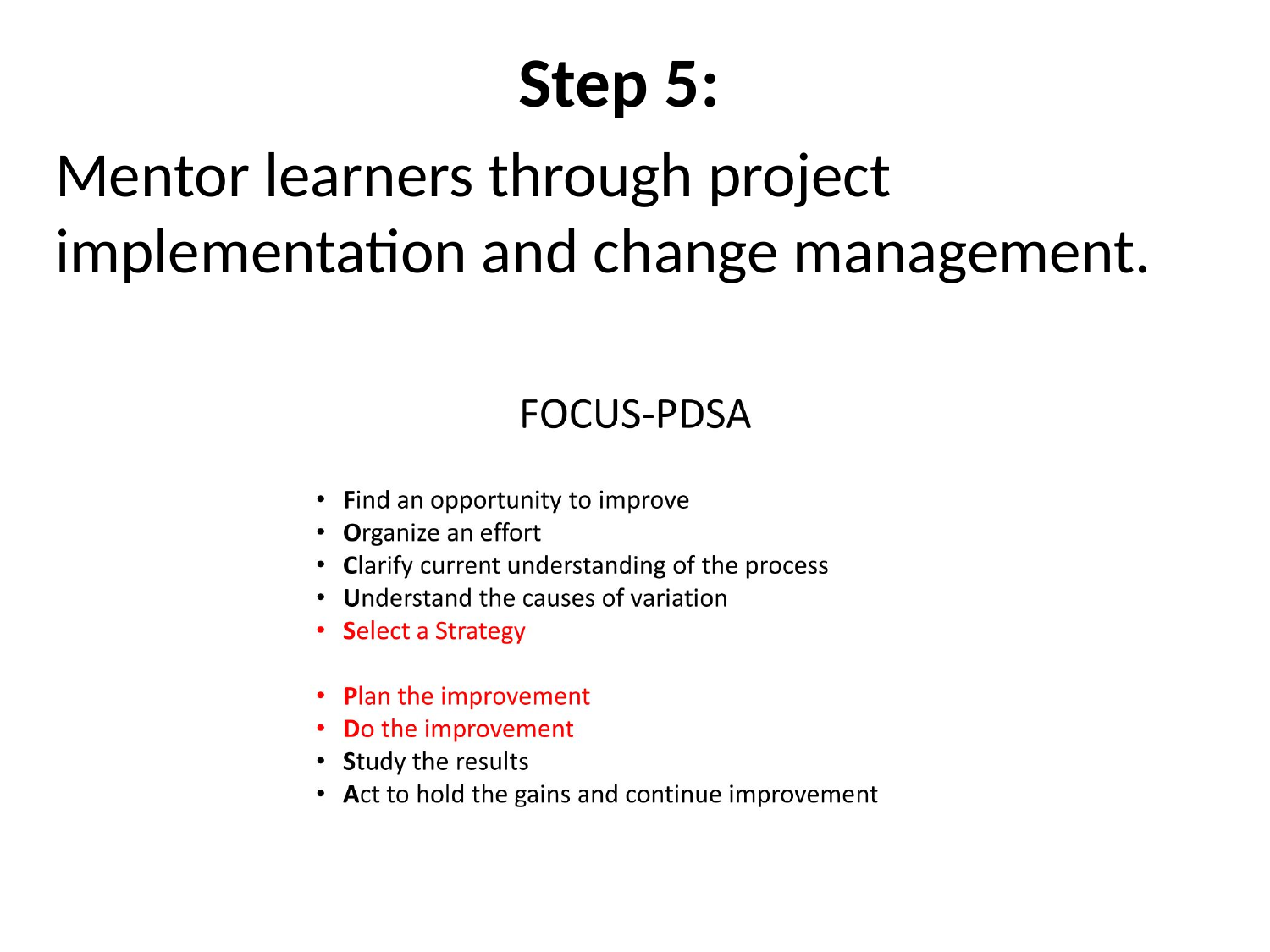

Step 5:
Mentor learners through project implementation and change management.

## Slide 58
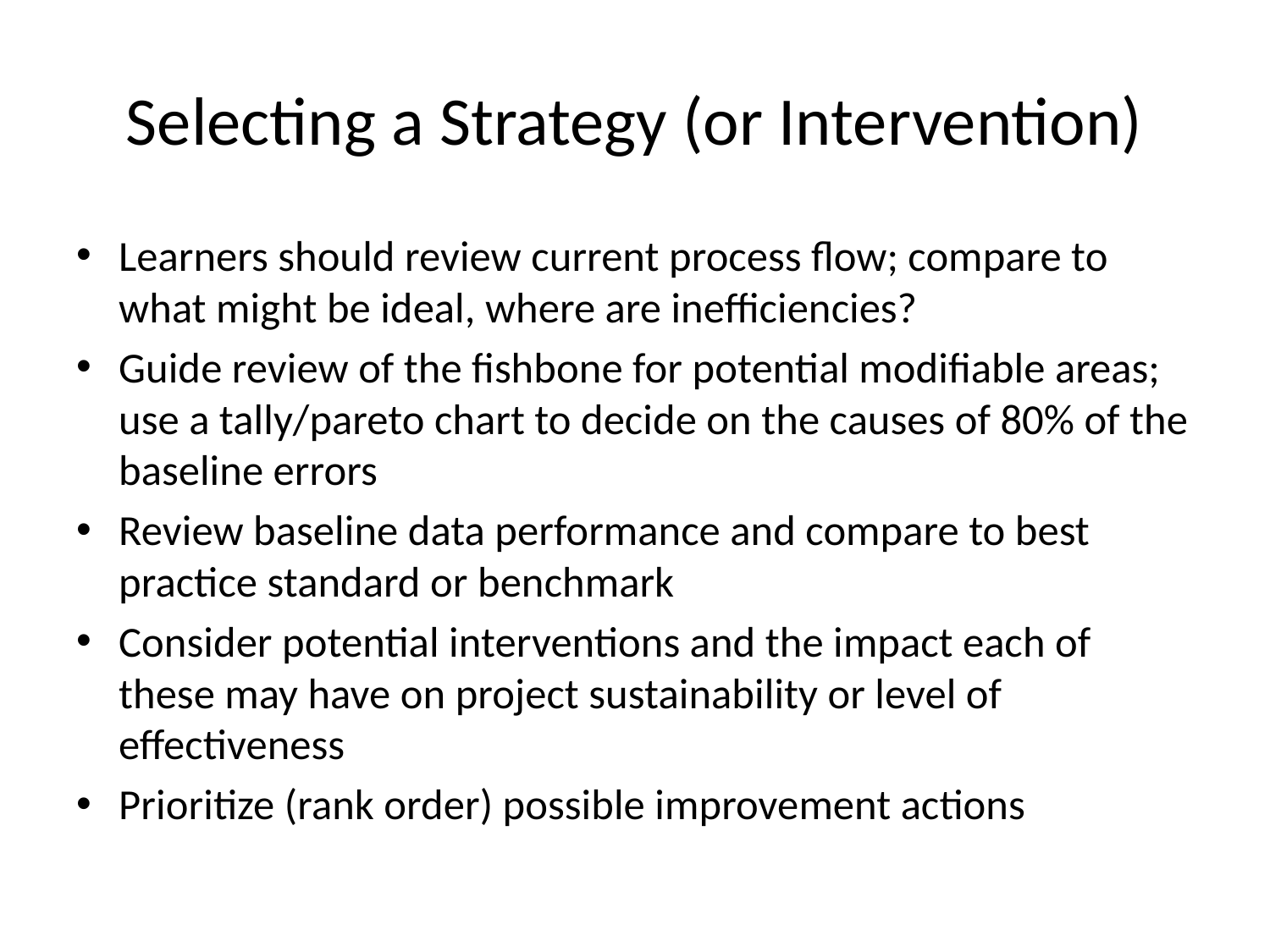

# Selecting a Strategy (or Intervention)
Learners should review current process flow; compare to what might be ideal, where are inefficiencies?
Guide review of the fishbone for potential modifiable areas; use a tally/pareto chart to decide on the causes of 80% of the baseline errors
Review baseline data performance and compare to best practice standard or benchmark
Consider potential interventions and the impact each of these may have on project sustainability or level of effectiveness
Prioritize (rank order) possible improvement actions

## Slide 59
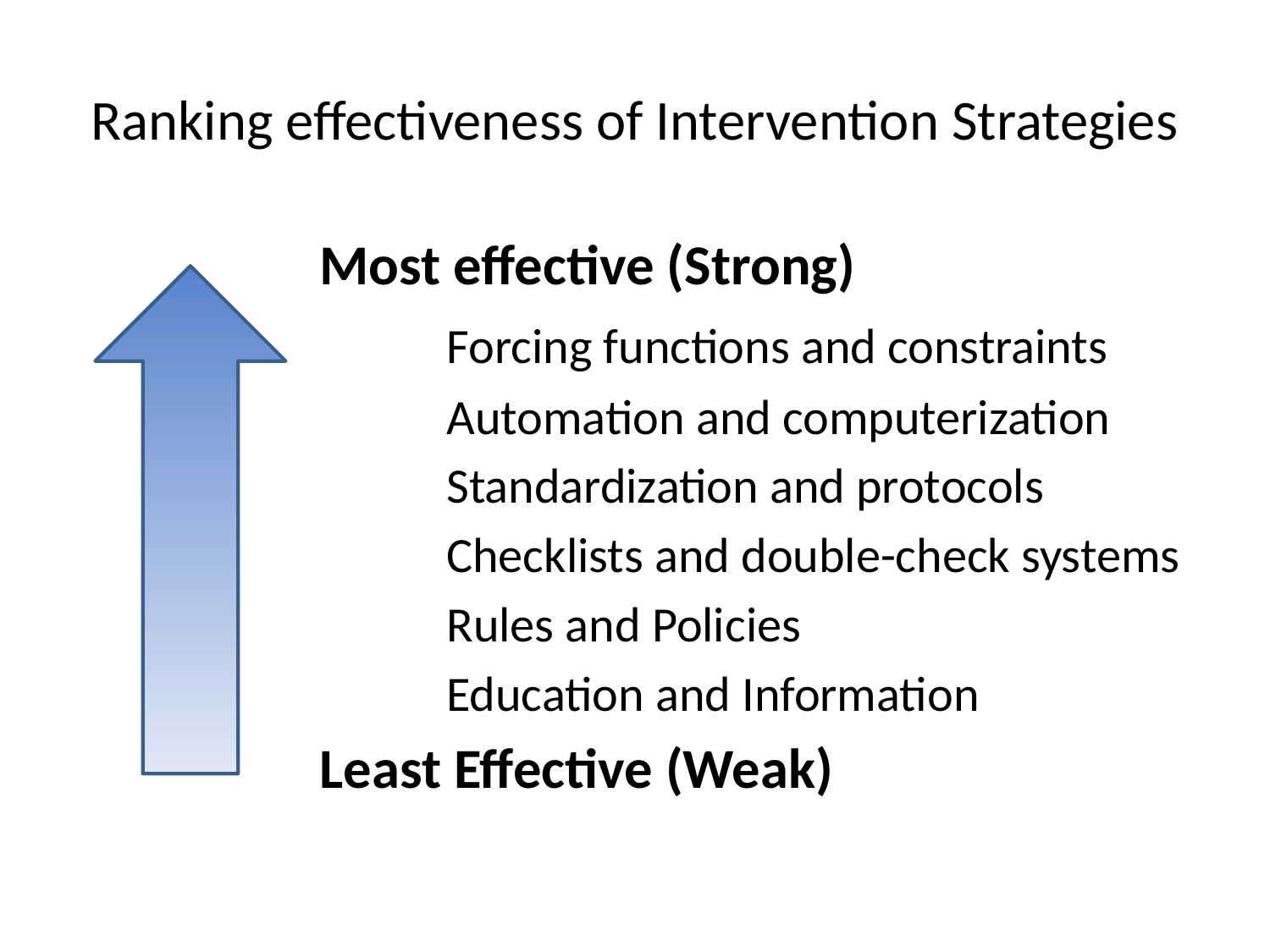

# Ranking effectiveness of Intervention Strategies
		Most effective (Strong)
			Forcing functions and constraints
			Automation and computerization
			Standardization and protocols
			Checklists and double-check systems
			Rules and Policies
			Education and Information
		Least Effective (Weak)

## Slide 60
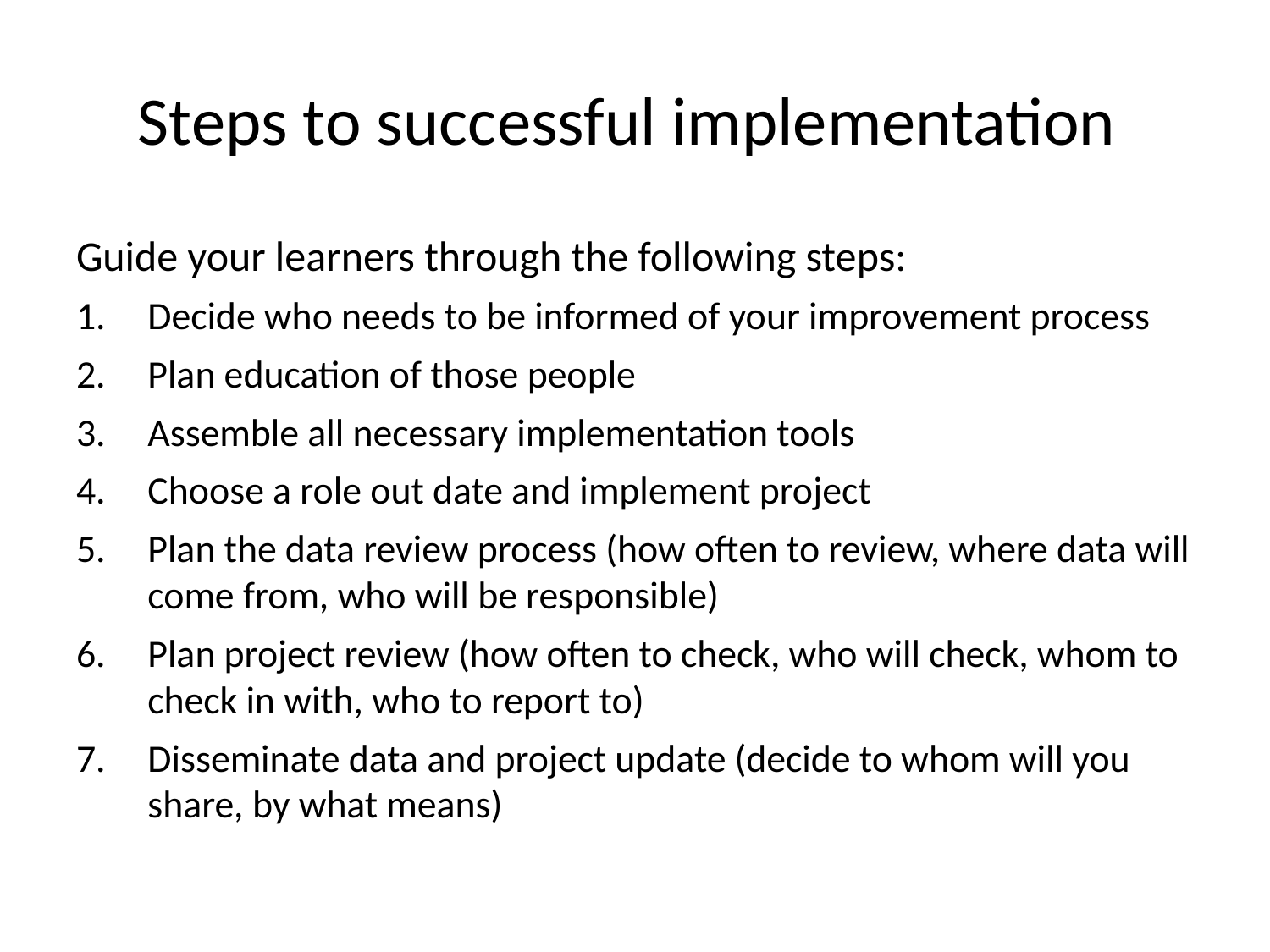

# Steps to successful implementation
Guide your learners through the following steps:
Decide who needs to be informed of your improvement process
Plan education of those people
Assemble all necessary implementation tools
Choose a role out date and implement project
Plan the data review process (how often to review, where data will come from, who will be responsible)
Plan project review (how often to check, who will check, whom to check in with, who to report to)
Disseminate data and project update (decide to whom will you share, by what means)

## Slide 61
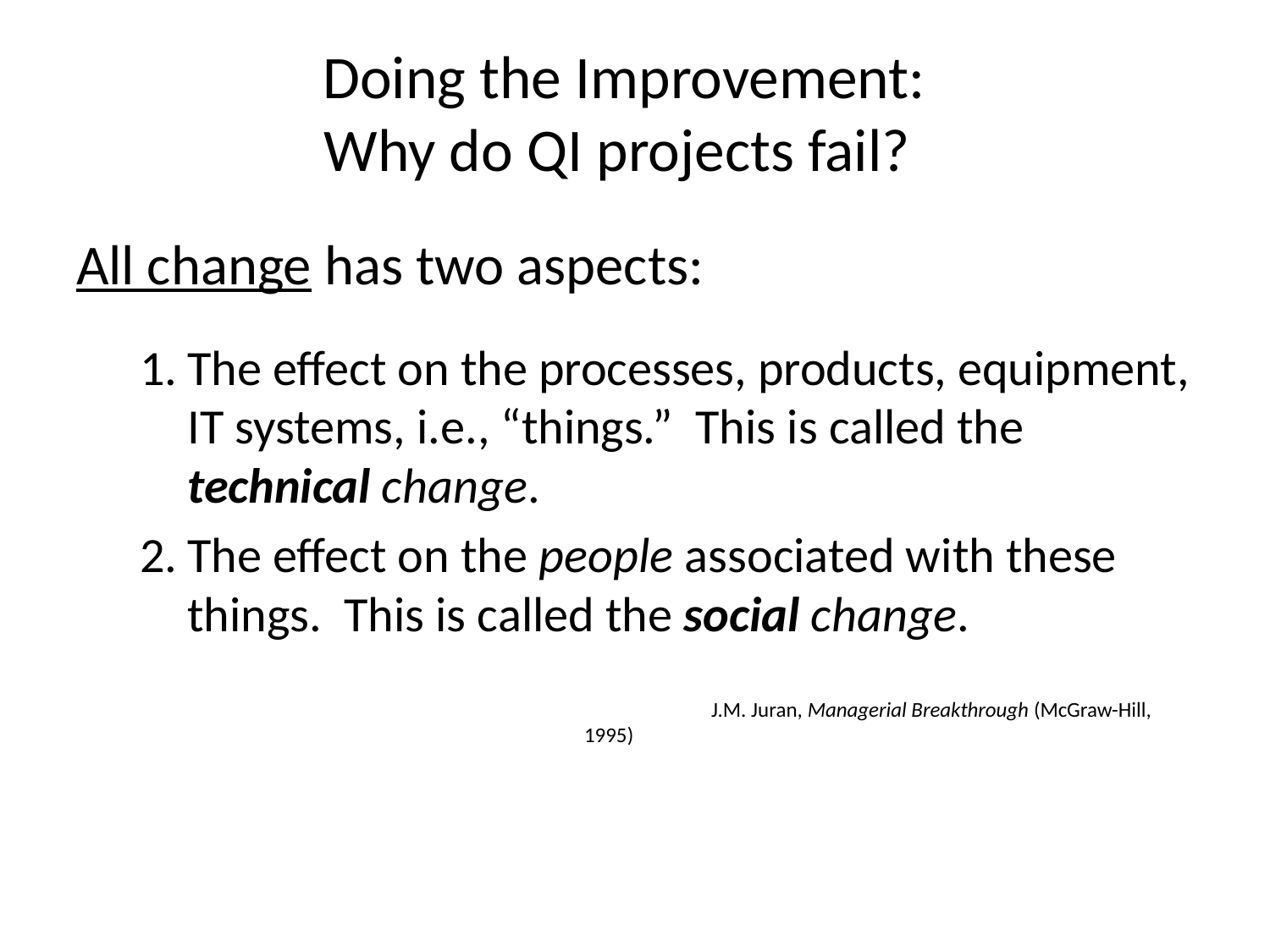

# Doing the Improvement: Why do QI projects fail?
All change has two aspects:
The effect on the processes, products, equipment, IT systems, i.e., “things.” This is called the technical change.
The effect on the people associated with these things. This is called the social change.
	J.M. Juran, Managerial Breakthrough (McGraw-Hill, 1995)

## Slide 62
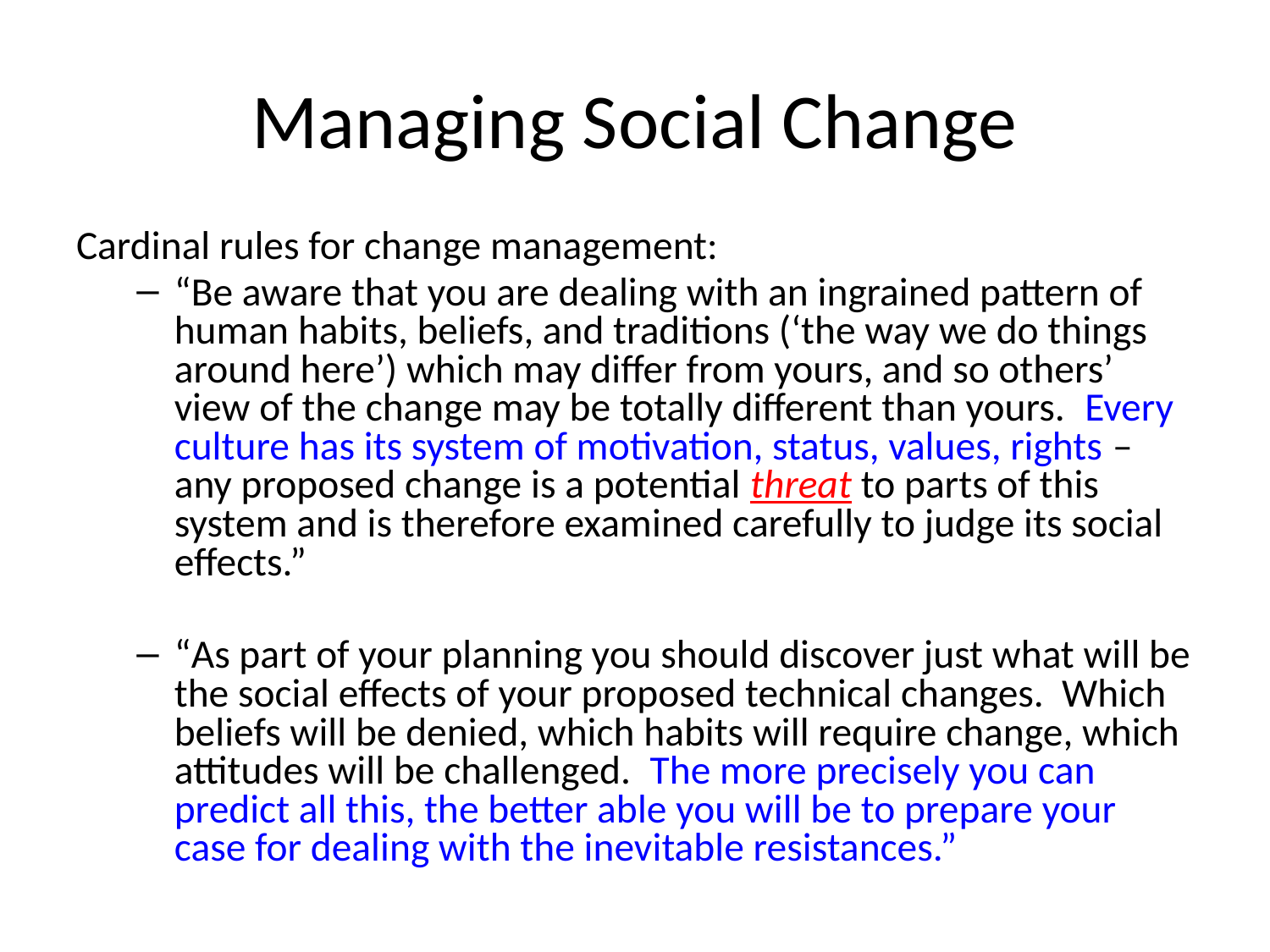

# Managing Social Change
Cardinal rules for change management:
“Be aware that you are dealing with an ingrained pattern of human habits, beliefs, and traditions (‘the way we do things around here’) which may differ from yours, and so others’ view of the change may be totally different than yours. Every culture has its system of motivation, status, values, rights – any proposed change is a potential threat to parts of this system and is therefore examined carefully to judge its social effects.”
“As part of your planning you should discover just what will be the social effects of your proposed technical changes. Which beliefs will be denied, which habits will require change, which attitudes will be challenged. The more precisely you can predict all this, the better able you will be to prepare your case for dealing with the inevitable resistances.”

## Slide 63
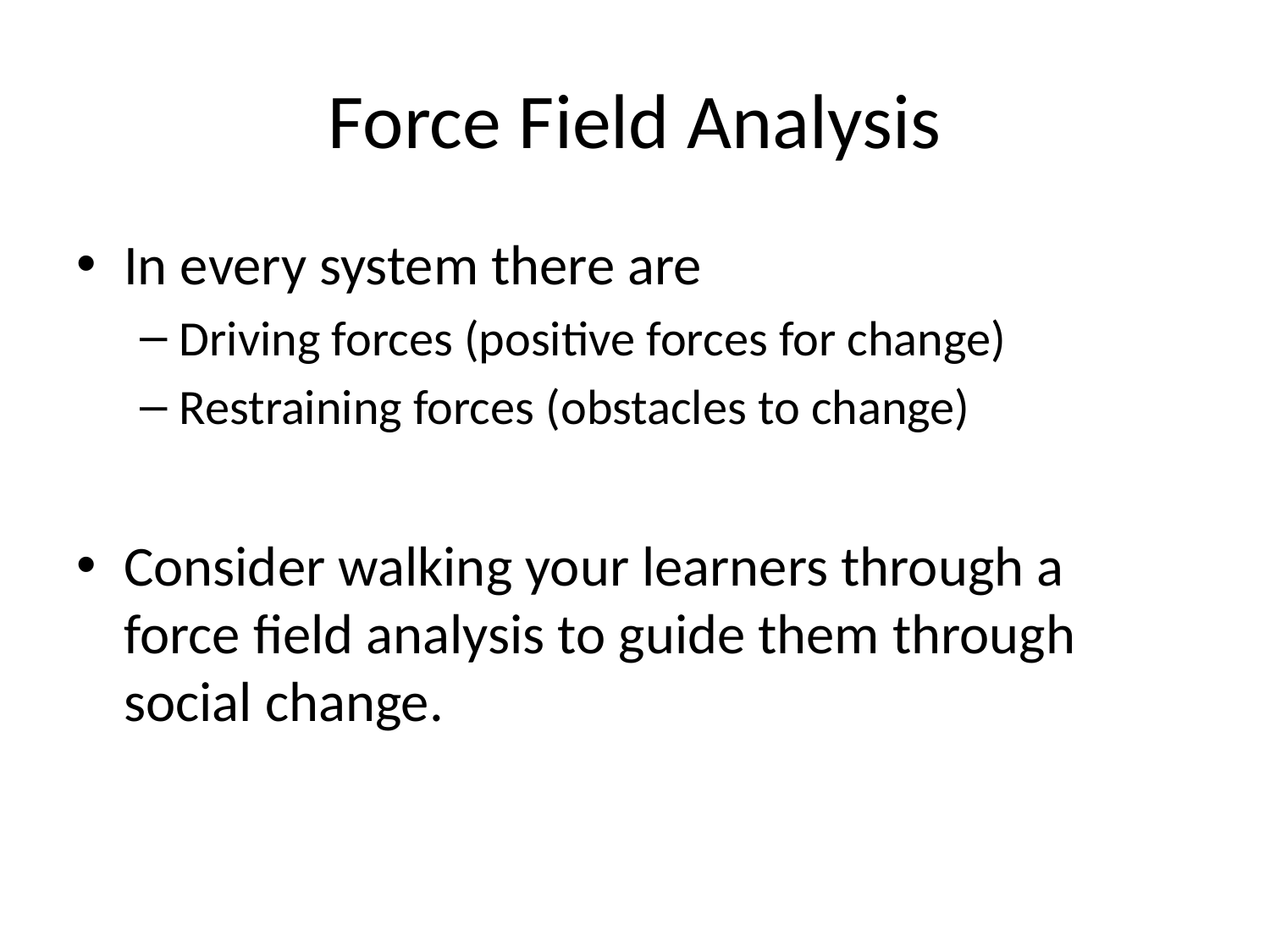

# Force Field Analysis
In every system there are
Driving forces (positive forces for change)
Restraining forces (obstacles to change)
Consider walking your learners through a force field analysis to guide them through social change.

## Slide 64
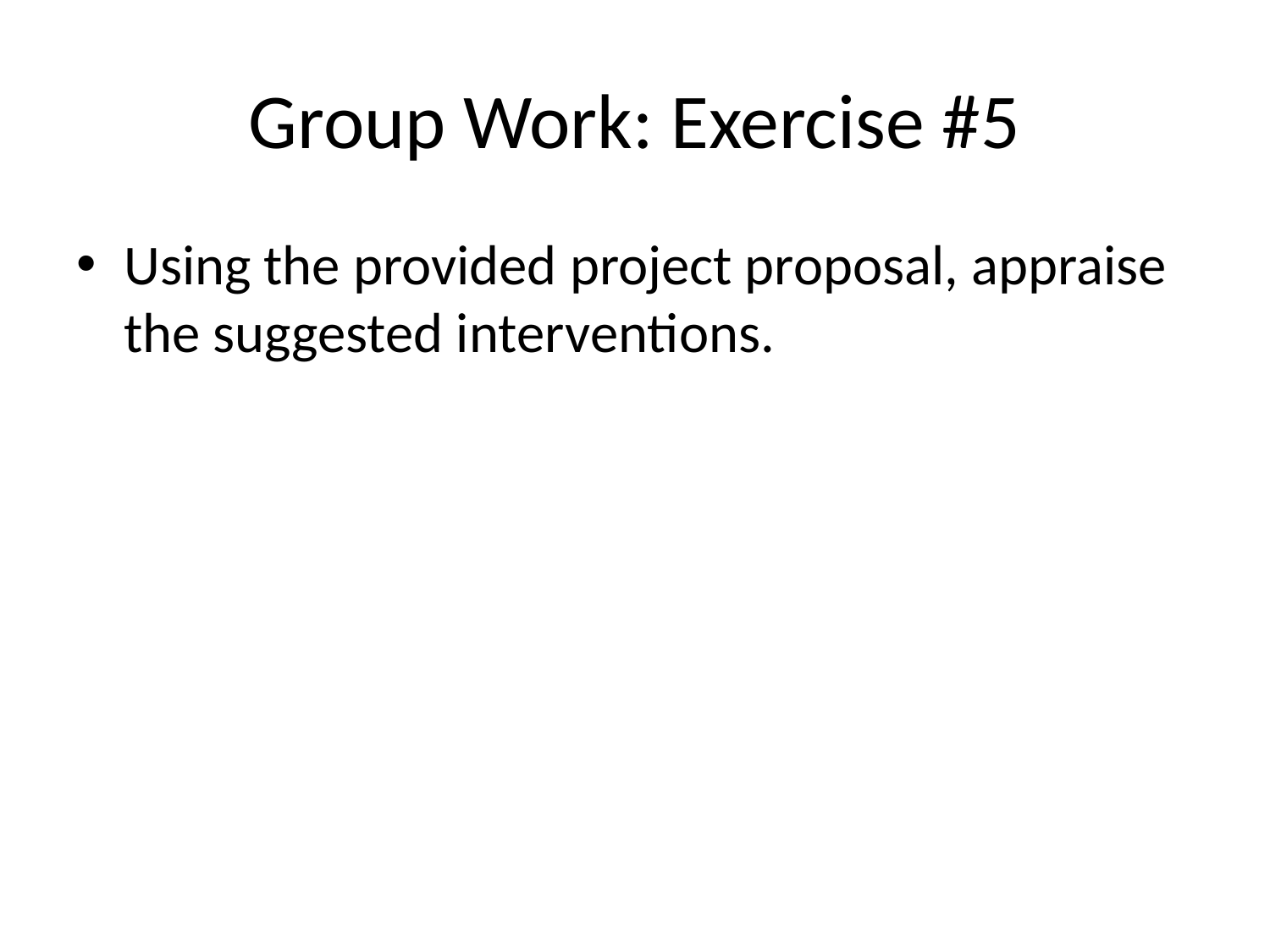

# Group Work: Exercise #5
Using the provided project proposal, appraise the suggested interventions.

## Slide 65
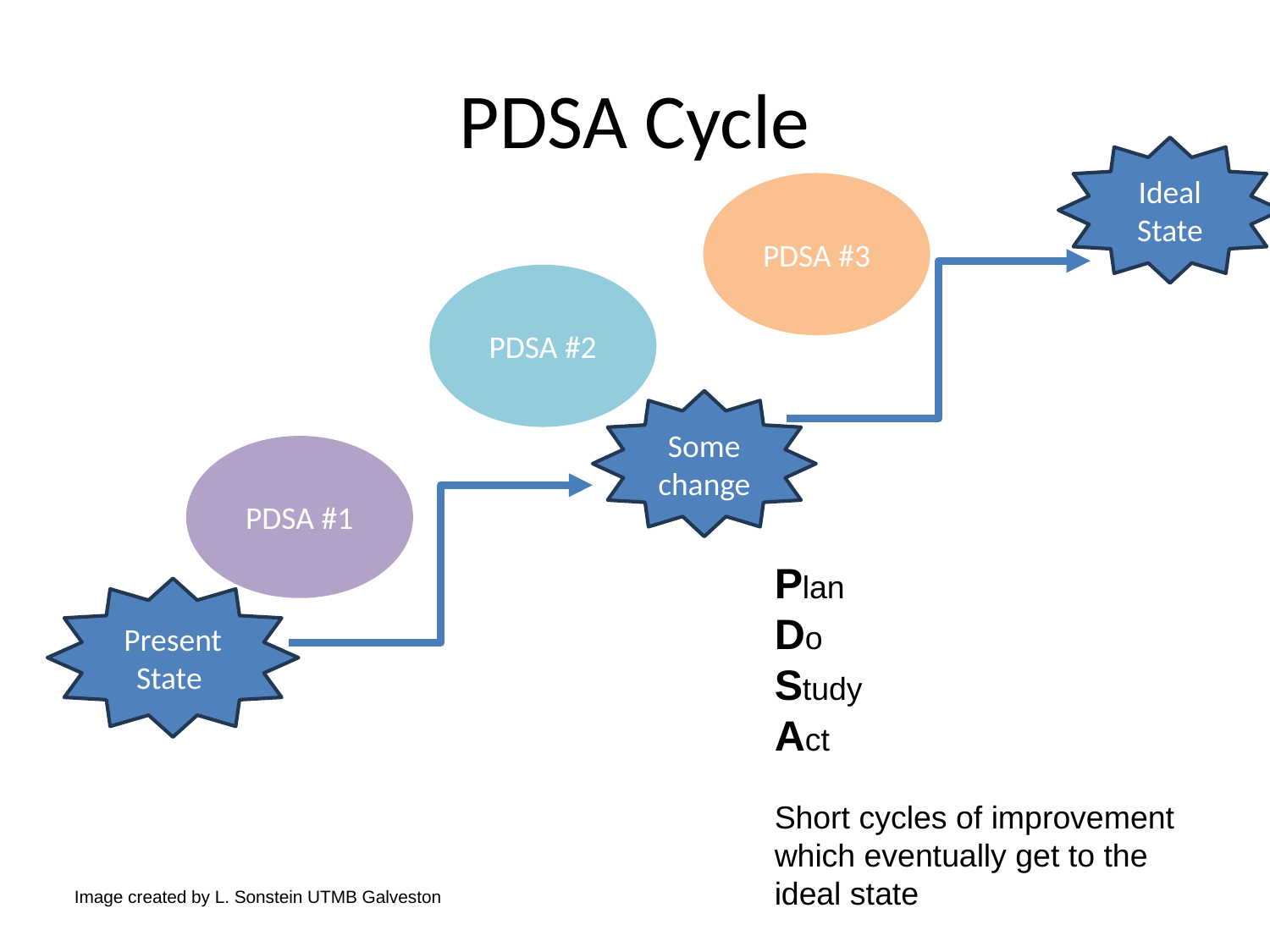

# PDSA Cycle
Ideal State
PDSA #3
PDSA #2
Some change
PDSA #1
Plan
Do
Study
Act
Short cycles of improvement which eventually get to the ideal state
Present State
Image created by L. Sonstein UTMB Galveston

## Slide 66
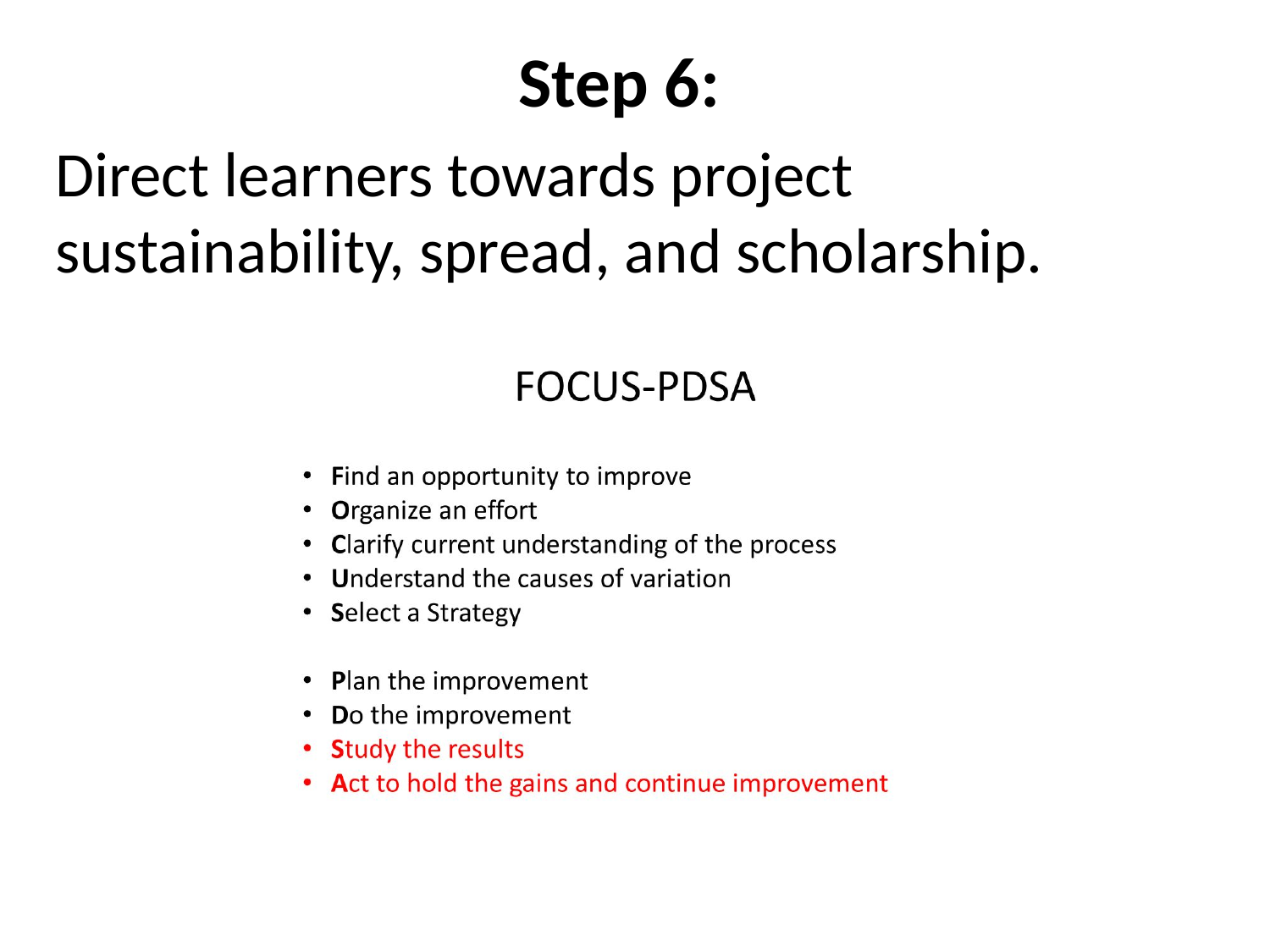

Step 6:
Direct learners towards project sustainability, spread, and scholarship.

## Slide 67
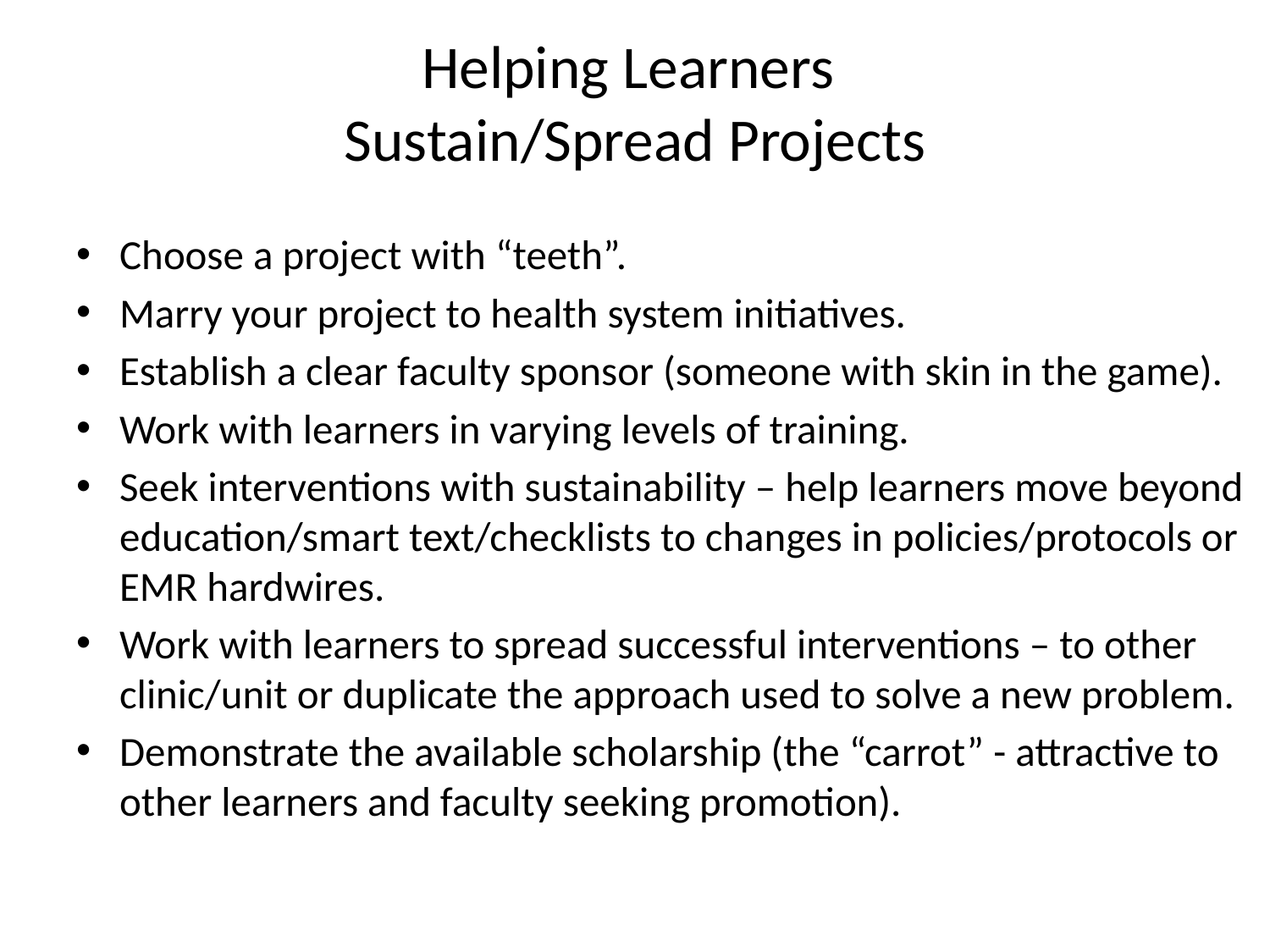

# Helping Learners Sustain/Spread Projects
Choose a project with “teeth”.
Marry your project to health system initiatives.
Establish a clear faculty sponsor (someone with skin in the game).
Work with learners in varying levels of training.
Seek interventions with sustainability – help learners move beyond education/smart text/checklists to changes in policies/protocols or EMR hardwires.
Work with learners to spread successful interventions – to other clinic/unit or duplicate the approach used to solve a new problem.
Demonstrate the available scholarship (the “carrot” - attractive to other learners and faculty seeking promotion).

## Slide 68
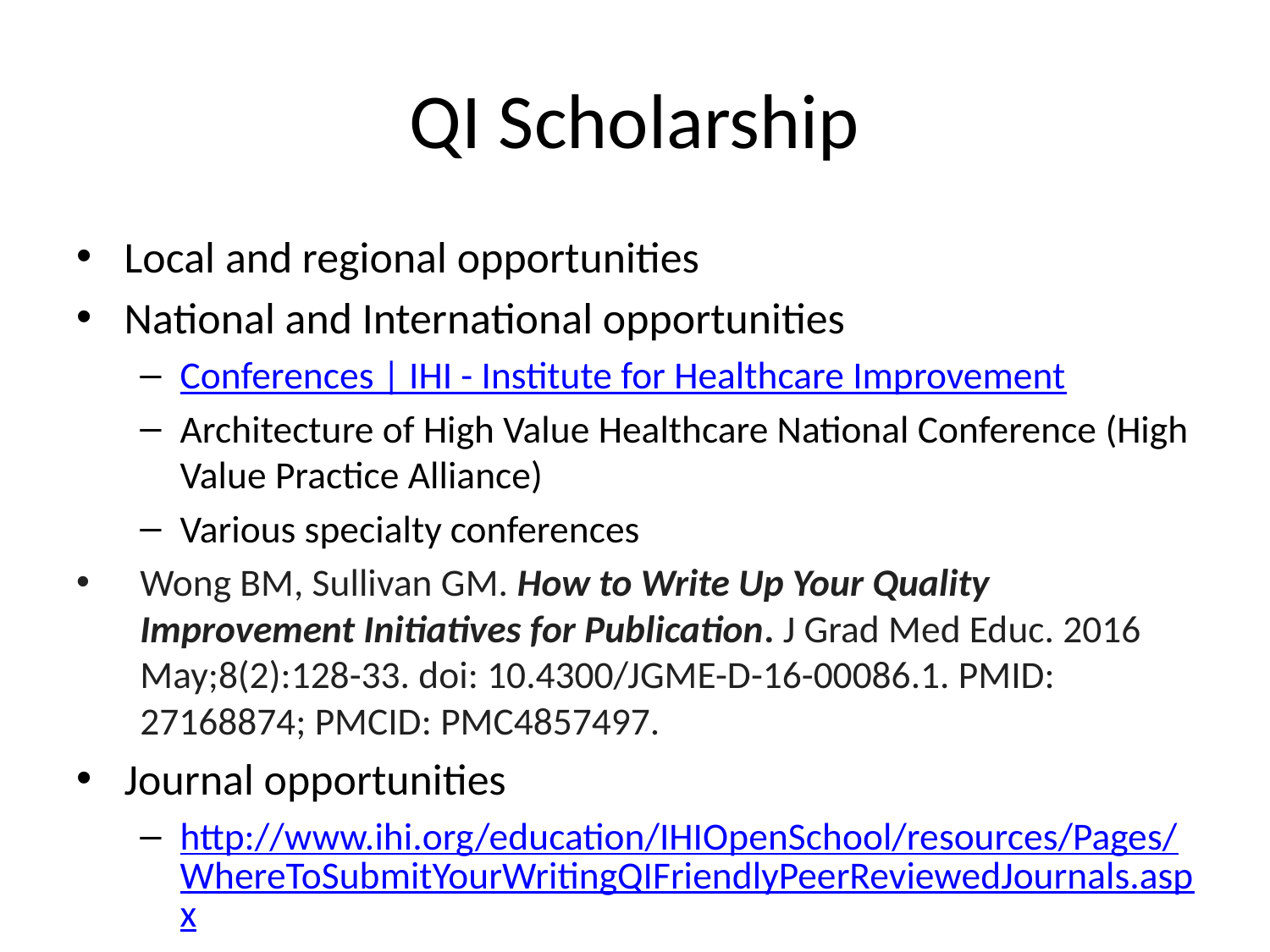

# QI Scholarship
Local and regional opportunities
National and International opportunities
Conferences | IHI - Institute for Healthcare Improvement
Architecture of High Value Healthcare National Conference (High Value Practice Alliance)
Various specialty conferences
Wong BM, Sullivan GM. How to Write Up Your Quality Improvement Initiatives for Publication. J Grad Med Educ. 2016 May;8(2):128-33. doi: 10.4300/JGME-D-16-00086.1. PMID: 27168874; PMCID: PMC4857497.
Journal opportunities
http://www.ihi.org/education/IHIOpenSchool/resources/Pages/WhereToSubmitYourWritingQIFriendlyPeerReviewedJournals.aspx
SQUIRE Guidelines (standard for QI reporting)

## Slide 69
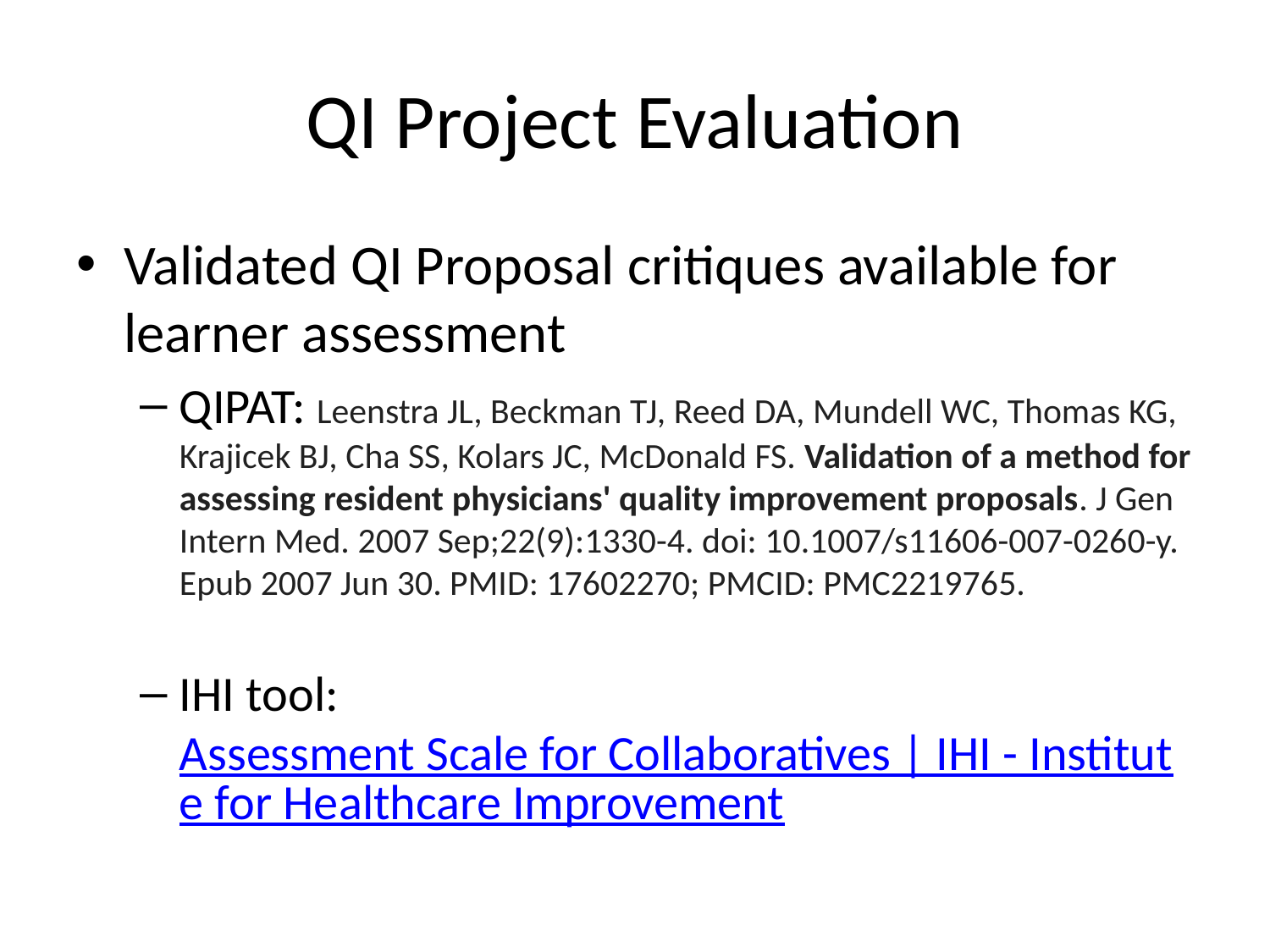

# QI Project Evaluation
Validated QI Proposal critiques available for learner assessment
QIPAT: Leenstra JL, Beckman TJ, Reed DA, Mundell WC, Thomas KG, Krajicek BJ, Cha SS, Kolars JC, McDonald FS. Validation of a method for assessing resident physicians' quality improvement proposals. J Gen Intern Med. 2007 Sep;22(9):1330-4. doi: 10.1007/s11606-007-0260-y. Epub 2007 Jun 30. PMID: 17602270; PMCID: PMC2219765.
IHI tool: Assessment Scale for Collaboratives | IHI - Institute for Healthcare Improvement

## Slide 70
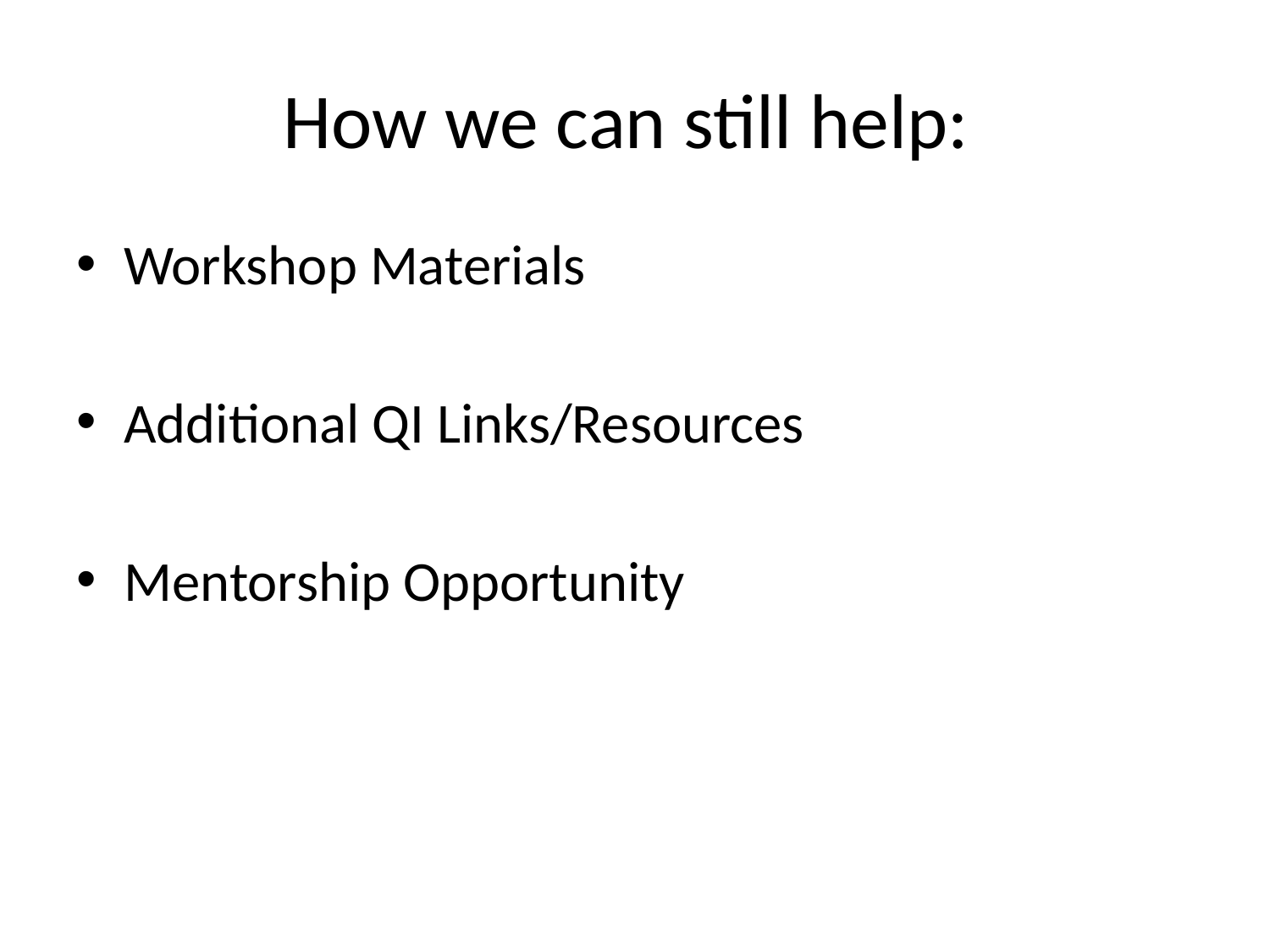

# How we can still help:
Workshop Materials
Additional QI Links/Resources
Mentorship Opportunity
