# Supplementary material for: Development of a Train-the-Trainer Quality Improvement Curriculum
Source: MedEdPORTAL. 2024 Jul 16;20:11425. doi: 10.15766/mep_2374-8265.11425 (PMC11249715; doi:10.15766/mep_2374-8265.11425)
Supplement: Supplementary file 1 — Train-the-Trainer Slide Set.pptxExercise 1 Aim Statements.docxExercise 2 Stakeholder Analysis.docxExercise 3a Flowchart Critique.docxExercise 3b Fishbone Critique.docxExercise 4 Measures Critique.docxExercise 5 Intervention Critique.docxExercise 1 Aim Statements Facilitator Guide.docxExercise 2 Stakeholder Analysis Facilitator Guide.docxExercise 3a Flowchart Critique Facilitator Guide.docxExercise 3b Fishbone Critique Facilitator Guide.docxExercise 4 Measures Critique Facilitator Guide.docxExercise 5 Intervention Critique Facilitator Guide.docxTrain-the-Trainer Quality Preassessment.docxCourse Evaluation.docxTrain-the-Trainer Quality Postassessment.doc [file mep_2374-8265.11425-s001.zip › B. Exercise 1 Aim Statements.docx]

**Exercise #1**

**Critiquing an Aim Statement**

**Aim Statement 1:**

- Increase the incidence of flu vaccination within the Family Medicine clinic.

Critique the above aim statement as pertains to inclusion of the SMAART criteria (**S**pecific, **M**easurable, **A**ggressive & yet **A**chievable, **R**elevant, **T**ime-bound):

How would you re-construct the aim statement to add necessary clarity?

**Aim Statement 2:**

- Improve post-operative pain relief while reducing opioid requirements and opioid adverse effects.

Critique the above aim statement as pertains to inclusion of the SMAART criteria (**S**pecific, **M**easurable, **A**ggressive & yet **A**chievable, **R**elevant, **T**ime-bound):

How would you re-construct the aim statement to add necessary clarity?
