# Supplementary material for: Development of a Train-the-Trainer Quality Improvement Curriculum
Source: MedEdPORTAL. 2024 Jul 16;20:11425. doi: 10.15766/mep_2374-8265.11425 (PMC11249715; doi:10.15766/mep_2374-8265.11425)

## Exercise #2: STAKEHOLDER ANALYSIS

*Project background:*  The anesthesia and orthopedic residents, recognizing an opportunity to enhance post-operative recovery with less reliance on opioid therapy, would like to move towards a multimodal analgesia protocol for patients admitted to the hospital in the first 24 hours post-operatively.

*Project Aim Statement:* To reduce 24-hour post-operative opioid requirements by 25% in patients status post hip fracture repair admitted to St. Francis by January 1, 2024.

| **1**  **Stakeholder Group** | **2**  **Opinion Leaders/Key Individuals** | **3**  **How Affected** | **4**  **Criticality to Success (L,H)** | **5**  **Level of Commitment to Change**  **(L,H)** | **6**  **Suggested Way to Involve in Change**  **(Level of involvement)** |
| --- | --- | --- | --- | --- | --- |
| Anesthesia Residents and Faculty | XXXXX | Pain consultation | High | High | Critical Team Member |
| Orthopedic Residents and Faculty | XXXXX | Post-operative care | High | High | Enlist Help |
| Electronic medical record support team | XXXXX | Order Set Build | High | Low | Report Out |

**Evaluate the content**

**of this stakeholder analysis and provide feedback considering the following:**

Are all key stakeholder groups included? Who might be missing?

Is the learner’s assessment of the stakeholder’s criticality to success and commitment to change accurate?

Does the “suggested way to involve in change” match the content indicated in “criticality to success” and “level of commitment to change”?


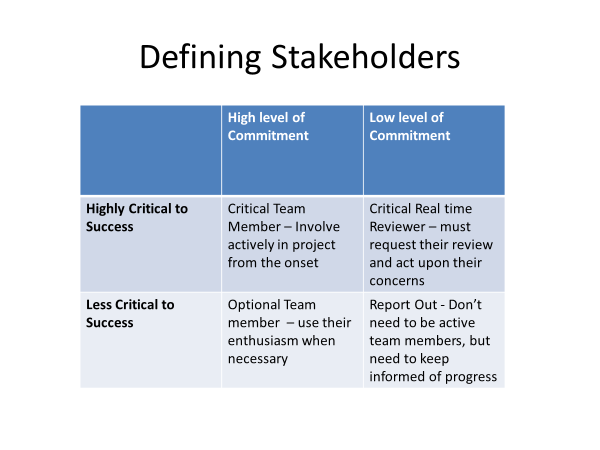

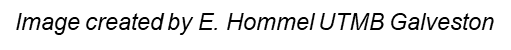

Supplement: Supplementary file 1 — Train-the-Trainer Slide Set.pptxExercise 1 Aim Statements.docxExercise 2 Stakeholder Analysis.docxExercise 3a Flowchart Critique.docxExercise 3b Fishbone Critique.docxExercise 4 Measures Critique.docxExercise 5 Intervention Critique.docxExercise 1 Aim Statements Facilitator Guide.docxExercise 2 Stakeholder Analysis Facilitator Guide.docxExercise 3a Flowchart Critique Facilitator Guide.docxExercise 3b Fishbone Critique Facilitator Guide.docxExercise 4 Measures Critique Facilitator Guide.docxExercise 5 Intervention Critique Facilitator Guide.docxTrain-the-Trainer Quality Preassessment.docxCourse Evaluation.docxTrain-the-Trainer Quality Postassessment.doc [file mep_2374-8265.11425-s001.zip › C. Exercise 2 Stakeholder Analysis.docx]
