# Supplementary material for: Development of a Train-the-Trainer Quality Improvement Curriculum
Source: MedEdPORTAL. 2024 Jul 16;20:11425. doi: 10.15766/mep_2374-8265.11425 (PMC11249715; doi:10.15766/mep_2374-8265.11425)
Supplement: Supplementary file 1 — Train-the-Trainer Slide Set.pptxExercise 1 Aim Statements.docxExercise 2 Stakeholder Analysis.docxExercise 3a Flowchart Critique.docxExercise 3b Fishbone Critique.docxExercise 4 Measures Critique.docxExercise 5 Intervention Critique.docxExercise 1 Aim Statements Facilitator Guide.docxExercise 2 Stakeholder Analysis Facilitator Guide.docxExercise 3a Flowchart Critique Facilitator Guide.docxExercise 3b Fishbone Critique Facilitator Guide.docxExercise 4 Measures Critique Facilitator Guide.docxExercise 5 Intervention Critique Facilitator Guide.docxTrain-the-Trainer Quality Preassessment.docxCourse Evaluation.docxTrain-the-Trainer Quality Postassessment.doc [file mep_2374-8265.11425-s001.zip › D. Exercise 3a Flowchart Critique.docx]

**Exercise #3: Flowchart Review and Critique**

**Problem Statement: Low Influenza Vaccination Rates within the Family Medicine Clinic**

Created by E. Hommel UTMB Galveston

**Flowchart Critique Tool:**

Does the flowchart begin and end with the appropriate steps?

Does the flowchart represent “current state” or “ideal state”? For purposes of background investigation, it should represent the “current state”.

Do the steps identified capture the process in its entirety? Are there missing steps?

Does it appear that the tool was completed with inclusion of the QI team and therefore multiple stakeholders in the process?
