# Supplementary material for: Development of a Train-the-Trainer Quality Improvement Curriculum
Source: MedEdPORTAL. 2024 Jul 16;20:11425. doi: 10.15766/mep_2374-8265.11425 (PMC11249715; doi:10.15766/mep_2374-8265.11425)
Supplement: Supplementary file 1 — Train-the-Trainer Slide Set.pptxExercise 1 Aim Statements.docxExercise 2 Stakeholder Analysis.docxExercise 3a Flowchart Critique.docxExercise 3b Fishbone Critique.docxExercise 4 Measures Critique.docxExercise 5 Intervention Critique.docxExercise 1 Aim Statements Facilitator Guide.docxExercise 2 Stakeholder Analysis Facilitator Guide.docxExercise 3a Flowchart Critique Facilitator Guide.docxExercise 3b Fishbone Critique Facilitator Guide.docxExercise 4 Measures Critique Facilitator Guide.docxExercise 5 Intervention Critique Facilitator Guide.docxTrain-the-Trainer Quality Preassessment.docxCourse Evaluation.docxTrain-the-Trainer Quality Postassessment.doc [file mep_2374-8265.11425-s001.zip › F. Exercise 4 Measures Critique.docx]

# *Exercise #4: Critiquing Proposed Measures*

| **Project Title:** Reducing Kidney Injury Associated with Combination Vancomycin and Piperacillin/Tazobactam Use |
| --- |
| **Problem Statement (general problem background)** |
| As part of the antibiotic stewardship program, we would like to help the health system reduce inappropriate use of combination vancomycin and piperacillin-tazobactam (broad spectrum antibiotics) as this combination can lead to acute kidney injury. |
| **Aim Statement (specific goal of project)** |
| To decrease the incidence of nephrotoxicity (increases in serum creatinine; acute kidney injury) induced with combination vancomycin/piperacillin-tazobactam by reducing the concurrent use of these antibiotics in geriatric patients admitted to hospital unit 11D by 15% by 05/2024. |
| **Proposed Interventions** |
| - Educate the geriatric fellows and internal medicine residents on the harms of concurrent vancomycin & piperacillin/tazobactam - Create a pocket card to remind them of alternative antibiotic choices specific for certain infections - Provide monthly feedback on how often patients received combination vancomycin & piperacillin/tazobactam |
| **Performance Measures** |
| **Main Measure(s): Outcomes or Process Measures**  Total number of patients on 11D who receive combination vancomycin & piperacillin/tazobactam.  Total number of residents who receive the educational pocket card. |
| **Balancing Measure(s)**  Total number of patients who receive expensive Carbapenems on 11D |

**Appraise the above project proposal as it pertains to the suggested measures.**

Which measure is an outcome measure? Which measure is a process measure?

Are there additional or alternative measures you would suggest?

Is the balancing measure appropriate?

Are the suggested measures well defined? Could you reproduce the numerator/denominator?

Where are the learners likely to obtain the requested data? How would you recommend they display their data?
