# Supplementary material for: Development of a Train-the-Trainer Quality Improvement Curriculum
Source: MedEdPORTAL. 2024 Jul 16;20:11425. doi: 10.15766/mep_2374-8265.11425 (PMC11249715; doi:10.15766/mep_2374-8265.11425)
Supplement: Supplementary file 1 — Train-the-Trainer Slide Set.pptxExercise 1 Aim Statements.docxExercise 2 Stakeholder Analysis.docxExercise 3a Flowchart Critique.docxExercise 3b Fishbone Critique.docxExercise 4 Measures Critique.docxExercise 5 Intervention Critique.docxExercise 1 Aim Statements Facilitator Guide.docxExercise 2 Stakeholder Analysis Facilitator Guide.docxExercise 3a Flowchart Critique Facilitator Guide.docxExercise 3b Fishbone Critique Facilitator Guide.docxExercise 4 Measures Critique Facilitator Guide.docxExercise 5 Intervention Critique Facilitator Guide.docxTrain-the-Trainer Quality Preassessment.docxCourse Evaluation.docxTrain-the-Trainer Quality Postassessment.doc [file mep_2374-8265.11425-s001.zip › G. Exercise 5 Intervention Critique.docx]

# *Exercise #5: Critiquing Proposed Interventions*

| **Project Title:** Reducing Nephrotoxicity Associated with Combination Vancomycin and Piperacillin/Tazobactam Use |
| --- |
| **Problem Statement (general problem background)** |
| As part of the antibiotic stewardship program, we would like to help the health system reduce inappropriate use of combination vancomycin and piperacillin-tazobactam (broad spectrum antibiotics) as this combination can lead to acute kidney injury. |
| **Aim Statement (specific goal of project)** |
| To decrease the incidence of nephrotoxicity (increases in serum creatinine; acute kidney injury) induced with combination vancomycin/piperacillin-tazobactam by reducing the concurrent use of these antibiotics in geriatric patients admitted to hospital unit 11D by 15% by 05/2024. |
| **Proposed Interventions** |
| - Educate the geriatric fellows and internal medicine residents on the risk of concurrent vancomycin & piperacillin/tazobactam - Create a pocket card to remind them of alternative antibiotic choices specific for certain infections - Provide monthly feedback on how often patients received combination vancomycin & piperacillin/tazobactam |

**Consider the suggested interventions.**

**What do you think the effectiveness will be of each intervention?**

**How would you encourage a learner to prioritize these interventions?**

**Can you recommend additional interventions that might lead the project to greater likelihood of success?**

**Consider the technical & social aspects of change that this project requires. What driving or restraining forces can you perceive the students will encounter? How would you help your learners organize their improvement efforts to overcome these barriers?**
