# Supplementary material for: Development of a Train-the-Trainer Quality Improvement Curriculum
Source: MedEdPORTAL. 2024 Jul 16;20:11425. doi: 10.15766/mep_2374-8265.11425 (PMC11249715; doi:10.15766/mep_2374-8265.11425)
Supplement: Supplementary file 1 — Train-the-Trainer Slide Set.pptxExercise 1 Aim Statements.docxExercise 2 Stakeholder Analysis.docxExercise 3a Flowchart Critique.docxExercise 3b Fishbone Critique.docxExercise 4 Measures Critique.docxExercise 5 Intervention Critique.docxExercise 1 Aim Statements Facilitator Guide.docxExercise 2 Stakeholder Analysis Facilitator Guide.docxExercise 3a Flowchart Critique Facilitator Guide.docxExercise 3b Fishbone Critique Facilitator Guide.docxExercise 4 Measures Critique Facilitator Guide.docxExercise 5 Intervention Critique Facilitator Guide.docxTrain-the-Trainer Quality Preassessment.docxCourse Evaluation.docxTrain-the-Trainer Quality Postassessment.doc [file mep_2374-8265.11425-s001.zip › H. Exercise 1 Aim Statements Facilitator Guide.docx]

**Exercise #1 Facilitators Guide**

**Critiquing an Aim Statement**

For the group exercises to be most effective, it is best to arrange participants into groups of 3-4 people. Provide copies of the worksheet to each group. Ask for each group to appoint a spokesperson for the large group debrief. Instructions for the debrief are included in the PowerPoint slides.

Suggested time allotment for this exercise: 10 minutes (5-7 minutes for group work; 3 minutes for debrief)

This exercise should be used alongside the train the trainer slide set, although could individually be used to teach aim statement formation.

**Aim Statement 1:**

- Increase the incidence of flu vaccination within the Family Medicine clinic

Critique the above aim statement as pertains to inclusion of the SMAART criteria (**S**pecific, **M**easurable, **A**ggressive & yet **A**chievable, **R**elevant, **T**ime-bound):

When we use the SMAART criteria, this aim statement is missing a measurement and a time-bound statement. The is no mention of how much we want to increase the flu vaccination, nor is there an end date included. For the measurement, learners should consider national benchmarks. How many patients should be receiving the flu vaccination? (For reference, Healthy People 2030 indicates a target of 70% with approximately 50% of persons currently vaccinated.) Learners can assume a current performance level or be provided with data of institutional performance.

Authors believe that a measure can be written as “increase X to Y” or can be written as “increase by Z%”. Also, time can be included by giving a specific end date or a time frame. For instance, one could write “by 6/1/2023” or “within 6 months”. Both the measurement and the time period should be critiqued as “aggressive but achievable”.

Additionally depending on the clinical area, we may need to add more specificity to “Family Medicine clinic” - is there more than 1 family medicine clinic? If so, does this project apply to all of them or just certain ones? (For instance, are you targeting resident only clinics or faculty only? Or a specific location?)

How would you re-construct the aim statement to add necessary clarity?

We AIM to increase flu vaccinations in the x Family Medicine clinic from 50% to 70% within 4 months. It is noted that this statement could be rewritten in a variety of ways, but the underlined portions are what need to be included added for a more comprehensive aim statement.

**Aim Statement 2:**

- Improve post-operative pain relief while reducing opioid requirements and opioid adverse effects.

Critique the above aim statement as pertains to inclusion of the SMAART criteria (**S**pecific, **M**easurable, **A**ggressive & yet **A**chievable, **R**elevant, **T**ime-bound):

When we use the SMAART criteria, this aim statement has issues with all the criteria. The first thing that needs to be established is: what is the problem statement? For example, is the problem statement inadequate control of post-operative pain, too high opioid requirements or too many opioid adverse effects? Once this has been determined the statement could be written to fulfil the SMAART criteria.

How would you re-construct the aim statement to add necessary clarity?

Here are 3 different aim statements depending on what the problem statement is:

1. We AIM to reduce post operative pain (as measured by the 0-10 pain scale) in lap chole patients (example population) at x hospital by 20% within 6 months.
2. We AIM to reduce post operative opioid requirements in x patient population at x hospital by 20% within 6 months.
3. We AIM to reduce the percent of patients reporting delirium as an opioid adverse effect in x patient population at x hospital by 20% within 6 months.

The point of this aim statement is for the group to find what is relevant. They then can rewrite the aim statement several ways to add specificity, measurement, and make it time-bound. You may need to further add discussion about how to benchmark their goal measure and establish what is aggressive yet achievable.
