# Supplementary material for: Development of a Train-the-Trainer Quality Improvement Curriculum
Source: MedEdPORTAL. 2024 Jul 16;20:11425. doi: 10.15766/mep_2374-8265.11425 (PMC11249715; doi:10.15766/mep_2374-8265.11425)

## Exercise #2: STAKEHOLDER ANALYSIS FACILITATOR GUIDE

This exercise should take approximately 5-8 minutes total. Please allow your groups to spend up to 5 minutes answering the questions at the bottom of the page. You can ask some groups to start with the bottom questions and others to answer the top questions to speed up the exercise. Allow debrief time of 3 minutes.

*Project background:*  The anesthesia and orthopedic residents, recognizing an opportunity to enhance post-operative recovery with less reliance on opioid therapy, would like to move towards a multimodal analgesia protocol for patients admitted to the hospital in the first 24 hours post-operatively.

*Project Aim Statement:* To reduce 24-hour post-operative opioid requirements by 25% in patients status post hip fracture repair admitted to St. Francis by January 1, 2024.

| **1**  **Stakeholder Group** | **2**  **Opinion Leaders/Key Individuals** | **3**  **How Affected** | **4**  **Criticality to Success (L,H)** | **5**  **Level of Commitment to Change**  **(L,H)** | **6**  **Suggested Way to Involve in Change**  **(Level of involvement)** |
| --- | --- | --- | --- | --- | --- |
| Anesthesia Residents and Faculty | XXXXX | Pain consultation | High | High | Critical Team Member |
| Orthopedic Residents and Faculty | XXXXX | Post-operative care | High | High | Enlist Help |
| Electronic medical record support team | XXXXX | Order Set Build | High | Low | Report Out |

**Evaluate this stakeholder analysis and provide feedback on the following:**

Are all key stakeholder groups included? Who might be missing?

Stakeholder groups may differ for each institution but here are some that are potentially missing:

-Patients/family members - these are key stakeholders

-Pharmacy -particularly if there are restrictions to pain regimens in your hospital

-Other hospitalist teams (Are they cared for by orthopedics or are they on a geriatrics, internal medicine, or family medicine service?)

-Nursing –they certainly have a role in assessing pain scores and administering prn medications. You would definitely want to get their engagement, especially if you are introducing new pain assessments or protocols.

-This project may also consider separating orthopedics and anesthesia residents from faculty as the level of commitment to change and criticality to success may differ for trainees and faculty.

Is the assessment of the stakeholder’s criticality to success and commitment to change accurate?

This is a subjective rating, but the facilitator should encourage discussion to start to recognize what barriers might arise from stakeholder groups. Sometimes, it takes talking to the stakeholder groups to identify their level of commitment. Failure to engage the right stakeholders in the right way at the right time will create trouble as the team moves ahead.

Does the “suggested way to involve in change” match the content indicated in “criticality to success” and “level of commitment to change”?

-If orthopedics residents and faculty are considered highly critical to success and highly committed to change they should all be critical team members.

-The EMR support team should be considered a critical real time reviewer.

-You want to match the level of commitment and the level of criticality to the quadrant for engagement as indicated in the table below.


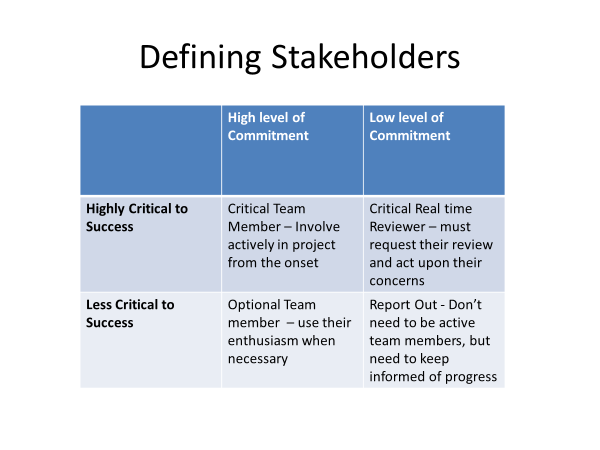


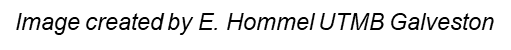

Supplement: Supplementary file 1 — Train-the-Trainer Slide Set.pptxExercise 1 Aim Statements.docxExercise 2 Stakeholder Analysis.docxExercise 3a Flowchart Critique.docxExercise 3b Fishbone Critique.docxExercise 4 Measures Critique.docxExercise 5 Intervention Critique.docxExercise 1 Aim Statements Facilitator Guide.docxExercise 2 Stakeholder Analysis Facilitator Guide.docxExercise 3a Flowchart Critique Facilitator Guide.docxExercise 3b Fishbone Critique Facilitator Guide.docxExercise 4 Measures Critique Facilitator Guide.docxExercise 5 Intervention Critique Facilitator Guide.docxTrain-the-Trainer Quality Preassessment.docxCourse Evaluation.docxTrain-the-Trainer Quality Postassessment.doc [file mep_2374-8265.11425-s001.zip › I. Exercise 2 Stakeholder Analysis Facilitator Guide.docx]
