# Supplementary material for: Development of a Train-the-Trainer Quality Improvement Curriculum
Source: MedEdPORTAL. 2024 Jul 16;20:11425. doi: 10.15766/mep_2374-8265.11425 (PMC11249715; doi:10.15766/mep_2374-8265.11425)
Supplement: Supplementary file 1 — Train-the-Trainer Slide Set.pptxExercise 1 Aim Statements.docxExercise 2 Stakeholder Analysis.docxExercise 3a Flowchart Critique.docxExercise 3b Fishbone Critique.docxExercise 4 Measures Critique.docxExercise 5 Intervention Critique.docxExercise 1 Aim Statements Facilitator Guide.docxExercise 2 Stakeholder Analysis Facilitator Guide.docxExercise 3a Flowchart Critique Facilitator Guide.docxExercise 3b Fishbone Critique Facilitator Guide.docxExercise 4 Measures Critique Facilitator Guide.docxExercise 5 Intervention Critique Facilitator Guide.docxTrain-the-Trainer Quality Preassessment.docxCourse Evaluation.docxTrain-the-Trainer Quality Postassessment.doc [file mep_2374-8265.11425-s001.zip › J. Exercise 3a Flowchart Critique Facilitator Guide.docx]

**Exercise #3a Flow chart critique – Facilitator Guide**

Time needed: 15 minutes; 7-10 minutes for group work / 5-7 minutes for debrief

For this exercise we suggest dividing the room in half. Give half the participants the flow chart exercise (labeled exercise #3A) and half the participants the fishbone exercise (labeled exercise #3B). This allows an efficient way to critique both tools at the same time. Give each group approximately 7-10 minutes for this exercise and choose 2 groups (1 flow chart and 1 fishbone) to report out.

**Problem Statement: Low Influenza Vaccination Rates within the Family Medicine Clinic**


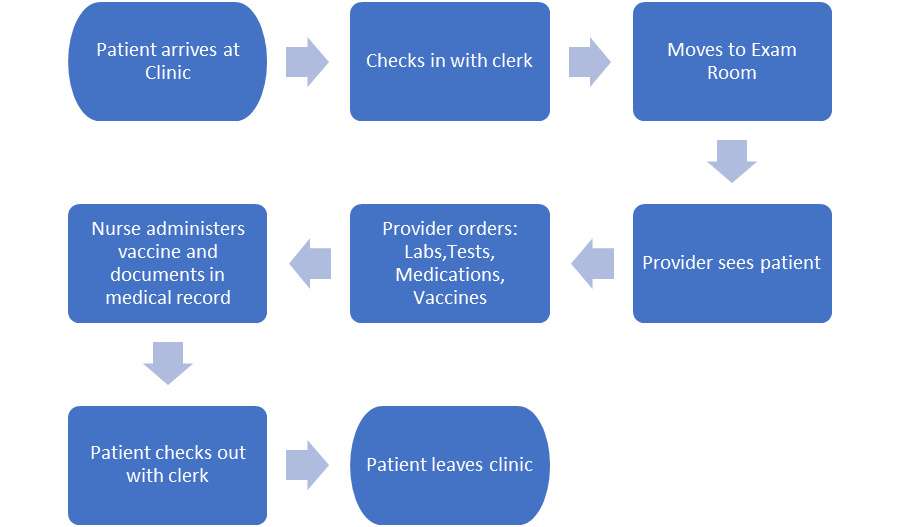


Created by E. Hommel UTMB Galveston

**Flowchart Critique Tool:**

Does the flowchart begin and end with the appropriate steps?

The hardest part of developing a flow chart is the start and end steps. In this specific example the start and end steps are correct.

Does the flowchart represent “current state” or “ideal state”? For purposes of background investigation, it should represent the “current state”.

It appears that this flow chart is documenting the ideal state and not the current state.

Do the steps identified capture the process in its entirety? Are there missing steps?

It appears there are several steps missing between when the patient is checked in and when the patient moves to the exam room. Are vital signs taken? Are there other questions that are asked to the patients during this step? Is there paperwork that a patient completes?

The second row in the flow chart is likely missing several steps. We would suggest that the learner expands the steps specifically focusing on process to document flu vaccination. (For example: When do you ask the patient about the flu shot? How is it determined to give the flu shot? Does the provider review anything specific in the EMR? What steps must the provider take to order the vaccine or communicate the need for the vaccine to the staff? What happens if the patient received the vaccine at another site outside of the institution?)

Does it appear that the tool was completed with inclusion of the QI team and therefore multiple stakeholders in the process?

It appears that this was made from a physician perspective and did not include the entire interdisciplinary team.
