# Supplementary material for: Development of a Train-the-Trainer Quality Improvement Curriculum
Source: MedEdPORTAL. 2024 Jul 16;20:11425. doi: 10.15766/mep_2374-8265.11425 (PMC11249715; doi:10.15766/mep_2374-8265.11425)
Supplement: Supplementary file 1 — Train-the-Trainer Slide Set.pptxExercise 1 Aim Statements.docxExercise 2 Stakeholder Analysis.docxExercise 3a Flowchart Critique.docxExercise 3b Fishbone Critique.docxExercise 4 Measures Critique.docxExercise 5 Intervention Critique.docxExercise 1 Aim Statements Facilitator Guide.docxExercise 2 Stakeholder Analysis Facilitator Guide.docxExercise 3a Flowchart Critique Facilitator Guide.docxExercise 3b Fishbone Critique Facilitator Guide.docxExercise 4 Measures Critique Facilitator Guide.docxExercise 5 Intervention Critique Facilitator Guide.docxTrain-the-Trainer Quality Preassessment.docxCourse Evaluation.docxTrain-the-Trainer Quality Postassessment.doc [file mep_2374-8265.11425-s001.zip › K. Exercise 3b Fishbone Critique Facilitator Guide.docx]

**Exercise #3b: Fishbone Critique Tool**

Time needed: 15 minutes; 7-10 minutes for group work / 5-7 minutes for debrief

For this exercise we suggest dividing the room in half. Give half the participants the flow chart exercise (labeled exercise #3A) and half the participants the fishbone exercise (labeled exercise #3B). This allows an efficient way to critique both tools at the same time. Give each group approximately 7-10 minutes for this exercise and choose 2 groups (1 flow chart and 1 fishbone) to report out.


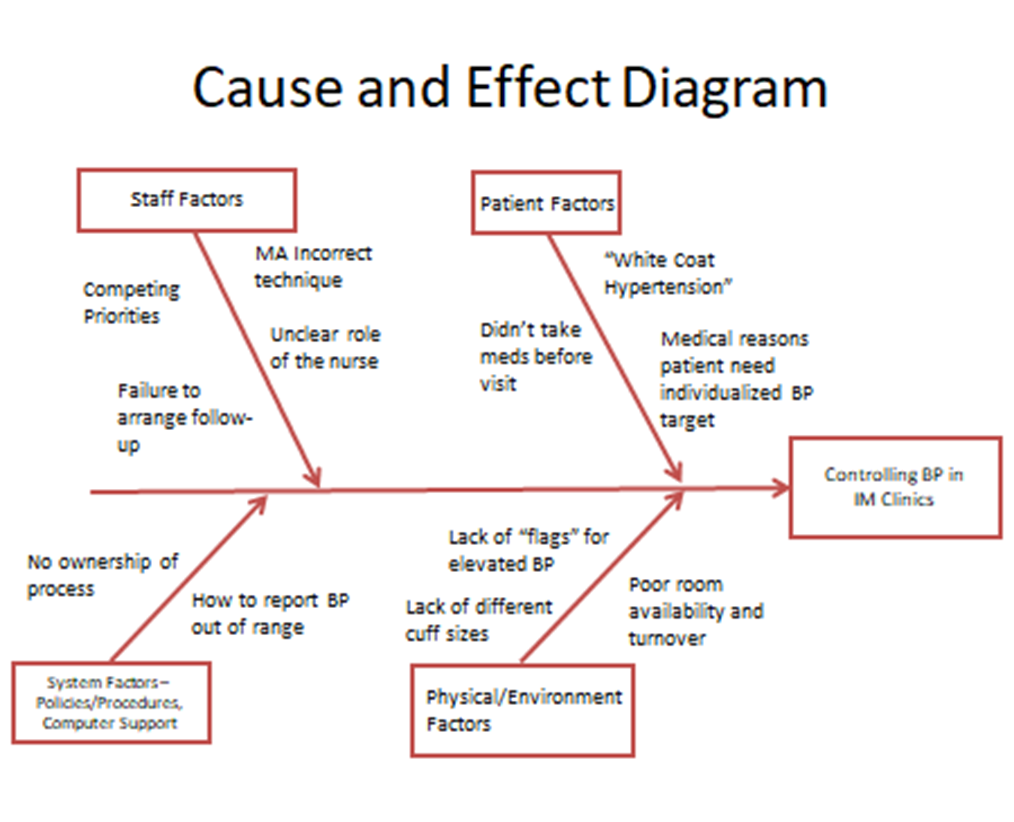


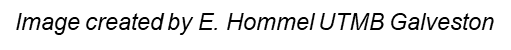


Does the “head” of the “fish” properly represent the PROBLEM statement?

NO, The problem in this example is uncontrolled blood pressure which is not correctly notated in the head of the fish. An appropriate problem statement might be “uncontrolled blood pressure within the resident IM clinics”.

Are the barriers identified properly sub-grouped into meaningful categories?

These categories are generally appropriate and are commonly used for cause and effect diagrams. If a group finds they have a lot of items in the staff factors category, this could be sub-divided into distinct types of personnel (i.e. physicians, ancillary staff, nursing, etc.).

Do the barriers identified capture most of the perceived barriers and appear “complete”?

It seems many of the barriers could be expanded. Some examples of things that are missing include:

Patient: non-compliance, could not afford medications, health literacy, dietary non-compliance

Staff Factors: medical knowledge about blood pressure targets, knowledge of how to adjust the medication regimen

System Factors: no EMR alerts for high blood pressure, access/availability of follow up visits

Does it appear that the tool was completed with inclusion of the QI team and therefore multiple stakeholders in the problem?

It appears this fish bone may not have included physicians as most items are related to clinic rooming staff.
