# Supplementary material for: Development of a Train-the-Trainer Quality Improvement Curriculum
Source: MedEdPORTAL. 2024 Jul 16;20:11425. doi: 10.15766/mep_2374-8265.11425 (PMC11249715; doi:10.15766/mep_2374-8265.11425)
Supplement: Supplementary file 1 — Train-the-Trainer Slide Set.pptxExercise 1 Aim Statements.docxExercise 2 Stakeholder Analysis.docxExercise 3a Flowchart Critique.docxExercise 3b Fishbone Critique.docxExercise 4 Measures Critique.docxExercise 5 Intervention Critique.docxExercise 1 Aim Statements Facilitator Guide.docxExercise 2 Stakeholder Analysis Facilitator Guide.docxExercise 3a Flowchart Critique Facilitator Guide.docxExercise 3b Fishbone Critique Facilitator Guide.docxExercise 4 Measures Critique Facilitator Guide.docxExercise 5 Intervention Critique Facilitator Guide.docxTrain-the-Trainer Quality Preassessment.docxCourse Evaluation.docxTrain-the-Trainer Quality Postassessment.doc [file mep_2374-8265.11425-s001.zip › L. Exercise 4 Measures Critique Facilitator Guide.docx]

# *Exercise #4 Critiquing Proposed Measures*

# Please allow 10 minutes for this exercise (5 minutes for group work and 5 minutes for debrief). Assign each group a question to report out on so they will be prepared to present.

| **Project Title:** Reducing Kidney Injury Associated with Combination Vancomycin and Piperacillin/Tazobactam Use |
| --- |
| **Problem Statement (general problem background)** |
| As part of the antibiotic stewardship program, we would like to help the health system reduce inappropriate use of combination vancomycin and piperacillin-tazobactam (broad spectrum antibiotics) as this combination can lead to acute kidney injury. |
| **Aim Statement (specific goal of project)** |
| To decrease the incidence of nephrotoxicity (increases in serum creatinine; acute kidney injury) induced with combination vancomycin/piperacillin-tazobactam by reducing the concurrent use of these antibiotics in geriatric patients admitted to hospital unit 11D by 15% by 05/2024. |
| **Proposed Interventions** |
| - Educate the geriatric fellows and internal medicine residents on the harms of concurrent vancomycin & piperacillin/tazobactam - Create a pocket card to remind them of alternative antibiotic choices specific for certain infections - Provide monthly feedback on how often patients received combination vancomycin & piperacillin/tazobactam |
| **Performance Measures** |
| **Main Measure(s): Outcomes or Process Measures**  Total number of patients on 11D who receive combination vancomycin & piperacillin/tazobactam.  Total number of residents who receive the educational pocket card. |
| **Balancing Measure(s)**  Total number of patients who receive expensive Carbapenems on 11D |

**Appraise the above project proposal as pertains to the suggested measures.**

Which measure is an outcome measure? The number of patients who receive combination antibiotic therapy

Which measure is a process measure? The process measure is the number of residents who receive the educational pocket card.

Are there additional or alternative measures you would suggest? An additional outcome measure is the number of patients who develop nephrotoxocity.

Is the balancing measure appropriate? This is not the only balancing measure. Better balancing metrics for this project might include mortality from sepsis or the number of patients who did not receive guideline based antibiotic therapy.

Are the suggested measures well defined? Could you reproduce the numerator/denominator?

- Number of patients receiving combination therapy is well defined and likely easy to reproduce. The denominator will be time (i.e., number of patients per week or per month).
- For the process measure, the learners would need to develop a system as part of the intervention for recording who receives the educational pocket card.
- The suggested additional outcome measure of nephrotoxicity would be somewhat harder to define. The learner would need to define nephrotoxicity and determine a way to abstract this from the EHR.

Where are the learners likely to obtain the requested data? How would you recommend they display their data?

Requesting this data will differ per institution. (Can this come from the electronic health record? Is there an existing pharmacy report?) We recommend each presenter be prepared to address this during the debrief.

This data is best presented over time in a run chart. The learner could present this data weekly or monthly. The ultimate outcome might be to create a statistical process control chart for scholarly presentation.
