# Supplementary material for: Development of a Train-the-Trainer Quality Improvement Curriculum
Source: MedEdPORTAL. 2024 Jul 16;20:11425. doi: 10.15766/mep_2374-8265.11425 (PMC11249715; doi:10.15766/mep_2374-8265.11425)
Supplement: Supplementary file 1 — Train-the-Trainer Slide Set.pptxExercise 1 Aim Statements.docxExercise 2 Stakeholder Analysis.docxExercise 3a Flowchart Critique.docxExercise 3b Fishbone Critique.docxExercise 4 Measures Critique.docxExercise 5 Intervention Critique.docxExercise 1 Aim Statements Facilitator Guide.docxExercise 2 Stakeholder Analysis Facilitator Guide.docxExercise 3a Flowchart Critique Facilitator Guide.docxExercise 3b Fishbone Critique Facilitator Guide.docxExercise 4 Measures Critique Facilitator Guide.docxExercise 5 Intervention Critique Facilitator Guide.docxTrain-the-Trainer Quality Preassessment.docxCourse Evaluation.docxTrain-the-Trainer Quality Postassessment.doc [file mep_2374-8265.11425-s001.zip › M. Exercise 5 Intervention Critique Facilitator Guide.docx]

# *Exercise #5: Critiquing Proposed Interventions*

# Allow 7 minutes for this group work (5 minutes for group work, 2 minutes for debrief). Ask each group to report back on a different question so you have time to review all aspects of the analysis.

| **Project Title:** Reducing Nephrotoxicity Associated with Combination Vancomycin and Piperacillin/Tazobactam Use |
| --- |
| **Problem Statement (general problem background)** |
| As part of the antibiotic stewardship program, we would like to help the health system reduce inappropriate use of combination vancomycin and piperacillin-tazobactam (broad spectrum antibiotics) as this combination can lead to acute kidney injury. |
| **Aim Statement (specific goal of project)** |
| To decrease the incidence of nephrotoxicity (increases in serum creatinine; acute kidney injury) induced with combination vancomycin/piperacillin-tazobactam by reducing the concurrent use of these antibiotics in geriatric patients admitted to hospital unit 11D by 15% by 05/2024. |
| **Proposed Interventions** |
| - Educate the geriatric fellows and internal medicine residents on the risk of concurrent vancomycin & piperacillin/tazobactam - Create a pocket card to remind them of alternative antibiotic choices specific for certain infections - Provide monthly feedback on how often patients received combination vancomycin & piperacillin/tazobactam |

**Consider the suggested interventions.**

**What do you think the effectiveness will be of each intervention?**

The educational session is necessary for the success of the project but will likely be the least successful intervention as educational interventions tend to be short lived and only attended by small numbers of trainees.

The pocket card will be somewhat more effective, as it will serve as a reminder tool and educate those who did not attend the educational session.

Monthly feedback will likely be the most successful of these interventions in changing personal practice.

**How would you encourage a learner to prioritize these interventions?**

Learners should prioritize based on buy in and resources. Do the learners have the ability/ bandwidth to develop a monthly feedback process? You cannot start a project without “training the ranks” so the educational pieces should be completed initially.

**Can you recommend additional interventions that might lead the project to greater likelihood of success?**

Electronic health record (EHR) changes such as order sets, warnings or even hard stops would likely lead to greater systemic change.

Institutional protocols such as antibiotic stewardship programs could also lead to more effective systemic change.

**Consider the technical & social aspects of change that this project requires. What driving or restraining forces can you perceive the students will encounter? How would you help your learners organize their improvement efforts to overcome these barriers?**

Driving forces that learners may see are:

Institutional or pharmacy buy in

Cost Savings

Length of stay improvements

Motivation to improve patient care

Restraining forces learners may see are:

Social change – physicians being unwilling to change their current practice

Pharmacy restrictions to alternative antibiotic choices

An additional EHR warning/flag or hard stop (alert fatigue)

Leaners need to balance these things when determining their intervention. If there are already a lot of EHR hard stops, maybe choose a pharmacy driven protocol instead of an EHR warning. If there is a lack of resources to build the EHR change, the first PDSA cycle may have to focus on education and building buy-in. Learners can also overcome practice barriers by engaging Infectious disease specialists or presenting data related to guideline-based care. Help the learner to consider creative ways to build strength for driving forces and minimize restraining forces.
