# Supplementary material for: Development of a Train-the-Trainer Quality Improvement Curriculum
Source: MedEdPORTAL. 2024 Jul 16;20:11425. doi: 10.15766/mep_2374-8265.11425 (PMC11249715; doi:10.15766/mep_2374-8265.11425)
Supplement: Supplementary file 1 — Train-the-Trainer Slide Set.pptxExercise 1 Aim Statements.docxExercise 2 Stakeholder Analysis.docxExercise 3a Flowchart Critique.docxExercise 3b Fishbone Critique.docxExercise 4 Measures Critique.docxExercise 5 Intervention Critique.docxExercise 1 Aim Statements Facilitator Guide.docxExercise 2 Stakeholder Analysis Facilitator Guide.docxExercise 3a Flowchart Critique Facilitator Guide.docxExercise 3b Fishbone Critique Facilitator Guide.docxExercise 4 Measures Critique Facilitator Guide.docxExercise 5 Intervention Critique Facilitator Guide.docxTrain-the-Trainer Quality Preassessment.docxCourse Evaluation.docxTrain-the-Trainer Quality Postassessment.doc [file mep_2374-8265.11425-s001.zip › N. Train-the-Trainer Quality Preassessment.docx]

Train the Trainer Quality PreAssessment

Demographic Information:

1. What is your profession? (circle all that apply)
2. Physician
3. Allied Health
4. Pharmacy
5. Nursing
6. Administration
7. Other (please state) _______________
8. How many years of QI educational experience do you have?
9. None
10. 1-5 years
11. 6-10 years
12. >10 years
13. Do you have any formal QI training? (circle) YES NO
14. Do you have any formal Educational training? (circle) YES NO

Knowledge Test: (highlight is the correct answer)

1. All of the following aim statements are appropriate EXCEPT ?
2. Reduce waiting time to see a physician in the Internal Medicine Residents’ Clinic to less than 15 minutes within 6 months.
3. Improve glucose control in patients in the Internal Medicine Clinic within 9 months.
4. Transfer 70% of patients admitted to the general surgery service from the Emergency Department to an inpatient room within one hour from admission over the next 6 months.
5. Decrease percentage of Adverse Drug Events in the Surgical Intensive Care Unit by 75% within the next 6 months.
6. What are the components of quality improvement?
7. Analysis of performance with systematic improvements to improve the performance
8. Use of methods to assure that a service or product confines to a desired standard
9. Compare one’s own performance to an external standard established as high performance
10. Process of assuring that a service or product meets a predefined set of criteria
11. Which of the following is NOT a Model for Improvement?
12. PDSA
13. Lean
14. Six Sigma
15. Root Cause Analysis
16. Which Quality Improvement tool helps to clarify an Understanding of the Current Process?
17. Ishikawa Diagram
18. Stake holder Analysis
19. Force field analysis
20. Run Chart
21. None of the above
22. You are doing a QI project on improvement of flu vaccination rates in your clinic. Your intervention is to have the medical assistant do an assessment of flu vaccine status and if the patient has not received the vaccine, they will educate the patient on the vaccine. Which of the following is a balancing metric you will need to follow during this project?
23. Flu Vaccination Rate
24. Rooming times
25. Number of flu assessments performed
26. None of the above
27. Using the same flu vaccination quality improvement project above, which quality improvement intervention is likely to be LEAST effective?
28. Creating a new policy that mandates the new flu assessment
29. Educating the staff about the new flu assessment and education process
30. Creating the flu assessment in the electronic health record with a hard stop ensuring completion
31. Creating a paper flu assessment for the staff to complete with each patient
32. Which of the following is NOT a reason quality improvement projects fail?
33. Changing things rapidly
34. Managing the social change
35. Under-training the multidisciplinary team
36. Expecting your implementation to last without iterations of the project

For the following questions, rate your confidence in the following items related to teaching quality improvement:

Confidence Level: 1 = Not Confident 2 = Some Confidence 3 = Very Confident

| 1. Building effective QI didactic materials for your learners | 1 | 2 | 3 |
| --- | --- | --- | --- |
| 1. Leading trainees in experiential QI learning including project development, facilitation, and scholarly presentations | 1 | 2 | 3 |
| 1. Appraising a QI proposal as regards to project aim, measures, chosen intervention and implementation strategies | 1 | 2 | 3 |
