# Supplementary material for: Development of a Train-the-Trainer Quality Improvement Curriculum
Source: MedEdPORTAL. 2024 Jul 16;20:11425. doi: 10.15766/mep_2374-8265.11425 (PMC11249715; doi:10.15766/mep_2374-8265.11425)
Supplement: Supplementary file 1 — Train-the-Trainer Slide Set.pptxExercise 1 Aim Statements.docxExercise 2 Stakeholder Analysis.docxExercise 3a Flowchart Critique.docxExercise 3b Fishbone Critique.docxExercise 4 Measures Critique.docxExercise 5 Intervention Critique.docxExercise 1 Aim Statements Facilitator Guide.docxExercise 2 Stakeholder Analysis Facilitator Guide.docxExercise 3a Flowchart Critique Facilitator Guide.docxExercise 3b Fishbone Critique Facilitator Guide.docxExercise 4 Measures Critique Facilitator Guide.docxExercise 5 Intervention Critique Facilitator Guide.docxTrain-the-Trainer Quality Preassessment.docxCourse Evaluation.docxTrain-the-Trainer Quality Postassessment.doc [file mep_2374-8265.11425-s001.zip › _Educational Summary Report_11425.pdf]

MedEdPORTAL® The AAMC Journal of Teaching and Learning Resources

Original Publication Open Access

Development of a Train-the-Trainer Quality Improvement Curriculum

Lindsay Sonstein, MD*, Erin Hommel, MD

*Corresponding author: lksonste@utmb.edu

Abstract

Introduction: Quality improvement (QI) curricula are required for clinical disciplines at all training levels. Despite this, faculty educators often feel inadequately prepared to perform QI functions and thus lack the skills necessary to teach QI to learners. We aimed to improve faculty QI skills so they could oversee didactic curricula and experiential QI projects. **Methods:** We developed a train-the-trainer curriculum for faculty within medicine, nursing, and allied health that was delivered as a 2-hour interactive workshop. Core concepts included QI methodologies, measurement, implementation, and scholarship. Prior to the workshop, attendees completed a baseline knowledge test and a self-assessment of their confidence in teaching QI. Both assessments were repeated 1 month and 6 months postworkshop. Participants also completed a course evaluation. **Results:** We report on our experience after two workshops with 23 participants total. Baseline median knowledge test percentage correct was 36%. This increased to 77% at 1 month and remained at 57% at 6 months. Self-assessment ratings of QI teaching skills increased consistently from baseline to 1 month to 6 months, with all respondents reporting feeling some confidence or very confident by the end of the study period. The course overall was rated very good or excellent by 91% of attendees. **Discussion:** A focused QI train-the-trainer curriculum can sustainably improve faculty knowledge and self-ratings of QI teaching skills. Participants rated the interactive 2-hour workshop highly. Its materials can be easily adapted across disciplines and clinical departments to increase the number of faculty competent to facilitate didactic and experiential QI training.

Keywords
Train-the-Trainer, Curriculum Development, Faculty Development, Interprofessional Education, Quality Improvement/Patient Safety

Educational Objectives

By the end of this activity, participants will be able to:

1. Build upon their existing quality improvement (QI) background to solidify knowledge of QI principles.
2. Demonstrate the ability to critically appraise a trainee's QI proposal, including project stakeholders, aims, measures, and interventions.
3. Adapt course materials into a manageable didactic or experiential QI curriculum specific to their learners' discipline or clinical specialty.

Introduction

Didactic and experiential quality improvement (QI) curricula are expected across clinical disciplines at most training levels.[1-4]

Citation:
Sonstein L, Hommel E. Development of a train-the-trainer quality improvement curriculum. *MedEdPORTAL*. 2024;20:11425.
https://doi.org/10.15766/mep_2374-8265.11425

A key factor in the success of these curricula is having QI curricular leaders and QI-trained project facilitators.[5,6] Many institutions struggle to identify faculty with adequate QI expertise or even prior QI experience.[7] Training faculty mentors specific to a clinical discipline or medical specialty is an important tactic to enhance didactic and experiential QI training.[8]

Many strategies exist for enhancing faculty competency in QI. Several national organizations have developed in-person or virtual QI faculty training programs.[9,10] Additionally, resource-rich academic institutions have successfully used local quality academies to train faculty.[11] Each of these strategies may be cost prohibitive and/or time intensive. Strategies are more limited for resource-poor environments.

Using a train-the-trainer model can permit an organization to equip faculty easily and rapidly for QI education. Train-the-trainer is an educational model where a content expert identifies trainers with ties to the learners targeted for training.[12] These trainers are provided with education, instructional tools, and guidelines to enable them to offer specific training to their target audience. This model has several advantages. While initial development

Copyright © 2024 Sonstein and Hommel. This is an open-access publication distributed under the terms of the Creative Commons Attribution license.

may be resource intensive for the expert, the model can be sustained long-term by recipients of the education. Additionally, this model allows curricula to be adapted across multiple health professions, learner levels, and clinical specialties to reach all different areas of an organization.

We describe how a train-the-trainer QI workshop can be an effective means to improve faculty QI knowledge and confidence in QI teaching skills. There are several QI curricula available in *MedEdPORTAL*; however, none independently target faculty or utilize the train-the-trainer model.[13-17]

Methods

We developed a 2-hour interactive didactic workshop for faculty interested in overseeing didactic QI curricula and/or experiential QI projects. The course was intended for educators across health system disciplines but could also apply to health system administrators. Participants were not required to have background QI knowledge. The workshop was initially structured for in-person training of up to 10-15 participants in a venue that could facilitate small-group activities; however, the course could be adapted to virtual training using software permitting small-group breakout sessions.

The workshop used a train-the-trainer model to teach the following core concepts: QI methodologies (with a focus on plan-do-study-act cycles[18]), measurement, implementation, and scholarship. The workshop consisted of an instructional slide set (Appendix A) and five interactive small-group exercises with structured worksheets and facilitator guides (Appendices B-M). The slide set contained the following: educational objectives, a workshop timeline, an introduction to QI, six steps to teach QI, and techniques for facilitating learners through experiential QI projects. All exercises were intended to reinforce the QI concepts and to replicate a critique of one step in a learner's QI project. During the workshop, exercises were completed in small groups of three to four learners with dedicated time for debrief following each exercise. Each exercise could be adapted to fit the needs of any individual health care discipline or clinical department. The content was developed by two internal medicine physicians with prior, formal training in education and QI.

Exercise 1 (Appendix B, exercise; Appendix H, facilitator guide) focused on critique of a learner's aim statement and took approximately 10 minutes to complete. Two aim statements were provided within the exercise to allow separate groups to critique different ones. The facilitator guide listed teaching points for the debrief and illustrated ways the aim statements could be rewritten.

Exercise 2 (Appendix C, exercise; Appendix I, facilitator guide) covered stakeholder analysis and took 5-8 minutes to complete. The goal of this exercise was to reinforce how a stakeholder analysis could be used with learners as they built their interdisciplinary QI team.

Exercise 3a (Appendix D, exercise; Appendix J, facilitator guide) featured a learner-created flowchart with four question prompts for flowchart critique. Exercise 3b (Appendix E, exercise; Appendix K, facilitator guide) was a similar exercise featuring a learner-created fishbone (or Ishikawa) diagram. To conserve time, half of the participant small groups were assigned exercise 3a, and half were assigned exercise 3b. This exercise familiarized participants with these commonly used QI tools and exposed common learner mistakes when utilizing the tools. The exercise took 15 minutes to complete.

Exercise 4 (Appendix F, exercise; Appendix L, facilitator guide) reinforced core concepts in QI measurement and took 10 minutes to complete. Participants critiqued proposed metrics for an example QI project by applying their knowledge of types of QI metrics, expected data sources, and data presentation options.

Exercise 5 (Appendix G, exercise; Appendix M, facilitator guide) covered QI interventions with a focus on strength of interventions and managing technical and social change. This exercise was expected to be completed within 7 minutes.

Prior to or at the start of the workshop, all participants completed a preassessment (Appendix N) with three components: participant demographics (profession, years of QI educational experience, presence of formal educational training, and presence of formal QI training), a multiple-choice baseline QI knowledge test, and a participant self-assessment of skills in teaching QI. For the self-assessment, participants were asked to rate their level of confidence on a 3-point scale (1 = *Not Confident,* 2 = *Some Confidence,* 3 = *Very Confident*) in the following areas: building effective QI didactic materials, leading trainees in experiential QI projects, and appraising a QI proposal. The survey instrument was created by the authors as we were unable to identify an existing validated tool to assess an instructor's ability to teach QI. The assessment was emailed to attendees prior to the session; participants who were not able to complete the electronic assessment were allotted time to complete a written assessment at the onset of the educational session. Immediately after the workshop, participants completed a course evaluation (Appendix O) where they rated the workshop using a 5-point Likert scale (1 = *very poor,* 2 = *poor,* 3 = *good,* 4 = *very good,* 5 = *excellent*).

Copyright © 2024 Sonstein and Hommel. This is an open-access publication distributed under the terms of the Creative Commons Attribution license.

All participants were invited to complete a postassessment (Appendix P) at 1 month and 6 months postworkshop. The postassessment was emailed to participants and included the same QI knowledge test and self-rating of QI teaching skills as the preassessment. Open-ended questions also invited participants to share how they had been engaged with QI training since the workshop and what resources they needed to further their QI skills. Participants were strongly encouraged to complete the postassessments during the workshop and through electronic communication but were not incentivized to do so.

We summarized participant demographics as counts with percentages. The QI knowledge test is reported as median percentage correct and summarized at each time point (pre, 1 month post, 6 months post). Self-assessment ratings for each question are summarized at each time point by counts with percentages. Course evaluation ratings for each question are summarized by counts. When missing data were encountered, we applied a pairwise deletion approach.

This educational innovation was deemed exempt by the University of Texas Medical Branch Institutional Review Board in February 2023.

Results

We report results after two workshops with 23 participants. Demographics were completed for 20 participants (87%). Baseline knowledge assessments were completed by 18 participants (78%), and baseline self-assessments were completed by 17 participants (73%). Postassessments were completed by 11 participants (48%) at 1 month and by nine participants (39%) at 6 months. All participants completed the course evaluation.

Participant demographics are summarized in Table 1. Of the attendees, five (26%) were physicians, eight (42%) were nurses, and three (16%) were health system administrators. Sixteen participants (80%) reported formal educational training, but only eight (40%) reported formal QI training. Six participants (32%) had no prior experience working in QI, and nine participants (47%) reported less than 5 years of experience. Only four participants (21%) reported more than 5 years of QI experience.

Baseline median QI knowledge test percentage correct was 36%, which increased to 77% 1 month postworkshop and 57% 6 months postworkshop. Participants' self-assessment ratings of their skills in teaching QI are summarized for each question in Table 2. Regarding the ability to build effective QI didactic materials, 11 participants (65%) rated themselves as not confident at baseline, while only two participants (12%) rated themselves as very confident. At the 6-month follow-up, zero participants (0%) rated themselves as not confident, and four participants (44%) rated themselves as very confident. We identified similar trends in self-assessment ratings for ability to lead trainees in experiential QI projects and ability to appraise a QI proposal, though fewer participants rated themselves as not confident at baseline for these teaching areas.

Table 1. Participants' Demographics (*N* = 20)

Demographic	No.	%
Participant profession[a]		
MD	5	26
RN	8	42
PhD	2	11
Allied health	1	5
Health system administrator	3	16
Years of QI experience[a]		
None	6	32
1-5 years	9	47
6-10 years	3	16
>10 years	1	5
Formal educational training		
No	4	20
Yes	16	80
Formal QI training		
No	12	60
Yes	8	40

Abbreviation: QI, quality improvement.
[a]Profession and years of QI experience were missing for one participant.

At the 6-month follow-up, participants were invited to explain how they had engaged in QI work since completing the workshop. All nine respondents expressed some level of engagement, with four participants (44%) reporting initiation or facilitation of a QI project, three participants (33%) reporting modifications to existing QI curricula, and four participants (44%) reporting mentoring learners in QI projects.

During the course evaluation, 21 of 23 participants (91%) rated the course overall as very good or excellent (Figure). We received similar ratings for workshop organization (item 1), statement of objectives (item 2), and covered topics meeting expectations (item 3). Ratings were slightly lower for helpfulness of handouts (item 4) and helpfulness of exercises (item 5), with very good or excellent ratings received by 20 of 23 participants (87%) and 18 of 23 participants (78%), respectively.

Discussion

A brief train-the-trainer interactive workshop can effectively enhance faculty readiness to oversee didactic QI curricula and experiential QI projects. We demonstrated improved

Copyright © 2024 Sonstein and Hommel. This is an open-access publication distributed under the terms of the Creative Commons Attribution license.

MedEdPORTAL® The AAMC Journal of Teaching and Learning Resources

Table 2. Self-Ratings of QI Teaching Skills

Skill[a]	No. (%)		
	Not Confident	Some Confidence	Very Confident
1. Building effective QI didactic materials for your learners			
Baseline	11 (65)	4 (23)	2 (12)
1 month post	3 (28)	4 (36)	4 (36)
6 months post	0 (0)	5 (56)	4 (44)
2. Leading trainees in experiential QI learning, including project development, facilitation, and scholarly presentations			
Baseline	9 (53)	6 (35)	2 (12)
1 month post	3 (27)	5 (46)	3 (27)
6 months post	0 (0)	4 (44)	5 (56)
3. Appraising a QI proposal as regards to project aim, measures, chosen intervention, and implementation strategies			
Baseline	7 (41)	8 (47)	2 (12)
1 month post	3 (27)	5 (46)	3 (27)
6 months post	0 (0)	4 (44)	5 (56)

Abbreviation: QI, quality improvement.
[a]Baseline self-ratings are reported for 17 participants. Self-ratings at 1 month are reported for 11 participants. Self-ratings at 6 months are reported for nine participants.

faculty QI knowledge and self-rated QI teaching skills among a multidisciplinary cohort at 1 month and 6 months postworkshop. This model can be developed by one to two local QI experts and adapted for efficient distribution throughout an institution.

This analysis includes participants from trainings offered through the Office of Educational Development, which reached faculty in the School of Nursing, School of Medicine, Graduate School of Biomedical Sciences, and School of Allied Health. Health system administrators within the quality department were also invited to attend. Subsequently, the workshop has been tailored and delivered to the following: nurse educators for master's or doctoral nursing students, physician educators for undergraduate medical students, and academic physicians in graduate medical education. We have provided training for faculty in graduate medical education across specialties using this train-the-trainer model but have also modified the resources for individual medical and surgical specialties using a faculty/resident co-training model. In all circumstances, we have adapted the materials with ease and maintained the 2-hour interactive workshop delivery strategy.

Curricular leaders and project mentors have been repeatedly identified as necessary components of QI training.[6-8] Universally required training elements include a focus on QI methodology, how to choose a project, stakeholder engagement, measurement, implementation, and scholarship. While these are universal elements, tailoring the instruction on methodology to

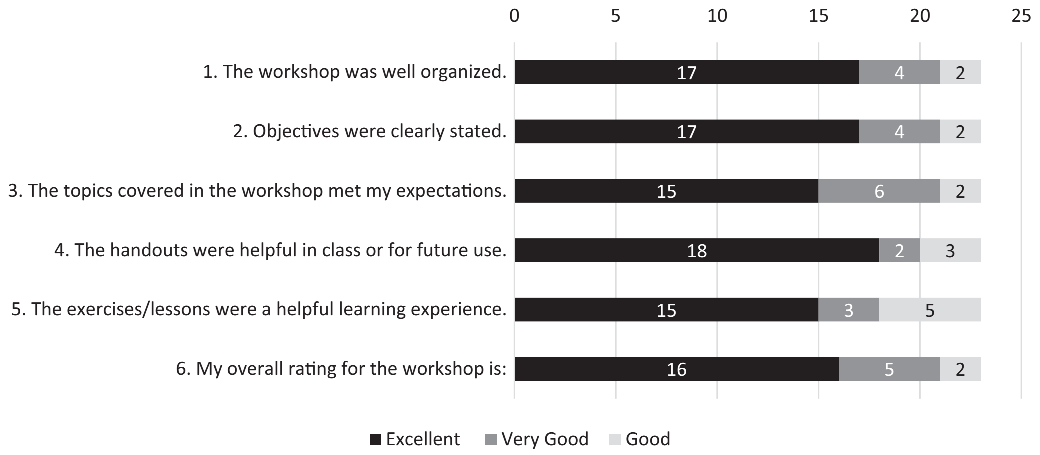

Figure. Participants' course evaluation ratings. Immediately after the workshop, participants completed a course evaluation where they rated each item using a 5-point Likert scale (1 = *very poor,* 2 = *poor,* 3 = *good,* 4 = *very good,* 5 = *excellent*). Course evaluations are shown for 23 participants. Results are reported as raw numbers per rating per item.

Copyright © 2024 Sonstein and Hommel. This is an open-access publication distributed under the terms of the Creative Commons Attribution license.

that of the local health system can facilitate learner engagement in health system improvement initiatives.[8] Additionally, involving faculty mentors specific to the discipline or clinical specialty of the learners increases effectiveness of the training by permitting trainees to recognize the application of their new knowledge.[8] The training materials provided here incorporate these necessary required training elements. Yet the slide set can be easily adapted to the local health system's methodology, and the exercise examples can be easily manipulated to a specific discipline or clinical specialty.

Requirements for QI training across disciplines and levels of education have now existed for several years. More faculty are entering the workforce with some prior QI training. While these materials were established to increase faculty QI knowledge and confidence to engage in QI education, we believe our workshop can serve to level-set faculty new to QI education at a given institution regardless of their prior QI experience. Our course can serve as either a stand-alone introduction to QI education or a supplemental overview for a more in-depth QI training experience.

There are several limitations to our work. Participants within the described cohort volunteered for our course. Their feedback may not reflect the experience of participants mandated to attend. We also used a nonvalidated pre- and postassessment tool. This was necessary given the lack of a validated tool for assessing an instructor's ability to teach QI, but it also limits the reliability of the results. Reliability of our results is further limited by the small size of the studied cohort. The total cohort included 23 participants, but only nine completed the 6-month follow-up. The positive trends demonstrated for the QI knowledge posttest and postworkshop self-assessment ratings should be interpreted with caution. Not only was the sample size small but the respondents at 6 months were likely the most engaged participants, contributing a sampling bias to our results. Importantly, while survey response rates were maintained over 39% throughout this study, investigators found it difficult to reliably resurvey at the 1-month and 6-month time periods. Users should consider both resources required and logistics for assessing participants at future time points if they desire to monitor how results are sustained over time.

QI education is likely to remain an important part of clinical training for the foreseeable future. All institutions need to identify or invest in the creation of a small cadre of QI experts. The resource we provide can be used as a catalyst to magnify the expertise of these select faculty and generate a larger cohort of educators prepared to mentor learners in their QI journey.

We believe these materials are simple yet comprehensive and structured yet adaptable.

Appendices

A. Train-the-Trainer Slide Set.pptx
B. Exercise 1 Aim Statements.docx
C. Exercise 2 Stakeholder Analysis.docx
D. Exercise 3a Flowchart Critique.docx
E. Exercise 3b Fishbone Critique.docx
F. Exercise 4 Measures Critique.docx
G. Exercise 5 Intervention Critique.docx
H. Exercise 1 Aim Statements Facilitator Guide.docx
I. Exercise 2 Stakeholder Analysis Facilitator Guide.docx
J. Exercise 3a Flowchart Critique Facilitator Guide.docx
K. Exercise 3b Fishbone Critique Facilitator Guide.docx
L. Exercise 4 Measures Critique Facilitator Guide.docx
M. Exercise 5 Intervention Critique Facilitator Guide.docx
N. Train-the-Trainer Quality Preassessment.docx
O. Course Evaluation.docx
P. Train-the-Trainer Quality Postassessment.doc

All appendices are peer reviewed as integral parts of the Original Publication.

Lindsay Sonstein, MD: Associate Professor, Department of Internal Medicine, University of Texas Medical Branch; ORCID: https://orcid.org/0000-0003-4530-9418

Erin Hommel, MD: Associate Professor, Department of Internal Medicine, University of Texas Medical Branch; ORCID: https://orcid.org/0000-0003-1975-4008

Disclosures
None to report.

Funding/Support
None to report.

Ethical Approval
The University of Texas Medical Branch Institutional Review Board deemed further review of this project not necessary.

References

1. Dolansky MA, Moore SM. Quality and Safety Education for Nurses (QSEN): the key is systems thinking. *Online*

Copyright © 2024 Sonstein and Hommel. This is an open-access publication distributed under the terms of the Creative Commons Attribution license.

J Issues Nurs. 2013;18(3):Manuscript 1. https://doi.org/10.3912/OJIN.Vol18No03Man01

2. Association of American Medical Colleges. *Quality Improvement and Patient Safety Competencies Across the Learning Continuum*. Association of American Medical Colleges; 2019. Accessed June 11, 2024. https://store.aamc.org/downloadable/download/sample/sample_id/302/
3. *ACGME Common Program Requirements (Residency).* Accreditation Council for Graduate Medical Education; 2022. Accessed June 11, 2024. https://www.acgme.org/globalassets/PFAssets/ProgramRequirements/CPRResidency_2022_TCCv2.pdf
4. *Standards and Required Elements for Accreditation of Physical Therapist Education Programs*. Commission on Accreditation in Physical Therapy Education; 2022. Accessed June 11, 2024. https://www.capteonline.org/globalassets/capte-docs/capte-pt-standards-required-elements.pdf
5. Jones AC, Shipman SA, Ogrinc G. Key characteristics of successful quality improvement curricula in physician education: a realist review. *BMJ Qual Saf*. 2015;24(1):77-88. https://doi.org/10.1136/bmjqs-2014-002846
6. Brown A, Lafreniere K, Freedman D, et al. A realist synthesis of quality improvement curricula in undergraduate and postgraduate medical education: what works, for whom, and in what contexts? *BMJ Qual Saf*. 2021;30(4):337-352. https://doi.org/10.1136/bmjqs-2020-010887
7. Wong BM, Levinson W, Shojania KG. Quality improvement in medical education: current state and future directions. *Med Educ*. 2012;46(1):107-119. https://doi.org/10.1111/j.1365-2923.2011.04154.x
8. Lavin J, Kao LS. Building infrastructure to teach quality improvement. *Semin Pediatr Surg*. 2023;32(2):151283. https://doi.org/10.1016/j.sempedsurg.2023.151283
9. Teaching for Quality (Te4Q). Association of American Medical Colleges. Accessed June 11, 2024. https://www.aamc.org/what-we-do/mission-areas/medical-education/teaching-for-quality-certificate-program
10. SHM Quality Improvement Academy. Society of Hospital Medicine. Accessed June 11, 2024. https://www.shmlearningportal.org/content/2022-shm-quality-improvement-academy#group-tabs-node-course-default1
11. Pronovost PJ, Holzmueller CG, Molello NE, et al; Armstrong Institute for Patient Safety and Quality Team. The Armstrong Institute: an academic institute for patient safety and quality improvement, research, training, and practice. *Acad Med*. 2015;90(10):1331-1339. https://doi.org/10.1097/ACM.0000000000000760
12. Pearce J, Mann MK, Jones C, van Buschbach S, Olff M, Bisson JI. The most effective way of delivering a train-the-trainers program: a systematic review. *J Contin Educ Health Prof*. 2012;32(3): 215-226. https://doi.org/10.1002/chp.21148
13. Vinas EK, White AB, Rogers RG, Ridgeway JJ, Young AE. Developing best clinical practices through outcomes improvement: an ongoing quality improvement curriculum for faculty and residents. *MedEdPORTAL*. 2018;14:10676. https://doi.org/10.15766/mep_2374-8265.10676
14. Reardon CL, Hafer R, Langheim FJP, et al. A quality improvement curriculum for psychiatry residents. *MedEdPORTAL*. 2020;16:10870. https://doi.org/10.15766/mep_2374-8265.10870
15. Dulay M, Saxe JM, Odden K, et al. Promoting quality improvement in primary care through a longitudinal, project-based, interprofessional curriculum. *MedEdPORTAL*. 2020;16:10932. https://doi.org/10.15766/mep_2374-8265.10932
16. Keefer P, Orringer K, Vredeveld J, Warrier K, Burrows H. Developing a quality improvement and patient safety toolbox: the curriculum. *MedEdPORTAL*. 2016;12:10385. https://doi.org/10.15766/mep_2374-8265.10385
17. Stewart D, Lye C, Lopez M, Mothner B, Camp E, Vachani J. Engaging learners through modules in quality improvement and patient safety. *MedEdPORTAL*. 2016;12:10482. https://doi.org/10.15766/mep_2374-8265.10482
18. Langley GJ, Moen RD, Nolan KM, Nolan TW, Norman CL, Provost LP. *The Improvement Guide: A Practical Approach to Enhancing Organizational Performance*. 2nd ed. Jossey-Bass; 2009.

Received: October 3, 2023
Accepted: March 27, 2024
Published: July 16, 2024

Copyright © 2024 Sonstein and Hommel. This is an open-access publication distributed under the terms of the Creative Commons Attribution license.
