# Supplementary material for: Development of a Train-the-Trainer Quality Improvement Curriculum
Source: MedEdPORTAL. 2024 Jul 16;20:11425. doi: 10.15766/mep_2374-8265.11425 (PMC11249715; doi:10.15766/mep_2374-8265.11425)
Supplement: Supplementary file 1 — Train-the-Trainer Slide Set.pptxExercise 1 Aim Statements.docxExercise 2 Stakeholder Analysis.docxExercise 3a Flowchart Critique.docxExercise 3b Fishbone Critique.docxExercise 4 Measures Critique.docxExercise 5 Intervention Critique.docxExercise 1 Aim Statements Facilitator Guide.docxExercise 2 Stakeholder Analysis Facilitator Guide.docxExercise 3a Flowchart Critique Facilitator Guide.docxExercise 3b Fishbone Critique Facilitator Guide.docxExercise 4 Measures Critique Facilitator Guide.docxExercise 5 Intervention Critique Facilitator Guide.docxTrain-the-Trainer Quality Preassessment.docxCourse Evaluation.docxTrain-the-Trainer Quality Postassessment.doc [file mep_2374-8265.11425-s001.zip › P. Train-the-Trainer Quality Postassessment.doc]

Train the Trainer Quality Post-Assessment

Knowledge Test: (highlight is the correct answer)

1. All of the following aim statements are appropriate EXCEPT ?
A. Reduce waiting time to see a physician in the Internal Medicine Residents’ Clinic to less than 15 minutes within 6 months.
B. Improve glucose control in patients in the Internal Medicine Clinic within 9 months.
C. Transfer 70% of patients admitted to the general surgery service from the Emergency Department to an inpatient room within one hour from admission over the next 6 months.
D. Decrease percentage of Adverse Drug Events in the Surgical Intensive Care Unit by 75% within the next 6 months.
2. What are the components of quality improvement?
A. Analysis of performance with systematic improvements to improve the performance
B. Use of methods to assure that a service or product confines to a desired standard
C. Compare one’s own performance to an external standard established as high performance
D. Process of assuring that a service or product meets a predefined set of criteria
3. Which of the following is NOT a Model for Improvement?
A. PDSA
B. Lean
C. Six Sigma
D. Root Cause Analysis
4. Which Quality Improvement tool helps to clarify an Understanding of the Current Process?
A. Ishikawa Diagram
B. Stake holder Analysis
C. Force field analysis
D. Run Chart
E. None of the above
5. You are doing a QI project on improvement of flu vaccination rates in your clinic. Your intervention is to have the medical assistant do an assessment of flu vaccine status and if the patient has not received the vaccine, they will educate the patient on the vaccine. Which of the following is a balancing metric you will need to follow during this project?
A. Flu Vaccination Rate
B. Rooming times
C. Number of flu assessments performed
D. None of the above
6. Using the same flu vaccination quality improvement project above, which quality improvement intervention is likely to be LEAST effective?
A. Creating a new policy that mandates the new flu assessment
B. Educating the staff about the new flu assessment and education process
C. Creating the flu assessment in the electronic health record with a hard stop ensuring completion
D. Creating a paper flu assessment for the staff to complete with each patient
7. Which of the following is NOT a reason quality improvement projects fail?
A. Changing things rapidly
B. Managing the social change
C. Under-training the multidisciplinary team
D. Expecting your implementation to last without iterations of the project

For the following questions, rate your confidence in the following items related to teaching quality improvement:

Confidence Level: 1 = Not Confident 2 = Some Confidence 3 = Very Confident

| 8. Building effective QI didactic materials for your learners | 1 | 2 | 3 |
| --- | --- | --- | --- |
| 9. Leading trainees in experiential QI learning including project development, facilitation, and scholarly presentations | 1 | 2 | 3 |
| 10. Appraising a QI proposal as regards to project aim, measures, chosen intervention and implementation strategies | 1 | 2 | 3 |

Please describe any QI work that you have been engaged with since the completion of the workshop.

What additional resources are necessary to help you further expand your QI knowledge and skills?
